# The GRAS protein RAM1 interacts with WRI transcription factors to regulate plant genes required for arbuscule development and function

**DOI:** 10.1073/pnas.2427021122

**Published:** 2025-05-19

**Authors:** Michael Paries, Karen Hobecker, Sofia Hernandez Luelmo, Filippo Binci, Angelica Guercio, Annika Usländer, Catarina Cardoso, Yang Si, Lotta Wankner, Sagar Bashyal, Philip Troycke, Franziska Brückner, Priya Pimprikar, Nitzan Shabek, Caroline Gutjahr

**Affiliations:** ^a^Plant Genetics, TUM School of Life Sciences, Technical University of Munich (TUM), 85354 Freising, Germany; ^b^Max-Planck-Institute of Molecular Plant Physiology, Postdam Science Park, 14476 Potsdam-Golm, Germany; ^c^Department of Plant Biology, College of Biological Sciences, University of California–Davis, Davis, CA 95616; ^d^Faculty of Biology, Genetics, Ludwig Maximilians University of Munich (LMU), 82152 Martinsried, Germany

**Keywords:** symbiosis, arbuscular mycorrhiza, transcriptional regulation, GRAS transcription factor, root

## Abstract

During arbuscular mycorrhiza (AM) symbiosis AM fungi form tree-shaped structures called arbuscules in root cortex cells of host plants. Arbuscules and their host cells are central for reciprocal nutrient exchange between the symbionts. *REQUIRED FOR ARBUSCULAR MYCORRHIZATION1* (*RAM1*) encodes a GRAS protein crucial for transcriptionally regulating plant genes needed for arbuscule development and nutrient exchange. Similar to other GRAS proteins, RAM1 likely does not bind to DNA and how RAM1 activates its target promoters remained elusive. Here, we demonstrate that RAM1 interacts with five AM-induced APETALA 2 (AP2) transcription factors of the WRINKLED1-like family called CTTC MOTIF-BINDING TRANSCRIPTION FACTOR1 (CBX1), WRI3, WRI5a, WRI5b, and WRI5c via a C-terminal domain containing the M2/M2a motif. This motif is conserved and enriched in WRI proteins encoded by genomes of AM-competent plants. RAM1 together with any of these WRI proteins activates the promoters of genes required for symbiotic nutrient exchange, namely *RAM2*, *STUNTED ARBUSCULES (STR),* and *PHOSPHATE TRANSPORTER 4 (PT4)*, in *Nicotiana benthamiana* leaves. This activation as well as target promoter induction in *Lotus japonicus* hairy roots depends on *MYCS* (*MYCORRHIZA SEQUENCE*)-elements and *AW*-boxes, previously identified as WRI-binding sites. The *WRI* genes are activated in two waves: Transcription of *RAM1*, *CBX1,* and *WRI3* is coregulated by calcium- and calmodulin-dependent protein kinase-activated CYCLOPS, through the *AMCYC-RE* in their promoter, and DELLA, while *WRI5a*, *b,* and *c* promoters contain *MYCS*-elements and *AW*-boxes and can be activated by RAM1 heterocomplexes with CBX1 or WRI3. We propose that RAM1 provides an activation domain to DNA-binding WRI proteins to activate genes with central roles in AM development and function.

## Significance

Arbuscular mycorrhiza (AM) symbiosis is formed by soil-inhabiting Glomeromycotina fungi and most land plants and can significantly promote plant performance via enhanced nutrient uptake. The fungi form branched hyphal structures, the arbuscules, within root cortex cells that provide the major interface for nutrient exchange. Coordination of arbuscule formation and reciprocal nutrient exchange require complex transcriptional changes in colonized cells. The protein REQUIRED FOR ARBUSCULAR MYCORRHIZATION 1 (RAM1) is important for transcriptional regulation in arbuscule-containing cells but predicted not to bind DNA. We demonstrate that RAM1 interacts with five DNA-binding AM-induced WRI transcription factors to activate plant genes involved in nutrient exchange, arbuscule formation, and maintenance. This provides important mechanistic insights into the regulation of an ecologically important plant-fungal symbiosis.

Arbuscular mycorrhiza (AM) is a mutualistic symbiosis formed between land plants and fungi of the phylum glomeromycotina (1, 2). A hallmark of this alliance is the reciprocal exchange of nutrients: The plant obtains water and mineral nutrients, in particular phosphate and nitrogen, and the fungus receives essential organic carbon sources, in the form of lipids and hexoses (3, 4). The majority of this trade most likely occurs at the interface between arbuscule-containing root cortex cells and arbuscules, structures resulting from repeated bifurcate branching and tapering of fungal hyphae within root cortex cells. The arbuscules are surrounded by a plant-derived periarbuscular membrane, thought to harbor a manifold arsenal of transporters and other proteins, crucial for plant-fungal nutrient exchange (5–7).

During arbuscule development, cell-autonomous transcriptional and subcellular changes occur in affected plant cells and their coordination involves complex transcriptional regulation (8, 9). The GRAS transcription factor [named after the first three members GIBBERELLIC ACID INSENSITIVE (GAI), REPRESSOR of GAI (RGA) and SCARECROW (SCR)] REQUIRED FOR ARBUSCULAR MYCORRHIZATION 1 (RAM1) is required for arbuscule maturation and the formation of fine branches. Mutation of *RAM1* results in a severe impairment of arbuscule branching and overall root colonization (10–14). It regulates the expression of genes encoding enzymes involved in lipid biosynthesis and transporters required for reciprocal nutrient exchange during AM symbiosis (10–14). Thus, RAM1 plays a central role in regulating symbiosis development and function.

Transcription of *RAM1* itself is induced during root colonization by AM fungi, and is regulated by a complex of CYCLOPS and the proteolytic target of gibberellic acid (GA) signaling DELLA (14–19). CYCLOPS activates the *RAM1* promoter at the palindromic *AMCYC*-response element (RE) (14) and needs to be phosphorylated by the calcium- and calmodulin-dependent protein kinase (CCaMK) (14, 17) to fulfill this role. CCaMK, CYCLOPS, and DELLA are part of the common symbiosis signaling network operating during AM and in legumes also in root nodule (RN) symbiosis with nitrogen-fixing rhizobia (15–17, 19, 20). The finding that CYCLOPS and DELLA directly interact demonstrated that common symbiosis signaling integrates hormone stimuli, which have the potential to alter symbiosis development in accordance with the physiological state of the plant.

Two studies suggest direct interaction of GRAS proteins with DNA in electromobility shift assays (EMSAs) (21, 22), while the majority show that GRAS transcription factors do not bind to DNA, but regulate gene expression via the interaction with other (DNA-binding) transcriptional regulators (23–25). For example, the GRAS proteins SHORTROOT and SCARECROW and DELLAs interact with DNA-binding C_2_H_2_ zinc finger transcription factors of the INDETERMINATE DOMAIN family to regulate genes involved in plant development (26–28); and DELLAs transcriptionally activate genes in AM and RN symbiosis by interacting with CYCLOPS or MYB1 (14, 19, 29). How RAM1, a central regulator of arbuscule development and function, interacts with the DNA to induce expression of its target genes, remained elusive.

Transcriptomics in *Medicago truncatula* revealed that in addition to other transcription factors such as *RAM1*, five genes encoding AP2/ETHYLENE RESPONSE FACTOR (ERF) transcription factors and belonging to the WRINKLED1-like (WRI1-like) family are induced in mycorrhizal roots (30). Proteins of the WRI1-like family were first described to be regulators of lipid synthesis in *Arabidopsis thaliana* (31–33). Among the five AM-induced WRI encoding genes (30), the protein products of *CTTC MOTIF-BINDING TRANSCRIPTION FACTOR1* (*CBX1*) in **Lotus* japonicus* and *WRI5a* in *M. truncatula* were shown to bind to promoters of *PHOSPHATE TRANSPORTER 4* (*PT4*) and *STUNTED ARBUSCULES* (*STR*) via so-called *MYCS*-elements and *AW*-boxes (34–37). These genes encode important transporters required for phosphate and lipid transfer, respectively, across the periarbuscular membrane and for arbuscule branching and maintenance (38, 39). Chromatin immunoprecipitation of CBX1 plus sequencing revealed that it also binds to promoters of AM-specific lipid biosynthesis genes, such as the glycerol-3 phosphate acyl transferase gene *REQUIRED FOR ARBUSCULAR MYCORRHIZATION 2* (*RAM2*) (34, 40, 41). Despite their role in regulating important genes required for nutrient exchange, mutation of *CBX1* and RNAi-mediated knockdown of *WRI5a*, *b,* and *c* leads to a relatively mild phenotype with slightly reduced colonization and a slight increase in the number of stunted arbuscules (for *WRI5a*) (34, 35). This is likely due to redundancy with (any of) the other four AM-induced WRI transcription factors and/or additional transcriptional regulators because mutation of the single *WRI* gene encoded in the genome of the liverwort *Marchantia paleacea* results in abortion of AM development (42). WRI quintuple mutants or mutants of higher order are currently not available in legumes.

In *M. truncatula* the expression of AM-specific lipid biosynthesis genes such as *RAM2* as well as *STR* and *PT4* depends on *RAM1* and ectopic expression of *RAM1* in *M. truncatula* and *L. japonicus* induces their expression in the absence of the fungus (11, 14, 30, 43). This overlap in transcriptional regulatory targets of RAM1 and CBX1/WRI5a led us to hypothesize that RAM1 may activate target promoters by physically interacting with the AM-induced WRI transcription factors that may act as adaptors for DNA binding.

## Results

### RAM1 and AM-Induced WRI Transcription Factors Coregulate the Promoters of *RAM2* and *STR*.

**Fig. 1.**
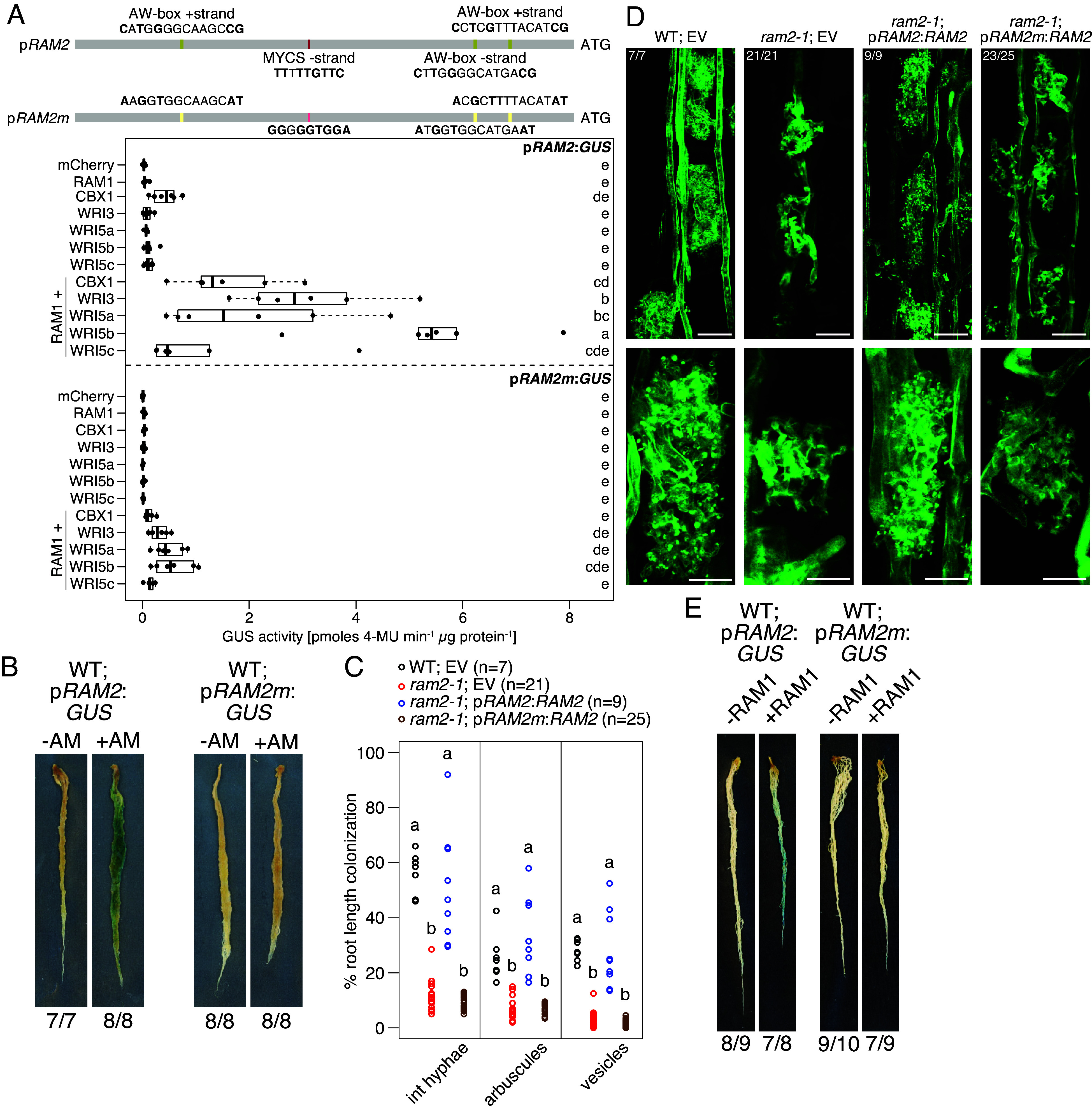
WRI-target sites in the *RAM2* promoter are required for *RAM2* activation by RAM1 and for arbuscule development. (*A*) Transactivation assay in *N. benthamiana* leaves. Genomic sequence encoding proteins indicated at the *y*-axis were controlled by the *LjUbiquitin10* promoter. The schematic representation on *Top* illustrates position and sequence of *AW*-boxes (yellow marks) and *MYCS*-elements (red marks) and their mutated versions (light yellow marks and light red marks, respectively). Bold letters indicate the basepairs, defining the respective motif. 4-MU, 4-methylumbelliferone. Bold black line, median; box, interquartile range; whiskers highest and lowest data point within 1.5 interquartile range; dots, actual values. Different letters indicate different statistical groups (ANOVA; post hoc Tukey; n = 6; *P* < 0.05). The experiment was performed twice with similar results. (*B*) Representative images of GUS activity resulting from p*RAM2* and p*RAM2m* activation in wild-type hairy roots colonized with *Rhizophagus irregularis* at 8 wpi. (*C*) Root length colonization parameters of wild-type and *ram2-1* hairy roots, colonized with *R. irregularis* at 8 wpi, transformed with the indicated expression cassettes. Different letters indicate different statistical groups (ANOVA; post hoc Tukey; *P* < 0.05). (*D*) Representative laser scanning confocal images of wild-type and *ram2-1* hairy roots colonized by *R. irregularis* at 8 wpi, transformed with the indicated expression cassettes. Numbers indicate the root systems that displayed the phenotype shown in the image, among the number of analyzed root systems. The fungus is stained with wheat germ agglutinin (WGA)-Alexa-Fluor488. Scale bars: *Upper* panel 20 µm, *Lower* panel 10 µm. (*E*) Representative images of GUS activity resulting from p*RAM2* and p*RAM2m* activation in noncolonized wild-type hairy roots at 6 wpp, coexpressing p*Ubi*:*RAM1* (+RAM1) or not (−RAM1). (*B* and *E*) Roots were stained with X-Gluc for 6 h. Numbers indicate the number of root systems that displayed staining as shown in the image, among the total number of analyzed root systems. (*B*–*E*), the experiments were performed once.

We examined this hypothesis using transactivation assays in *Nicotiana benthamiana* leaves with RAM1 in combination with each of the five AM-induced WRI transcription factors CBX1, WRI3, and WRI5a, b, and c. We chose the promoters of *RAM2* and *STR* as examples for target promoters. Both promoters drive genes required for plant lipid provision to AM fungi (30, 44, 45). RAM1 or any of the five AM-induced WRI transcription factors expressed alone was insufficient to significantly increase ß-glucuronidase (GUS) activity relative to the negative control mCherry when the *GUS* gene was driven by the *RAM2* promoter (Fig. 1*A*). A small and not statistically significant increase of GUS activity was caused by expression of CBX1 (see also ref. 34). However, when we coexpressed RAM1 together with any of the WRI proteins, the GUS activity increased significantly and only the induction by the combination of RAM1 with WRI5c was not significantly different from the controls (Fig. 1*A*). We conclude that RAM1 and four of the WRIs act synergistically to activate the *RAM2* promoter in *N. benthamiana* leaves. We identified known WRI-binding sites (34, 35) one putative *MYCS*-element and three *AW*-boxes in the 2,200 bp long *RAM2* promoter fragment employed for the transactivation assay (Fig. 1*A*). When we mutated all of them, the promoter activity was drastically reduced, in the presence of any of the tested transcription factor combinations (Fig. 1*A*), indicating RAM1 can activate these promoters only when the WRI proteins can bind to their cognate *cis*-elements. Similar results were obtained with the promoter sequence of *STR* (2,150 bp upstream of start codon), in which we found conservation of the two *AW*-boxes described in the *M. truncatula STR* promoter (35), along with one additional putative *MYCS*-element (*SI Appendix*, Fig. S1*A*). However, the *STR* promoter responded slightly to RAM1 alone and this could not be abolished by mutating the *AW*-boxes and the *MYCS*-element (*SI Appendix*, Fig. S1*A*), indicating that RAM1 may interact with an additional transcription factor present in *N*. *benthamiana* leaves that may bind to an unknown *cis*-element in the promoter fragment.

The *RAM2* and *STR* promoters displayed variation in the magnitude of their response to different WRI-RAM1 combinations: *RAM2* promoter activity was strong with all WRIs in combination with RAM1, except for WRI5c (Fig. 1*A* and *SI Appendix*, Fig. S2*A*), while *STR* promoter activity was strongest in the presence of CBX1 and WRI3, combined with RAM1 (*SI Appendix*, Fig. S1*A*). To investigate whether these differences are caused by a different abundance or position of *AW*-boxes in the examined promoter sequences, we assembled versions of the *RAM2* promoter, in which either the *MYCS*-element or the *AW*-boxes were mutated (*SI Appendix*, Fig. S2*A*). When only the *AW*-boxes were mutated, the *RAM2* promoter showed low activity similarly to the version in which all WRI binding sites were mutated (Fig. 1*A* and *SI Appendix*, Fig. S2*A*). Mutation of the *MYCS*-element alone hardly influenced the promoter activity caused by WRI3, WRI5a, or WRI5b, while the activity caused by CBX1 dropped (*SI Appendix*, Fig. S2*A*), suggesting that in this promoter context the *MYCS*-element is preferentially bound by CBX1, while all WRI transcription factors activate the *RAM2* promoter majorly via *AW*-boxes (*SI Appendix*, Fig. S2*A*). We performed the same experiment using the promoter of the phosphate transporter gene *PT4*, as it contains two *MYCS*-elements and three *AW*-boxes in a different arrangement. The *PT4* promoter was strongly activated by the combination of RAM1 with all WRIs except WRI3 (*SI Appendix*, Fig. S2*B*), congruent with previously performed EMSA experiments (34). Unlike what we observed for the *RAM2* promoter, mutation of the *MYCS*-element in the *PT4* promoter affected activation by WRI5a and RAM1, while mutation of the *AW*-boxes affected activation by WRI5a and WRI5c in combination with RAM1. Only mutation of all *MYCS*-elements and *AW*-boxes caused a loss of activation by all transcription factor combinations (*SI Appendix*, Fig. S2*B*). It appears that at least in transactivation assays in *N. benthamiana* leaves the WRIs cannot be specifically assigned to any of the two elements. Potentially, the differences in transactivation by different WRI-RAM1 combinations are determined by the sequence context in which the *cis*-elements reside (e.g., unknown *cis*-elements, which are bound by additional transcription factors), the distance among *cis*-elements and/or their distance to the transcriptional start site.

To understand whether the synergistic action of RAM1 with the WRI transcription factors is required for expression of *RAM2* and *STR* in the context of symbiosis, we examined whether *AW*-boxes and *MYCS*-elements are required for promoter activation during AM and for AM formation. Colonized *L. japonicus* wild-type hairy roots transformed with p*RAM2*:*GUS* turned blue during GUS staining while noninoculated roots remained white, indicating strong *RAM2* promoter activity upon colonization (Fig. 1*B*), as described before (44). Roots transformed with mutated p*RAM2m*:*GUS* did not display blue staining, irrespective of the symbiotic state of the root (Fig. 1*B*), indicating that *AW*-boxes and *MYCS*-elements are required for p*RAM2* activity in colonized roots. This was confirmed by complementing a *ram2* mutant with the wild-type *RAM2* gene driven by p*RAM2* or p*RAM2m*. The wild-type *RAM2* promoter drove *RAM2* expression sufficiently to restore wild-type-like root colonization and arbuscule maturation in the *ram2* background (Fig. 1 *C* and *D*). The promoter sequence lacking all WRI binding sites failed to restore AM in *ram2* roots (Fig. 1 *C* and *D*). These results were confirmed with the *STR* promoter and transgenic complementation of the *str* mutant (*SI Appendix*, Fig. S1 *B*–*D*).

Previously we and others showed that ectopic expression of *RAM1* can activate target genes in the absence of root colonization (11, 14, 30, 43). We examined whether activation of promoters by ectopic expression of *RAM1* depends on WRI binding sites. We transformed *L. japonicus* hairy roots with Golden Gate constructs containing an expression cassette with the *RAM1* gene fused to the *LjUbiquitin10* promoter and an additional cassette containing the *GUS* gene fused to p*RAM2* or p*STR* as well as to their mutated versions. The empty vector (EV) cassette lacking p*Ubi*:*RAM1* but containing the p*RAM2*:*GUS* cassette caused only sparse GUS-activity in a subset of root systems, irrespective of the promoter version (Fig. 1*E* and *SI Appendix*, Fig. S1*E*). In contrast, in the presence of p*Ubi*:*RAM1,* the *RAM2* and *STR* promoters were strongly activated as indicated by blue staining, while the roots transformed with the constructs containing the mutated *RAM2* and *STR* promoter sequences remained white (Fig. 1*E* and *SI Appendix*, Fig. S1*E*). Thus, RAM1 can only activate the *RAM2* and the *STR* promoters, when these promoters can be bound by the WRI transcription factors.

### RAM1 Interacts with AM-Induced WRI Transcription Factors via its GRAS Domain.

**Fig. 2.**
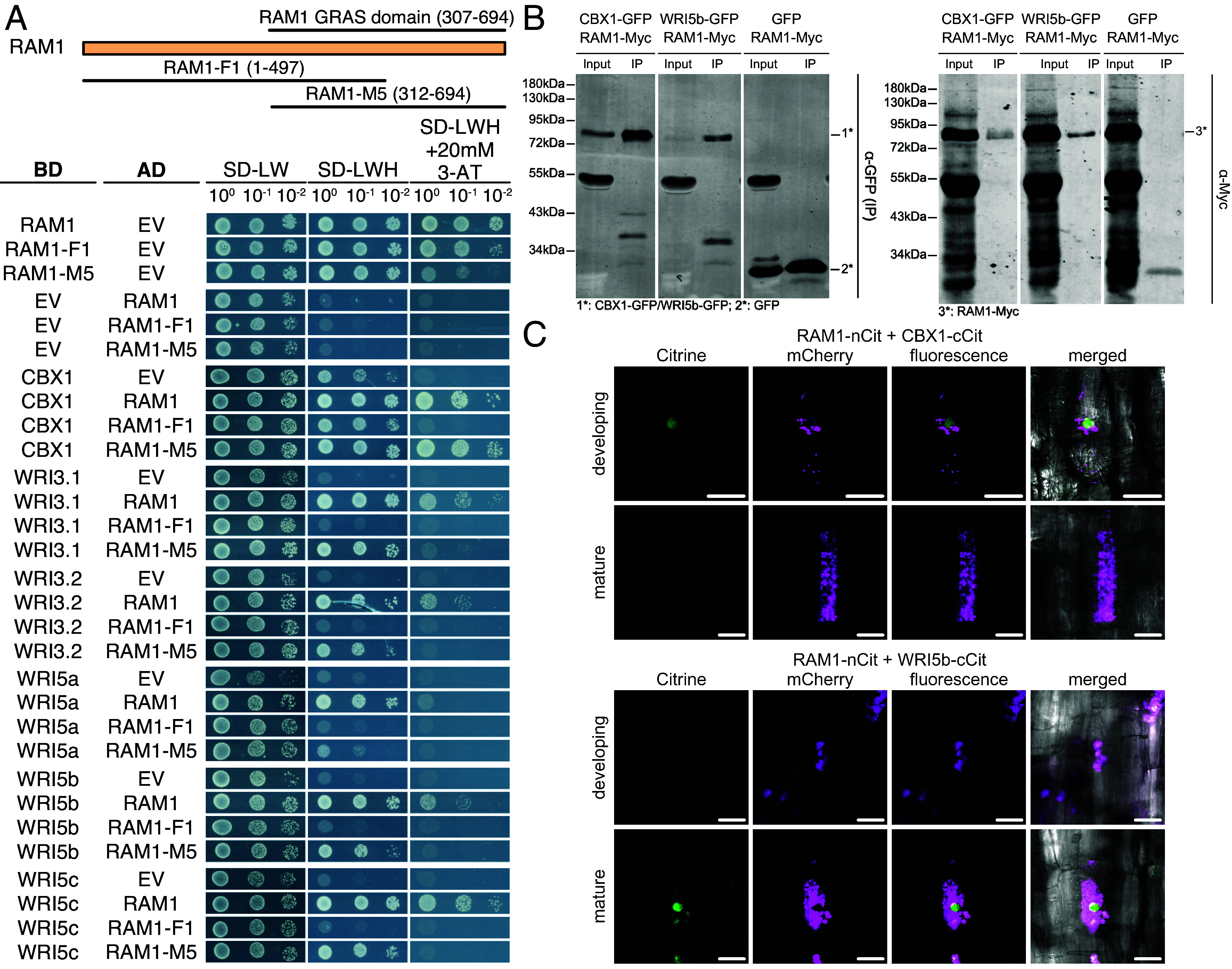
RAM1 interacts with AM-induced WRI transcription factors via its GRAS domain. (*A*) GAL4-based Y2H assay to test interaction of RAM1 as prey (AD) and five AM-induced WRI transcription factors as bait (BD). The schematic representation on *Top* illustrates the truncated F1 and M5 RAM1 versions and their length in amino acids. Expressed proteins, optical density of dropped yeast cultures, amino acids lacking from the medium and 3-AT concentration are provided in the figure. (*B*) Western blot images of Co-IP assays showing interaction of CBX1 and WRI5b with RAM1 in *N. benthamiana* leaves. Fractions loaded are input (Input) and immunoprecipitation (IP). Molecular weights of the bands of the protein standard, left side. Primary antibodies used for detection, right side. (*A* and *B*) The experiments were performed twice with similar results. (*C*) Interaction of CBX1 or WRI5b with RAM1 based on BiFC in cortex cells of colonized *L. japonicus* hairy roots 5wpi. Green fluorescence indicates interaction. Magenta fluorescence indicates arbuscules. All gene fusions were expressed under the control of their endogenous (*RAM1*, *CBX1,* or *WRI5b*) promoters. See *SI Appendix*, Table S1 for the number of observed cells. Size bars, 20 µm. nCit, N-terminal half of Citrine; cCit, C-terminal half of Citrine.

As RAM1 and AM-induced WRIs target promoters synergistically and WRI-promoter binding sites are required for RAM1 activity, we hypothesized that RAM1 interacts directly with the WRI proteins. To address this, we performed a GAL4-based yeast two-hybrid (Y2H) assay (Fig. 2*A*). PCR amplification and cloning of the coding sequences of *CBX1*, *WRI3*, *WRI5a*, *b,* and *c* from *L. japonicus* complementary DNA (cDNA), revealed the presence of two splice variants of *WRI3*, which we termed *WRI3.1* and *WRI3.2*. Both variants were included in the assay. Yeast growth in the absence of histidine and in the presence of 3-aminotriazole (3-AT) indicates a strong interaction between full-length RAM1 (fused to the activation domain) with each of the five WRI proteins CBX1, WRI3, WRI5a, WRI5b, and WRI5c (Fig. 2*A*). The interaction of CBX1 and WRI5b with RAM1 was confirmed by coimmunoprecipitation (Co-IP) from transiently transformed *N. benthamiana* leaves (Fig. 2*B*), and by bimolecular fluorescence complementation (BiFC) in colonized *L. japonicus* hairy roots (Fig. 2*C*). As expected for the tested transcriptional regulators, the BiFC signal was detectable mainly in the nucleus of arbuscule-containing cells (Fig. 2*C*). However, the BiFC signal was observed only very rarely (*SI Appendix*, Table S1). This may be explained by a high turnover of RAM1, because so far, we did not succeed in microscopically observing fluorophore-tagged RAM1 in *L. japonicus* roots, when RAM1 was expressed under the control of its own promoter. Interestingly, in these rare cases, we observed BiFC signal indicating CBX1–RAM1 interaction, only in cells containing young, developing arbuscules, while we observed the rare BiFC signal resulting from WRI5b–RAM1 interaction only in cells containing mature arbuscules (Fig. 2*C*). These results could potentially reflect different arbuscule developmental stage-specific expression patterns of the endogenous promoters of *CBX1* and *WRI5b*. Due to the rare observation of BiFC signal, this conclusion needs to be considered preliminary.

By including truncated versions of RAM1 [Fig. 2*A*; nomenclature of RAM1-F1 and RAM1-M5 is in analogy to DELLA-F1 and DELLA-M5 (46)] into the Y2H assay, we found that the RAM1 GRAS domain is required for the interaction with the five WRI proteins, while the N-terminal part of RAM1 is dispensable (Fig. 2*A*).

To examine specificity, we tested by Y2H assay and Co-IP, whether the WRIs also interact with another GRAS protein DELLA1 which is known for its involvement in AM symbiosis (47) and closely related to RAM1 on a phylogenetic tree (14). None of the used methods suggested interaction between DELLA1 and any of the five WRI proteins, indicating that the interaction is rather specific for RAM1 (*SI Appendix*, Fig. S3 *A* and *B*).

### The M2/M2b Motif of CBX1 Participates in the Interaction with RAM1.

**Fig. 3.**
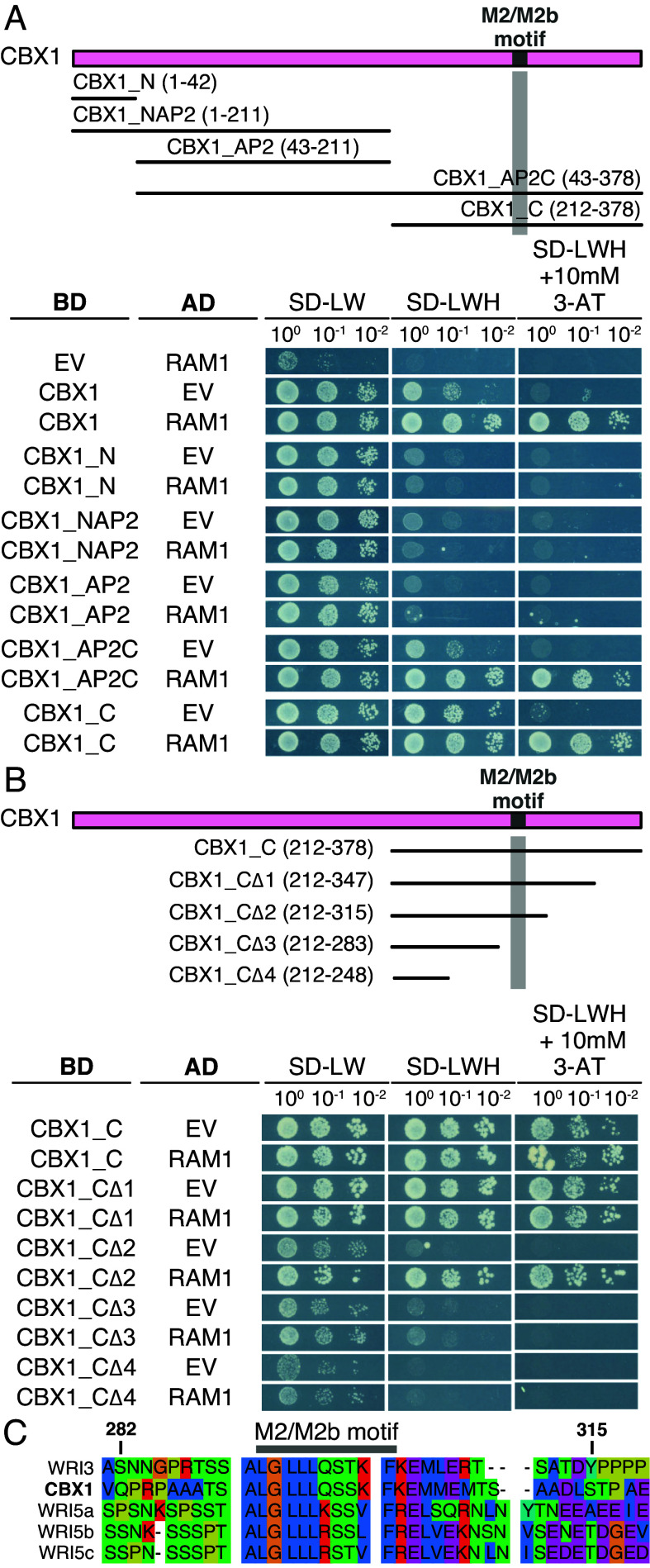
The conserved M2 motif in the C-terminus of AM-induced WRI transcription factors is important for the interaction with RAM1. (*A* and *B*) GAL4-based Y2H assay to test interaction of RAM1 as prey (AD) and CBX1 and its truncated versions as bait (BD). Schematic representations on *Top* illustrate the truncated versions of CBX1 and their length in amino acids. The experiments were performed twice with similar results. Truncations in (*A*) are based on (34). Truncations in (*B*) are based on the sequence of CBX1_C. All coding-sequence-containing plasmids were used in combination with the complementary EV as negative controls. Expressed proteins, optical density of dropped yeast cultures, amino acids lacking from the medium and 3-AT concentration are provided in the figure. (*C*) Alignment of the amino acid sequences of five AM-induced WRI transcription factors shown for CBX1 to be important for the interaction with RAM1 in (*B*). The color code indicates amino acids with similar physico-chemical properties (blue nonpolar, green polar, red basic, purple acidic, yellow proline, orange glycine). Dashes show gaps in the alignment. The alignment was performed in seaview5 (49) with the muscle algorithm (50). Alignment of full-length protein sequences is shown in *SI Appendix*, Fig. S4. Amino acid numbers on top of the alignment refer to the amino acid sequence of CBX1.

To pinpoint which region of the WRI proteins is important for the interaction with RAM1, truncated versions of CBX1 (34) as a representative for the WRI proteins, were generated and examined for interaction with RAM1 in the Y2H assay (Fig. 3*A*). The CBX1 C-terminus and the AP2 domains plus the C-terminus interacted with RAM1, similar to the full-length CBX1 protein (Fig. 3*A*). No interaction was observed for the N terminus, the AP2 domains, and the N terminus plus the AP2 domains (Fig. 3*A*). Therefore, the amino acid sequence allowing interaction with RAM1 must be located within the C-terminal region of CBX1. Further truncations of the CBX1 C-terminus narrowed down the interactive amino acid stretch to a region between amino acids Q283 and S315 (Fig. 3*B*; see *SI Appendix*, Fig. S3*C* for higher 3-AT concentrations). Removal of the amino acids from S315 to E347 strongly reduced the autoactivity of CBX1 in yeast (Fig. 3*B*). We hypothesized that the RAM1 interaction site of all five WRIs must be conserved among them. Alignment of their amino acid sequences revealed conserved stretches, present in all five protein sequences (Fig. 3*C* and *SI Appendix*, Fig. S4). Besides the two AP2 domains, another conserved stretch corresponds to the M2/M2b motif, which was previously computationally identified (48) (Fig. 3*C* and *SI Appendix*, Figs. S4 and S5*B*). This motif is located in the domain of CBX1 that is required for the interaction with RAM1 suggesting that it participates in the interaction (Fig. 3 *B* and *C* and *SI Appendix*, Fig. S4). To understand which domain of RAM1 may interact with this portion of CBX1, further truncations of RAM1-M5 were generated by deleting GRAS subdomains sequentially (24) from the N- and the C-terminus. These truncated RAM1 versions were tested for interaction with CBX1_CΔ2 (*SI Appendix*, Fig. S3*D*). However, none of them allowed yeast growth comparable to the full-length sequence of the RAM1 GRAS domain, suggesting that the intact structure of the entire RAM1 GRAS domain is required for this interaction.

To determine the phylogenetics distribution of the M2/M2b motif, WRI amino acid sequences from 39 plant species (*SI Appendix*, Table S6) were retrieved by BLAST, using the amino acid sequences of *L. japonicus* CBX1, WRI3, WRI5a, b, and c as input. Some WRI amino acid sequences contained a M2/M2b motif with one amino acid polymorphism with respect to the consensus sequence. We termed these motifs M2-like. A screen for the M2/M2b and M2-like motifs within the collected sequences revealed that these motifs occur in 18.5% of WRI proteins encoded by AM host genomes and only in 2.7% WRI proteins encoded by nonhost genomes (*SI Appendix*, Fig. S5*C* and Table S6). In a cladogram and phylogenetics tree the sequences containing M2/M2b/M2-like motifs, cluster predominantly in two clades: One consists mainly of sequences of AM host proteins including WRI5a, WRI5b, WRI5c, and their homologues (*SI Appendix*, Figs. S5*A* and S6). The other clade contains sequences of CBX1, WRI3, and their homologues as well as some sequences from AM nonhosts (*SI Appendix*, Figs. S5*A* and S6 and Table S5). This clade also features one additional amino acid sequence from *L. japonicus*, containing the motif, encoded by *LotjaGi3g1v0346300* (*SI Appendix*, Figs. S5*A* and S6). However, as this gene shows a decrease in transcript abundance upon progressing colonization with mycorrhizal fungi (51), it was not further investigated in this study. In summary, the overrepresentation of M2/M2b/M2-like domains in WRI proteins of AM hosts highlights the symbiotic function of this domain.

### Structural Models Support the Role for the M2/M2b Domain in WRI-Interaction with RAM1 and Accurately Predict Key Amino Acids.

**Fig. 4.**
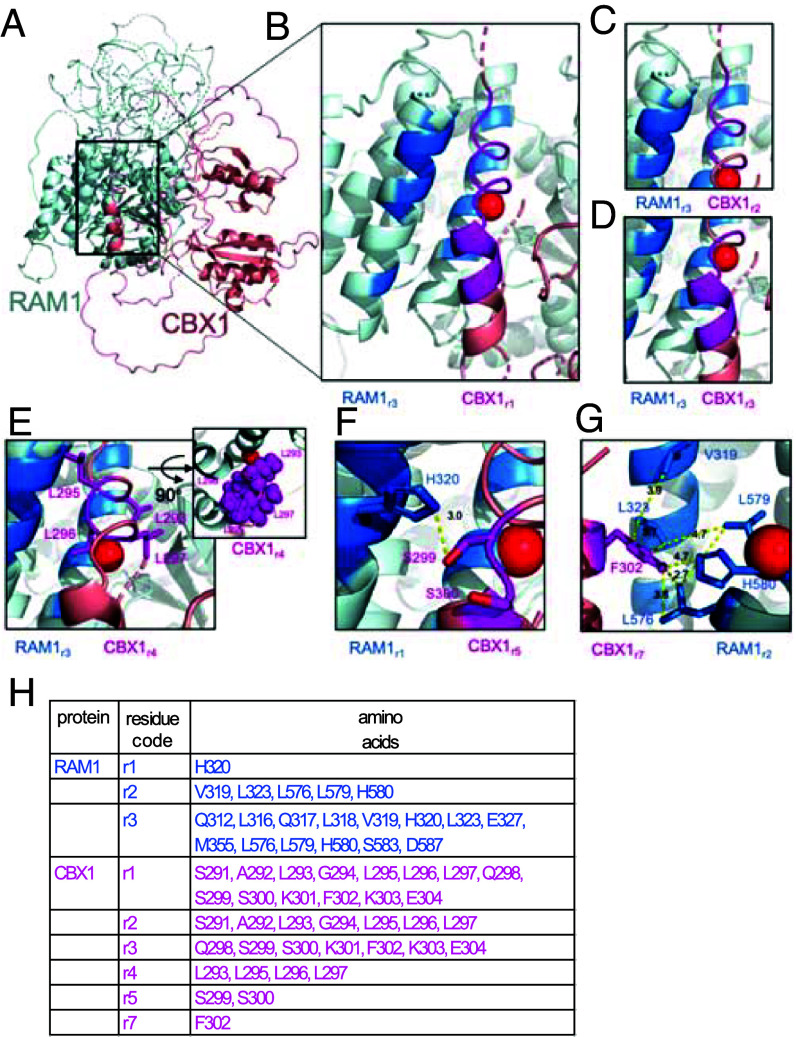
Structural implications of residues involved in the CBX1–RAM1 interaction (*A*) RoseTTafold-derived CBX1–RAM1 complex structure shown as cartoon. RAM1 is colored in light blue and CBX1 in salmon. (*B*) Close-up view of CBX1–RAM1 interacting helices. Residues in CBX1 interaction helix (r1) correspond to CBX1 291-304 SALGLLLQSSKFKE, which encompass the CBX1 M2/M2b domain and are colored in magenta. Residues highlighted in marine relate to RAM1 residues (r3) within 3 Å of CBX1 291-304 that may potentially form interactions. Red sphere is provided for spatial orientation. (*C*–*G*) Interacting residues are highlighted between CBX1 (magenta) and RAM1 (marine). The additional box in (*E*) represents the top view showing space filling by leucine residues as spheres. Measurements are shown in panels (*F* and *G*) to highlight (*F*) correct hydrogen bonding distance and (*G*) correct van der Waals distance network. (*H*) Table contains the codes of the amino acids of CBX1 and RAM1 that are highlighted in (*B*–*G*).

To generate independent support for the domain of WRI family proteins that interacts with RAM1 and generate hypotheses for the interaction interface, the WRI proteins CBX1 and WRI5b were used as representatives for the two subgroups (CBX1/WRI3 and WRI5a, b, c) and modeled in complex with RAM1 using RoseTTafold (52) (Fig. 4*A* and *SI Appendix*, Figs. S7 and S8). The top-ranked RosettaFold model, representing the predicted structure with the highest confidence, was used for further analysis. The interaction interface for the CBX1–RAM1 interactions involved the residues 290-310 in CBX1 and three helices in RAM1 corresponding to residues 315 to 330, 354 to 375, and 569 to 592 (Fig. 4*A* and *SI Appendix*, Fig. S7). The predicted involvement of a RAM1 helix bundle in the interaction, explains our failure to find the interacting domain in RAM1 through deletion series in Y2H assays (*SI Appendix*, Fig. S3*D*). Overall, while there are some poorly supported loops in the structure model, the core of the RAM1–CBX1 interaction is well supported and is maintained in WRI5b–RAM1 interactions (*SI Appendix*, Figs. S7 and S8).

The model of the RAM1 and CBX1 complex suggested that the most critical interaction segment of CBX1 lies in the helix comprising residues 291 to 304, which encompass the M2/M2b motif (amino acid 293 to 302, Fig. 4*B* and *SI Appendix*, Figs. S4 and S5*B*), supporting the results of the Y2H assay (Fig. 3*B*). This region CBX1_r1_ (Fig. 4*H*) was predicted to interact with RAM1 residues Q312, L316, Q317, L318, V319, H320, L323, E327, M355, L576, L579, H580, S583, and D587 as they are all within 3 to 4 Å of CBX1_r1_. This group of residues in RAM1 is referred to as RAM1_r3_ (Fig. 4*H*).

In order to experimentally interrogate whether the CBX1 residues emerging from the model are important for the interaction, we generated a series of CBX1 mutants: We deleted the interacting CBX1_r1_ helix corresponding to residues 291 to 304 (CBX1m1, *SI Appendix*, Fig. S9*A*). We also separately deleted the two halves of the CBX1_r1_ helix to generate CBX1m2 and CBX1m3 (*SI Appendix*, Fig. S9*A*). In the CBX1_r2_ region, four leucine residues (L293, L295, L296, and L297), conserved across M2/M2b domains appear important in coordinating interaction with RAM1 in a sort of space-filling lock-and-key fit (Fig. 4*E*). We mutated these residues to glycine, creating CBX1m4 (*SI Appendix*, Fig. S9*A*).

The CBX1_r3_ region, contained two further sites of interest: One contains residue S299 and S300 which seem to coordinate a salt bridge or hydrogen bond with H320 of RAM1 as they are within 3 Å of one another, an optimal distance for hydrogen bond donors and acceptors (Fig. 4*F*). We interrogated the importance of this serine–histidine interaction by generating the mutant CBX1m5, with both serines substituted to alanine (*SI Appendix*, Fig. S9*A*). The second site is CBX1 residue F302 which is coordinating an extensive van der Waals network with RAM1 residues V319, L323, L576, L579, and H580 (RAM1_r2_) (Fig. 4*G*). Therefore, we introduced the mutation F302G, resulting in the mutant CBX1m7 (*SI Appendix*, Fig. S*9A*). As a negative control, we tested the function of the residue E304 by mutating it to alanine creating CBX1m6 (*SI Appendix*, Fig. S9*A*). E304 is part of the interacting helix, but is the last residue pointing away from the interaction interface with RAM1 (*SI Appendix*, Fig. S7*D*). Thus, while this residue and its backbone are within interacting distance to RAM1, the function of the carboxyl group on the glutamic acid is not predicted to be important, as, based on the predicted structure, it is too far away (6.9 Å) from the nearest h-bonding partner (*SI Appendix*, Fig. S7*E*).

The CBX1 mutants (m1-m7) were tested for interaction with RAM1 in Y2H assays and in transactivation assays using p*PT4* as the model promoter (*SI Appendix*, Fig. S9 *A* and *C*). The wild-type version of CBX1 strongly interacted with RAM1 in yeast. Some of the CBX1 mutant versions (m1, m3, m4, m7) showed slight autoactivity; however, none of them supported additional yeast growth in the presence of RAM1 (*SI Appendix*, Fig. S9*A*). The exception is CBX1 m6 confirming the prediction that E304 is not involved in the interaction (*SI Appendix*, Fig. S7 *D* and *E*).

In transactivation assays, the combination of wild-type CBX1 and RAM1 conferred strong *PT4* promoter activity as observed before (*SI Appendix*, Figs. S2*B* and S9*C*). All CBX1 mutant proteins except for m5 and m6 were unable to transactivate the promoter significantly together with RAM1, with m2 showing the strongest effect (*SI Appendix*, Fig. S9*C*). While m6 with RAM1 fully transactivated the *PT4* promoter, m5 with RAM1 showed a significant drop in activity. Also, in BiFC assays in *L. japonicus* hairy roots, the mutants CBX1 m4 or m7, used as examples, were unable to interact with RAM1 as no BiFC signal was observed, irrespective of arbuscule development stage (*SI Appendix*, Fig. S9*D*). Thus, the leucines (likely for stabilization of the interaction) and the phenylalanine in the M2/M2b motif are all required for the interaction with RAM1.

**Fig. 5.**
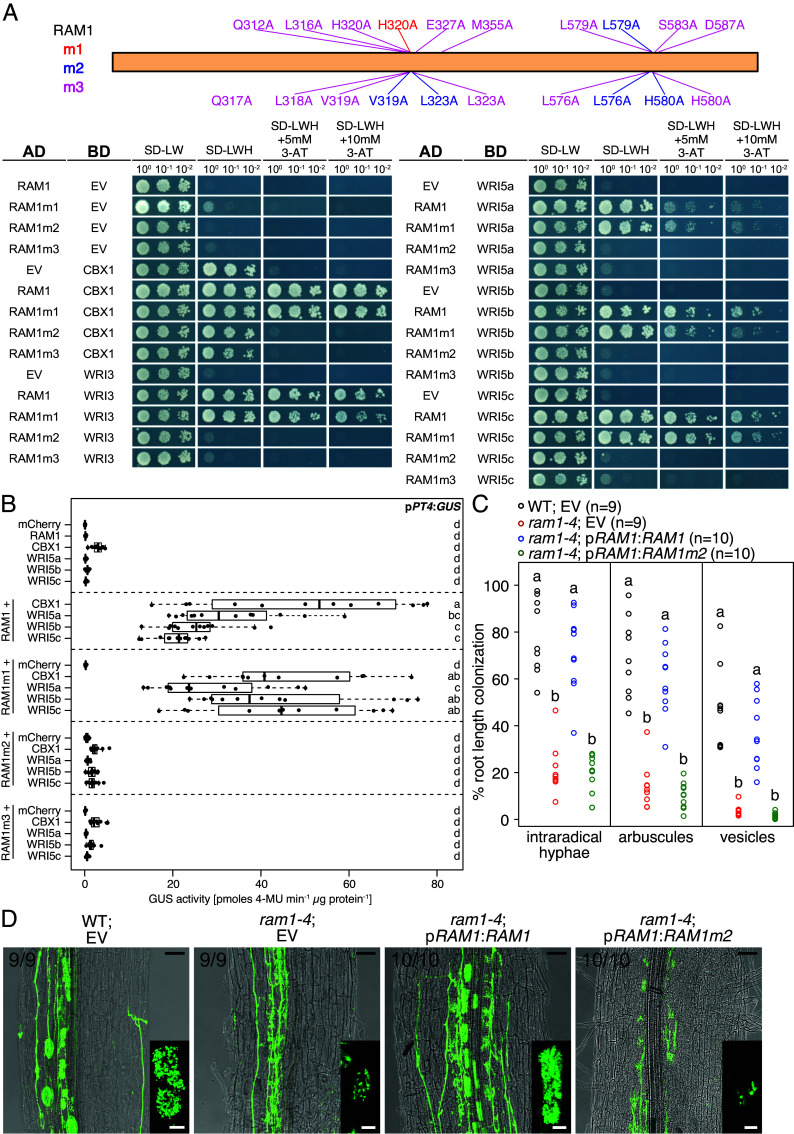
Mutation of RAM1 residues predicted to be involved in interaction with WRIs reduces interaction capability. (*A*) GAL4-based Y2H assay to test interaction of mutant versions of RAM1 (bait) and five AM-induced WRI transcription factors (prey). All coding-sequence-containing plasmids were used in combination with the complementary EV as negative controls. Expressed proteins, optical density of dropped yeast cultures, amino acids lacking from the medium and 3-AT concentration are provided in the figure. The schematic representation on top shows the positions of amino acid substitutions of the different mutant versions of RAM1. The experiment was performed three times with similar results. (*B*) Transactivation assay in *N. benthamiana* leaves showing activity of wild-type and mutated versions of RAM1 in combination with 5 AM-induced WRI proteins on the *PT4* promoter. The genomic sequence encoding the proteins indicated at the *y*-axis was driven by the *LjUbiquitin10* promoter. Bold black line, median; box, interquartile range; whiskers highest and lowest data point within 1.5 interquartile range; dots, actual values. Different letters indicate different statistical groups (ANOVA; post hoc Tukey; n = 12; *P* < 0.05). (*C*) Root length colonization parameters of wild-type and *ram1-4* hairy roots, colonized with *R. irregularis* at 6 wpi, transformed with the indicated expression cassettes. Different letters indicate different statistical groups (ANOVA; post hoc Tukey; *P* < 0.05). (*D*) Representative laser scanning confocal images of wild-type and *ram1-4* hairy roots colonized with *R. irregularis* at 6 wpi, transformed with the indicated expression cassettes. The numbers indicate the number of root systems that displayed the phenotype shown in the image, among the total number of analyzed root systems. Black scale bar, 50 µm. *Insets* show a close-up of arbuscules; white scale bar, 10 µm. The fungus is stained with WGA-Alexa-Fluor488. (*B*–*D*) The experiments were performed once.

To validate our predictions of RAM1 residues involved in the interaction with the WRI proteins, we generated mutant versions of RAM1. For the RAM1 mutant m1 H320 was substituted to alanine and for RAM1m2 residues V319, L323, L576, L579, and H580 were substituted with alanine to decrease the hydrophobicity and increase the distance between potentially interacting residues to outside of van der Waals distance (Fig. 4*G*). To create RAM1 mutant m3 all interacting residues of region r3 Q312, L316, Q317, L318, V319, H320, L323, E327, M355, L576, L579, H580, S583, and D587 (Fig. 4*B*) were changed to alanine. In Y2H assays the m1 mutant version of RAM1 displayed similar interaction with the WRI proteins than wild-type RAM1. The versions m2 and m3 were unable to interact with the WRI proteins in yeast (Fig. 5*A*). In transactivation assays, wild-type RAM1 and the m1 mutant activated p*PT4* in the presence of any of the four WRI proteins, while transactivation was abolished when the RAM1 m2 and m3 mutants were coexpressed with the WRIs, although the RAM1 m2 and m3 mutant proteins accumulated stably in *N. benthamiana* leaves (Fig. 5*B* and *SI Appendix*, Fig. S9*B*). Also BiFC assays did not detect interaction of RAM1m3 with CBX1 or WRI5b in colonized *L. japonicus* hairy roots (*SI Appendix*, Fig. S9*D*). Also, we transgenically complemented *ram1-4* hairy roots with wild-type *RAM1* and *RAM1m2* driven by the endogenous *RAM1* promoter (Fig. 5 *C* and *D*). Wild-type RAM1 restored arbuscule branching and full root length colonization, while complementation with *RAM1m2* did not change the *ram1* phenotype with stunted arbuscules and low root length colonization. In conclusion, the hydrophobic residues V319, L323, L576, L579, and H580, which are distributed across three alpha-helices in RAM1, are crucial for its interaction with the phenylalanine in the M2/M2b-domain of WRI proteins, the activation of target promoters, and RAM1 function in arbuscule branching.

### Ectopic Expression of *CBX1*, *WRI3,* and *WRI5b* Restores Arbuscule-Formation in *cyclops*.

We previously demonstrated that ectopic expression of *RAM1* driven by a *Ubiquitin* promoter restores arbuscule formation in the *cyclops* mutant, which usually blocks arbuscule formation in *L. japonicus* (14). This supported the conclusion that *RAM1* is activated by and acts downstream of CYCLOPS in AM development. If RAM1 functions in a complex with the WRI transcription factors it would be expected that their ectopic expression can also restore arbuscule formation in the *cyclops* background but not in the *ram1* background. To address this wild-type, *cyclops-4* and *ram1-3* hairy roots, transformed with *RAM1, CBX1* and *WRI3* and with *WRI5a* and *WRI5b* as examples for *WRI5* genes, all driven by the *LjUbiquitin10* promoter were exposed to *Rhizophagus irregularis* (*SI Appendix*, Figs. S10–S12). All transformed wild-type roots were fully colonized with highly branched arbuscules (*SI Appendix*, Fig. S10*A* and *SI Appendix*, Fig. S11 *A*, *Upper*, *SI Appendix*, Figs. S10*B* and S11*B*). *cyclops-4* mutants transformed with the control EV showed hardly any colonization and absence of arbuscules and vesicles (14) (*SI Appendix*, Figs. S10 *A* and *B* and S11 *A* and *B*). Ectopic expression of all *RAM1*, *CBX1*, *WRI3,* and *WRI5b* restored formation of fully branched arbuscules in the *cyclops* mutant consistent with the notion that they act downstream of CYCLOPS (*SI Appendix*, Fig. S10*A* and *SI Appendix*, Fig. S11 *A*, *Middle*), while interestingly, *WRI5a* was unable to do so, indicating that WRI5a is the least potent transcriptional activator among the examined WRIs proteins with respect to genes required for arbuscule branching. Only ectopic expression of *CBX1* improved root length colonization of the *cyclops* mutant to the level of the wild type, while all other transcription factors did so to a much lesser extent (*SI Appendix*, Figs. S10*B* and S11*B*). CBX1 may have more target genes or might be more potent than the other WRI proteins in inducing genes in the *cyclops* background that are required for arbuscule formation. *ram1* hairy roots transformed with EV contained stunted arbuscules and showed strongly reduced colonization, in particular a drastic reduction in vesicles as reported before (11–14) (*SI Appendix*, Figs. S10 *A* and *B* and S11 *A* and *B*). When *RAM1* was overexpressed in *ram1* it restored colonization and arbuscule branching to wild-type levels. Ectopic expression of neither of the *WRI* genes had any effect on arbuscule morphology and colonization level in the *ram1* mutant (*SI Appendix*, Fig. S10 *A* and *B* and *SI Appendix*, Fig. S11 *A*, *Lower* and *SI Appendix*, Fig. S11*B*), suggesting that the WRI proteins depend on RAM1 to promote arbuscule branching and/or that RAM1 targets more genes required for arbuscule branching than the WRI proteins.

We assessed whether ectopic expression of *CBX1* as an example for a *WRI* gene that can restore arbuscule branching and root length colonization, and *WRI3* as an example for a *WRI* gene that can only restore arbuscule branching in the *cyclops* background, can cause activation of *RAM2, STR, and PT4* in the absence of the fungus in *cyclops* and *ram1*. All ectopically expressed genes displayed increased transcript levels in all genetic backgrounds (*SI Appendix*, Fig. S12, *Upper*). As observed before, ectopic expression of *RAM1* caused increased transcript levels of *RAM2*, *STR,* and *PT4* in all genotypes (14) (*SI Appendix*, Fig. S12, *Lower*). Ectopically expressed *CBX1* increased transcript levels of *RAM2*, *STR,* and *PT4* in the wild-type but even more strongly in the *cyclops* mutant hinting toward transcriptional inhibition in the presence of CYCLOPS. In contrast, ectopically expressed WRI3 failed to induce the transcripts at the whole root system level in any genotypic background (*SI Appendix*, Fig. S12, *Lower*), consistent with WRI3’s weaker ability to restore AM development in the *cyclops* mutant as compared to CBX1 (*SI Appendix*, Fig. S10). Ectopic expression of neither *CBX1* nor *WRI3* caused significant increases of *RAM2, STR,* and *PT4* expression in the *ram1* mutant consistent with a requirement of RAM1 for their action. Together, the complementation experiment and the AM marker gene induction after ectopic expression of *RAM1*, CBX1 and *WRI3* in the absence of the fungus confirm that RAM1 acts together with WRI transcription factors to induce gene expression in AM.

### *CBX1* and *WRI3* Are Transcriptional Targets of CCaMK, CYCLOPS, and DELLA.

**Fig. 6.**
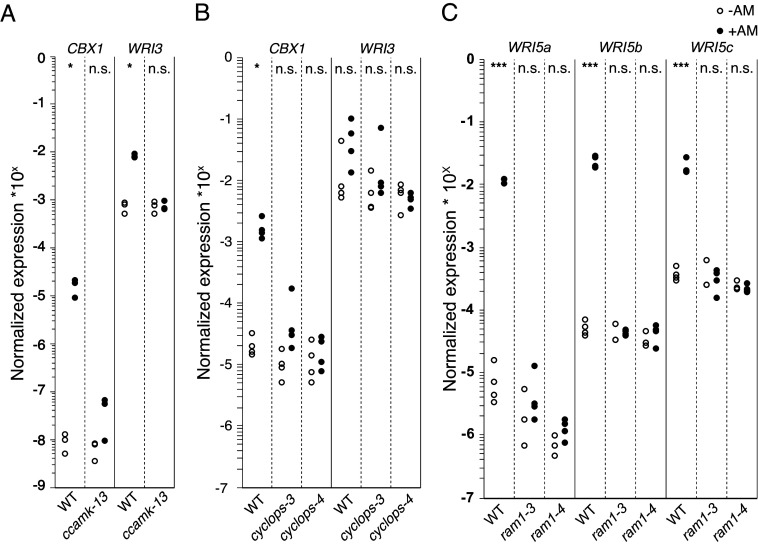
Expression of WRI encoding genes during AM symbiosis. (*A* and *B*) Transcript accumulation of *CBX1* and *WRI3* in roots of the indicated genotypes colonized by *R. irregularis* at 6 wpi. Transcript accumulation was determined by RT-qPCR. The housekeeping gene *Ubiquitin10* was used for normalization. Corresponding colonization data are displayed in *SI Appendix*, Fig. S13 *A* and *B*. (*C*) Transcript accumulation of *WRI5a*, *WRI5b,* and *WRI5c* in roots of the indicated genotypes colonized by *R. irregularis* at 5 wpi. Transcript accumulation was determined by RT-qPCR, and the housekeeping gene *Ubiquitin10* was used for normalization. Corresponding colonization data are displayed in *SI Appendix*, Fig. S14. Statistical analysis: Welch *t* test [n = 3 (*A*), n = 4 (*B* and *C*), #*P* < 0.1, *P < 0.05, **P < 0,01, ***P < 0.001]. All experiments were performed twice with similar results.

As RAM1 functions in a heterodimer with AM-induced WRI transcription factors, we hypothesized that *RAM1* and the *WRI* genes are coexpressed and coregulated by the same factors. Indeed, the promoters of *RAM1*, *CBX1*, *WRI5a*, *b,* and *c* are all active in arbuscule-containing cells (14, 30, 34). It was previously shown in *M. truncatula* that the induction of *CBX1* and *WRI3* during AM symbiosis is independent of *RAM1*, while the induction of *WRI5a*, *b,* and *c* is dependent on *RAM1* (30). Therefore, we hypothesized that *CBX1* and *WRI3* could be coregulated with *RAM1*, while *WRI5a*, *b,* and *c* could be part of a second transcriptional wave. The expression of both *CBX1* and *WRI3* is induced upon root colonization by *R. irregularis* with *WRI3* being more strongly expressed in the absence of AM than *CBX1* (Fig. 6 *A* and *B* and *SI Appendix*, Fig. S13 *A* and *B*), leading to expression differences between AM and control roots to be nonsignificant in some experiments (Fig. 6*B*). Similarly to *RAM1* (14), the induction in response to colonization by *R. irregularis* of both *CBX1* and *WRI3* depends on *CCaMK* and *CYCLOPS* (Fig. 6 *A* and *B* and *SI Appendix*, Fig. S13 *A* and *B*). Consistently, the expression of *WRI3* and *CBX1* is induced by overexpression of the CCaMK gain of function version CCaMK^314^ in hairy roots (*SI Appendix*, Fig. S13*C*), as shown for *RAM1* (14). However, unlike for *RAM1,* this induction occurred also in *cyclops* roots, suggesting an additional, CYCLOPS-independent factor activating their expression in the presence of autoactive CCaMK^314^.

To address directly whether *CBX1* and *WRI3* can be transcriptionally induced by CCaMK-activated CYCLOPS, we performed transactivation assays in *N. benthamiana* leaves using *GUS* fusions with sequence fragments 2070 and 1958 bp upstream of the start codon of *CBX1* and *WRI3* promoters, respectively. The promoters were strongly activated in the presence of CCaMK^314^ and CYCLOPS, while they were hardly active in the presence of the mCherry control (*SI Appendix*, Fig. S13 *D* and *E*). Examination of the promoter sequences of *CBX1* and *WRI3* revealed the core palindrome sequence (GCCGGC) of the *AMCYC*-RE, a CYCLOPS binding site that was previously identified in the *RAM1* promoter (14). Mutation of this motif abolished activation of the *CBX1* and *WRI3* promoters by CCaMK^314^ and CYCLOPS (*SI Appendix*, Fig. S13 *D* and *E*), confirming that *WRI3* and *CBX1* are, like *RAM1*, targets of CCaMK and CYCLOPS.

CYCLOPS and CCaMK form a complex with the GRAS protein DELLA1, the proteolytic target of GA signaling, to regulate *RAM1* (14, 53) RT-qPCR showed that, as for *RAM1* (14), ectopic expression of *DELLA1^Δ17^*, that is resistant against GA-mediated degradation (53), increases transcript levels of both *CBX1* and *WRI3* in hairy roots, in the absence of AM (*SI Appendix*, Fig. S13*F*). These results were recapitulated in nontransformed roots by pharmacological treatment with the GA biosynthesis inhibitor paclobutrazol (PAC), which leads to the stabilization of DELLA (*SI Appendix*, Fig. S13*G*). Furthermore, the induction of *CBX1* and *WRI3* expression by CCaMK^314^ was reduced to levels comparable to the EV control by the application of exogenous GA_3_ (*SI Appendix*, Fig. S13*C*). The induction of *CBX1* and *WRI3* during symbiosis and after PAC treatment was mostly independent of *RAM1* (*SI Appendix*, Figs. S13 *G* and *H* and S14*A*), confirming the results from *M. truncatula* that RAM1 is not required for their activation (30) and supporting that CBX1, WRI3, and RAM1 act at the same level in a regulatory cascade.

### *WRI5a*, *b,* and *c* Promoters Can Be Transactivated by CBX1 and RAM1.

We examined whether the AM-mediated induction of *WRI5a*, *WRI5b,* and *WRI5c* upon AM depends on *RAM1* like in *M. truncatula* (30). While all three genes were induced by AM in wild type, no increase in their transcript levels was detected in colonized roots of two allelic *ram1* mutants (Fig. 6*C* and *SI Appendix*, Fig. S14*A*). Consistent with CCaMK and CYCLOPS acting upstream of *RAM1*, *WRI5a*, *WRI5b,* and *WRI5c* were also not induced in the *ccamk* and *cyclops* roots during root colonization (*SI Appendix*, Fig. S14 *B* and *C*). Furthermore, ectopically expressed *DELLA1^Δ17^*was unable to induce *WRI5a* and *b* in the absence of the fungus. *WRI5c* was induced only to a small extent and the difference was not significantly different from the control (*SI Appendix*, Fig. S14*D*).

Since RAM1 activates the *RAM2*, *STR,* and *PT4* promoters through interaction with the WRI transcription factors, we hypothesized that the *WRI5a*, *WRI5b,* and *WRI5c* promoters would be similarly activated by RAM1–WRI complexes. Thus, we performed *N. benthamiana* leaf-based transactivation assays with promoter fragments of 2,114 bp (*WRI5a*), 2,100 bp (*WRI5b*), and 2,035 bp (*WRI5c*) upstream of ATG. CBX1 or WRI3 alone were unable to induce any of the three promoters, while together with RAM1 they did activate all three promoters (*SI Appendix*, Fig. S14 *E*, *Left*). Surprisingly, RAM1 activated the *WRI5a* and *b* promoters even in the absence of CBX1 and WRI3 (*SI Appendix*, Fig. S14 *E*, *Right*). This activation was independent of the *AW*-boxes and *MYCS*-elements, in these promoters (*SI Appendix*, Fig. S14 *E*, *Upper* schematic). Therefore, we used the *WRI5b* promoter as a model to identify potentially unknown *cis*-elements and performed transactivation assays with a 5′ promoter deletion series. This revealed a region, responsive to CBX1 and RAM1, between the bases -187 and -161 upstream of ATG (*SI Appendix*, Fig. S15 *A* and *B*). Examination of this DNA stretch revealed the presence of the motif CCTTGT, which despite a T to C replacement in the first position and the loss of a second T at the 3′ end is identical with the core sequence of the *MYCS*-element (TTCTTGTT) (54). Therefore, we termed this motif *coreMYCS* (*cMYCS*). Mutation of *cMYCS* in the wild-type *WRI5b* promoter caused a strong drop in RAM1 and CBX1-mediated transactivation (*SI Appendix*, Fig. S15*C*), indicating that *cMYCS* alone explains induction of the *WRI5b* promoter by CBX1 and RAM1. As *cMYCS* is also located very close to the transcriptional start site of the *WRI5a* and *WRI5c* promoters, this observation can likely be extrapolated to all *WRI5* promoters. The wild-type version of the *WRI5b* promoter driving a *GUS* reporter gene was inducible by colonization of transgenic wild-type hairy roots, while mutation of the *cMYCS* strongly reduced the blue staining, despite colonization (*SI Appendix*, Fig. S15*D*). This suggests that in *L. japonicus* roots RAM1 majorly acts in concert with CBX1 and WRI3 (or other AP2 transcription factors that can bind *cMYCS*) to activate the employed promoter fragment of *WRI5b*, while an interaction with a DNA-binding transcription factor that binds another *cis*-element is less likely in the native context. Furthermore, RAM1 is essential for transcriptional activation via the *cMYCS* element during colonization, as no blue staining was observed upon root colonization when p*WRI5b* controlled GUS expression in the *ram1* mutant background (*SI Appendix*, Fig. S15*D*). The isolated *cMYCS*-element fused in duplicate and quadruplicate tandems and linked to a *35S* minimal promoter was sufficient for activation by RAM1 and CBX1 (*SI Appendix*, Fig. S15*C*). Binding of CBX1 to the *cMYCS*-element was also confirmed by EMSA (*SI Appendix*, Fig. S15*E*). Thus, *cMYCS* appears to be an additional target site for the RAM1–CBX1 complex. This notion is further supported by the conservation of the *cMYCS* in the promoter sequences of *M. truncatula* p*WRI5b* and *c* (*SI Appendix*, Fig. S15*F*).

**Fig. 7.**
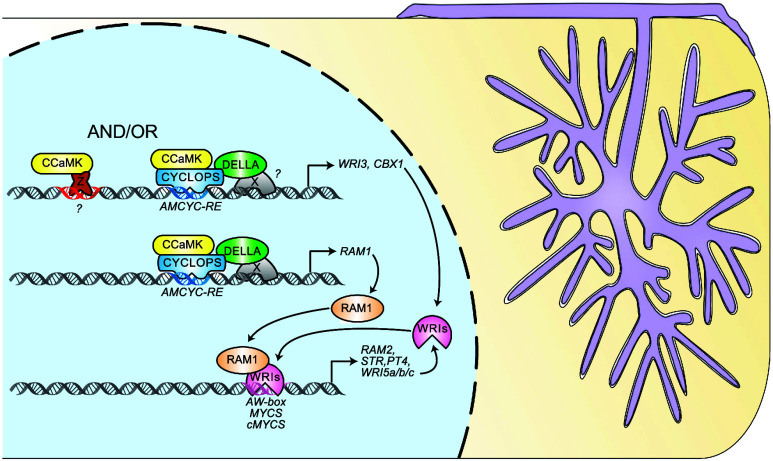
Model of gene regulation by RAM1 and WRI transcription factors during AM. The expression of *RAM1, CBX1* and *WRI3* is activated by the CCaMK–CYCLOPS–DELLA complex via the *AM-CYCRE* in their promoters. DELLA can also activate the transcription of the three genes in the absence of CYCLOPS, suggesting an additional, so far unknown, DNA-binding transcription factor X (14). The transcription of *CBX1* and *WRI3* can also be induced by ectopic function of dominant active CCaMK^314^ in the absence of CYCLOPS, suggesting that their promoters can be targeted by (a) currently unknown CCaMK phosphorylation target(s) Z. Subsequently the GRAS protein RAM1 interacts with the WRI transcription factors CBX1 and WRI3 and can induce transcription of *RAM2*, *STR,* and *PT4* to enable lipid for phosphate exchange between plant and fungus. Likely, also the WRI transcription factor encoding genes *WRI5a*, *b,* and *c* are transcriptional targets of CBX1, WRI3, and RAM1. WRI5a, b, and c also interact with RAM1 and activate *RAM2*, *STR,* and *PT4* promoters and potentially their own promoters.

We propose that the five AM induced WRI genes act in two waves in which *CBX1* and *WRI3* are activated by the CCaMK–CYCLOPS–DELLA complex (and an alternative transcription factor complex activated by CCaMK) and *WRI5a*, *b,* and *c* are activated by the RAM1–CBX1 (and likely RAM1–WRI3) complex through the *cMYCS*-element (Fig. 7).

## Discussion

A number of GRAS transcription factors involved in regulating AM symbiosis have been discovered in the past decade (9, 55–57). It remained unknown how they interact with their target promoters. We addressed this knowledge gap for RAM1, a central regulator of genes required for arbuscule branching and symbiotic nutrient exchange. We show that RAM1 interacts with five AM-induced transcription factors of the WRI family (CBX1, WRI3, WRI5a, b, and c) to activate promoters of key genes, necessary for AM development and function, namely *RAM2, STR,* and *PT4* (58, 59).

Our conclusion rests on six orthogonal pieces of evidence: 1) RAM1 can significantly transactivate *RAM2*, *STR,* and *PT4* promoters only in the presence of CBX1, WRI3, or WRI5a, b, or c and vice versa. 2) This transactivation depends on WRI binding-sites within the target promoters. 3) RAM1 can induce target promoters in hairy roots only in the presence of WRI binding sites. 4) RAM1 directly interacts with all five WRI proteins in yeast (Y2H assay) *N. benthamiana* leaves (Co-IP) and *L. japonicus* roots (BiFC) (Fig. 2 *B* and *C*). 5) Computational protein modeling predicted the interactions and suggested the amino acids of RAM1 and the WRI proteins that could then be experimentally confirmed to be involved in the interaction. 6) The WRI proteins interact with RAM1 via amino acids in the M2/M2b motif, which is conserved across all five WRI proteins, and underrepresented in WRI proteins of plant species that have secondarily lost AM. These findings are further supported by another study in which chromatin immunoprecipitation with RAM1 from *M. truncatula* hairy roots pulled down the *RAM2* promoter (10). Since cross-linking was applied in this experiment, it is likely that the full protein complex that mediates DNA association of RAM1 and contains AM-induced WRI proteins was pulled down.

CBX1 and WRI5a have previously been shown to bind to the DNA via *MYCS*-elements and *AW*-boxes (34, 35). We provide evidence that this should also apply to WRI3, WRI5b, and WRI5c, as mutation of these elements abolishes the ability of RAM1 and any of these three WRI proteins to activate target promoters. Furthermore, the DNA-binding AP2 domains (60) of all five WRI proteins are highly conserved, suggesting their affinity to the same *cis*-elements. As the WRI proteins are unable to activate promoters in the absence of RAM1, we propose that they provide the DNA-binding domain, while RAM1 contributes the activation domain required for activating transcription. Indeed, in contrast to RAM1 (Fig. 2) none of the WRIs shows autoactivation in a Y2H assay when bound to the GAL4-binding domain except for CBX1, which also weakly transactivates promoters in *N. benthamiana* leaves and *Arabidopsis* cell culture (34). Thus, CBX1 may have a domain that can act as an activation domain. Interestingly, CBX1 is also, upon ectopic expression, more potent than RAM1, WRI3, WRI5a, and b in restoring AM in the *cyclops* background. The reason for this is currently unknown. Possibly it can interact with additional transcription factors and/or somehow boost *RAM1* expression in the absence of CYCLOPS (14, 35).

Several studies highlighted the importance of the GRAS domains for heteromerization of GRAS factors with other proteins involved in transcriptional regulation (21, 27, 28). We found that in RAM1 the GRAS domain is required for interaction with the five WRI proteins. The M2/M2b domain is underrepresented in WRI proteins from AM-nonhost species, supporting its role in interacting with AM-specific transcriptional regulators such as RAM1. The question remains, whether the few remaining *WRI* genes encoding M2/M2b motifs in AM-nonhost genomes underwent neofunctionalization or if they represent genomic remnants from secondary loss of AM symbiosis. The single AM-relevant WRI protein of the liverwort *M. paleacea* also contains the M2/M2b domain, while the currently available *M. paleacea* genome appears to lack a *RAM1* gene (42). It is possible that in *M. paleacea*, RAM1 is replaced by another transcriptional coregulator or that MpWRI contains an activation domain, making it independent of coactivators.

Interactions between GRAS proteins and AP2/ERF transcription factors not containing an M2/M2b domain have been reported in other contexts. The *Arabidopsis* DELLA protein GAI interacts with RAP2.3, thereby limiting its transactivation potential during seedling development (61). Furthermore, a large-scale Y2H screen identified numerous AP2 transcription factors interacting with DELLA; however, these interactions were functionally not further characterized (62). Other reports showed ERF5,114 and 115 interacting with GRAS proteins of the PAT1 branch (SCL1,5,13,21), to regulate chitin-induced innate immune response and tissue regeneration in response to wounding (63, 64). Thus, there is likely a diversity of interaction surfaces between GRAS proteins and AP2 transcription factors.

We provide evidence on how the *WRI* genes may be regulated. Similar to *RAM1* (14), *CBX1* and *WRI3* can be activated by CYCLOPS together with autoactive CCaMK^314^ via the palindromic *AMCYC*-RE present in their promoters, as well as by stabilized DELLA, suggesting that *RAM1*, *CBX1* and *WRI3* are coregulated by the same CCaMK–CYCLOPS–DELLA complex.

The promoters of *WRI5a*, *WRI5b,* and *WRI5c* do not contain *AMCYC*-REs. Instead, they contain *MYCS*-elements and *AW*-boxes and can be activated by CBX1 or WRI3 together with RAM1, consistent with the dependence of their AM-mediated activation on *RAM1* (30) and their induction by *CBX1* overexpression (34). We identified an additional *cis*-element in their promoter that we named *cMYCS* because it resembles the core sequence of the *MYCS*-element (CTTGTT), although it comprises an additional C at the 5′ end and misses a T at the 3′ end. This element is conserved in all three promoters (and in *M. truncatula* p*WRI5b* and *c*), located closely to the start codon and was sufficient for being transactivated by CBX1 and RAM1. It was shown recently that in *M. truncatula* induction of *WRI5a* and *c* is dependent on a periarbuscular membrane-localized *CYCLIN-DEPENDENT KINASE 2* (*CDK2*) gene (65). It will be interesting to understand in the future whether this wiring is conserved in *L. japonicus* and whether CDK2 triggers a signal transduction cascade that affects the activity of *RAM1*, *CBX1* and/or *WRI3* or whether it affects alternative transcription factors, which (co-)regulate expression of *WRI5a* and *c*, but not *WRI5b*.

Based on our data, we propose that two waves of gene induction are responsible for the presence of WRI transcription factors for transcriptional regulation in arbuscule-containing cells. The first wave comprises *CBX1* and *WRI3* that together with *RAM1* are directly induced by CCaMK-activated CYCLOPS and DELLA, while the second wave includes *WRI5a*, *b,* and *c*, which are activated by the WRIs of the first wave together with RAM1 (Fig. 7). This two-step mode of transcriptional activation of two and three WRI transcription factors may lead to an amplification of the transcriptional signal by enlarging the pool of redundant WRI factors or it may provide arbuscule developmental stage specificity and/or flexibility to the system through differences in promoter affinity among the WRI transcription factors. CBX1, WRI3, and the WRI5a, b, and c belong to different subfamilies of the WRI proteins, and although the DNA-binding AP2 domains of all five are well conserved, the N and C termini are less conserved and differ particularly among the WRIs of the first and the second transcriptional waves. The N and C termini could interact with yet additional transcriptional regulators involved in AM-induced promoter activation, depending on their amino acid composition. Thus, individual WRIs may preferentially activate a subset of promoters, depending on their interactors, the composition of, and the distance among *cis*-elements in the individual promoters. In fact, a larger cohort of AM-induced promoters than examined here contains *MYCS*-elements and *AW*-boxes (34) and they could potentially belong to subgroups with different *cis*-element compositions. It can therefore be assumed that these are all regulated by RAM1–WRI complexes possibly in a spatiotemporally complex manner. In semireductionist transactivation assays in *N. benthamiana* leaves, we already reproducibly observed differences in the potency of different WRI transcription factors to activate certain promoters. It will be interesting to determine whether and how the distribution and relative abundance of *cis*-elements within regulatory sequences contributes to the fine-tuning of spatiotemporal gene expression patterns and binding of different WRI proteins in the context of AM. Identification of further interacting transcriptional regulators, the effects of their combinatorial fluctuations with members of the WRI transcription factor pool, as well as analysis of plant mutants in all five *WRI* genes is required to understand to which extent these WRIs act redundantly, or whether the amplification of AM-induced *WRI* genes in vascular plants [as compared to e. g. *M. paleacea* (42)] has increased the complexity and specificity of transcriptional regulation in root cortex cells during arbuscule development.

## Materials and Methods

Details of plant materials and growth conditions, yeast-two-hybrid, Co-IP, BiFC, microscopy, and quantification of root colonization by AM fungi, qRT-PCR analysis, plasmid generation, plant transformation, promoter-reporter analysis, transactivation assays, protein purification, EMSA, phylogenetic and bioinformatic analyses, and protein structure modeling are provided in *SI Appendix*.

Constructs and primers are listed in *SI Appendix*, Tables S2–S5; benchmark data of the dataset submitted to phylogenetic analysis are listed in *SI Appendix*, Table S6.

## Supplementary Material

Appendix 01 (PDF)

## Data Availability

All study data are included in the article and/or *SI Appendix*.
