# Supplementary material for: The GRAS protein RAM1 interacts with WRI transcription factors to regulate plant genes required for arbuscule development and function
Source: Proc Natl Acad Sci U S A. 2025 May 19;122(21):e2427021122. doi: 10.1073/pnas.2427021122 (PMC12130850; doi:10.1073/pnas.2427021122)
Supplement: Supplementary file 1 — Appendix 01 (PDF) [file pnas.2427021122.sapp.pdf]

Supporting Information for

The GRAS protein RAM1 interacts with WRI transcription factors to regulate plant genes required for arbuscule development and function

Michael Paries, Karen Hobecker, Sofia Hernandez Luelmo, Filippo Binci, Angelica Guercio, Annika Usländer, Catarina Cardoso, Yang Si, Lotta Wankner, Sagar Bashyal, Philip Troycke, Franziska Brückner, Priya Pimprikar, Nitzan Shabek, Caroline Gutjahr

Corresponding author: Caroline Gutjahr
Email: gutjahr@mpimp-golm.mpg.de

This PDF file includes:

Supplementary Material and Methods

Figures S1 to S15

Tables S1 to S6

Supplementary Materials and Methods

Plant material, growth conditions and inoculation with AM fungi

For all experiments *L. japonicus* ecotypes Gifu and MG20 wild type and mutants were used. The mutants *ccamk-3*, *ccamk-13*, *cyclops-3*, *cyclops-4*, *ram1-3*, *ram1-4*, *ram2-1*, *str* have been described previously (1–5).

Seeds were scarified with sand paper and surface sterilized with 0,1% SDS, 0,3% NaClO. Imbibed seeds were germinated on 0,8% Plant Agar (Duchefa) at 24°C constant temperature, 60% air humidity and 16-h-light/8-h-dark cycles for 10-14 days. Plantlets were then transferred to pots (6-9 per pot) containing washed and autoclaved quartz sand (Casafina, grain size 0,7-1,2mm) and grown at 20°C constant temperature, 50% air humidity and 16-h-light/8-h-dark cycles. Immediately before planting and once per week each pot was fertilized with 30-40ml of modified half strength Hoagland solution containing 20µM phosphate (6). Twice per week the pots were watered with 30-40ml of autoclaved, de-ionized tap water. For arbuscular mycorrhiza colonization pots were inoculated with 350 spores of *R. irregularis* DAOM197198 (Agronutrition, Toulouse, France) per plant. Plants were treated by GA_3 (Sigma, G7645) or PAC (Fluka, 46046) by watering/fertilizing with the above-mentioned solutions containing the respective chemical in the final concentration indicated in the respective figure legend. Control plants received equal amounts of the solvent ethanol. Treatment started one week post planting (1).

Yeast-two-hybrid

Coding sequences of *RAM1*, *DELLA1*, *CBX1*, *WRI3.1*, *WRI3.2*, *WRI5a*,*b*,*c* and truncated/mutated versions thereof were cloned into Gateway modified yeast vectors pGAD424 (Clontech) or pBD-GAL4 Cam (Stratagene). Constructs are listed in Supplementary Table 5. Transformation of the yeast reporter strain HF7c and drop test were performed according to standard protocols (Clontech Laboratories, Inc. Yeast Protocols Handbook). For the transformation, liquid YPAD medium was inoculated with a single yeast colony not older than 4 weeks and grown overnight at 28°C at 200rpm horizontal shaking. From this culture fresh YPAD medium was inoculated to a final OD_{546} of 0,2 and grown at 28°C at 200rpm until an OD_{546} between 0,6 and 0,9. Cultures were centrifuged at 800g at RT for 5min and the pellet was resuspended in 1ml sterile ddH_2O. For one transformation reaction, 100µl of the resuspended cells were mixed with 240µl 50% PEG 3350, 36µl 1M LiAc, 25µl denatured ss-DNA (2mg/ml) and 150g bait and prey plasmid respectively by vortexing for 50 seconds. After a 45 min incubation in a 42 °C water bath, the cells were centrifuged again at 800g for 5min. The pellets were then resuspended in 100µl 0,9% NaCl and streaked on SD-LW plates. The plates were incubated at 28 °C for three days.

For the drop-out assay, 2ml SD-LW liquid medium were inoculated with a single transformed colony and grown overnight at 28°C at 200rpm horizontal shaking. Cultures were centrifuged for 10min at

800g and washed with 1ml sterile 0,9% NaCl. The pellets were resuspended in 200µl 0,9% NaCl and the OD_{546} was measured and adjusted to 0.6, 0.06 and 0.006 (corresponding to dilutions 10^0, 10^{-1} and 10^{-2}) and 4µl of each dilution were applied on SD Medium plates (SD-LW, SD-LWH, SD-LWH + XmM 3-AT). The plates were sealed and incubated at 28°C for 2-3 days, before scanning.

Microscopy, BiFC and quantification of root colonization

R. irregularis structures in *L. japonicus* roots were stained with acetic acid and ink (7, 8). Roots were incubated for 15min at 95°C in 10% KOH. KOH was removed and roots were washed with tap water and with 10% acetic acid. Roots were covered with 5% black ink (Pelikan) and 5% acetic acid and incubated at 95°C for 5min. Ink and acetic acid were removed and roots were washed with tap water. For destaining, 5% acetic acid was added, followed by 20min incubation at room temperature and subsequent long-term storage at 4°C.

Root length colonization was quantified using a modified gridline intersect method (8, 9) and 10X magnification with a light microscope (Olympus CX23). Stained roots were cut into 1 cm long pieces and 20 root pieces were randomly selected. 10 arbitrary sections of every root piece were checked for fungal structures.

For confocal laser scanning microscopy, a LEICA TCS SP8 X WLL2 UPRIGHT HYVOLUTION 2 or an Olympus FV1000 CLSM Inverse were used. For BiFC imaging (Supplementary Table 1), a Stellaris 8 Confocal microscope (Leica) was used to image hairy roots transformed with plasmids for BiFC (pSHL_3-32 – pSHL_3-39) shown in Supplementary Table 5.

For fluorescence/brightfield microscopy imaging a DM6 B widefield microscope (Leica) was used. For confocal laser scanning microscopy or fluorescence microscopy fungal structures were stained with WGA Alexa Fluor 488 (8, 10) (www.lifetechnologies.com). Roots were incubated for 15min at 95°C in 10% KOH. KOH was removed and roots were washed with tap water. Roots were covered with 0,1M HCl and incubated for 2-3h. HCl was removed and the roots were washed with tap water three times and once with 1x PBS (pH 7.5). Roots were covered in 1x PBS with 0.2 µg/ml WGA AlexaFluor488 and placed in the dark at 4°C overnight and for long-term storage.

Gene expression analysis by RT-qPCR

Plant tissue was rapidly shock frozen in liquid nitrogen and stored at -80°C. RNA was extracted from tissue, ground in liquid nitrogen, with the Spectrum Plant Total RNA Kit (www.sigmaaldrich.com) and treated with DNaseI amplification grade (www.invitrogen.com). RNA purity was assessed with PCR. The BIORAD iScript cDNA synthesis kit (www.bio-rad.com) was used for cDNA synthesis from 800ng or 1µg of RNA. Primers were designed with Primer3 (11) and are shown in Supplementary Table 2. RT-qPCR was conducted with Jena Bioscience qPCR GreenMaster highROX (www.jenabioscience.com) and run in a BIORAD CFX 384 Real-Time PCR System using the following settings: 50°C 2min, 95°C 3min, 45 cycles of 95°C 10s, 55°C 20s, 72°C

30s followed by a primer dissociation curve analysis. Expression levels were calculated according to the ΔΔCt method (12). For each genotype or treatment three to four biological replicates were examined with two technical replicates.

Plasmid generation

Genes and promoter regions were amplified using Phusion or Q5 polymerase (NEB) according to standard protocols and using primers indicated in Supplementary Table 4. Plasmids were constructed by Gateway cloning using BB4 (13) as entry vector and Gateway LR clonase II Enzyme mix (Invitrogen) for recombination into the destination vector. For plant transformation plasmids were constructed by Golden Gate cloning (13) as indicated in Supplementary Table 5.

Plant transformation

Induction of transgenic *L. japonicus* hairy roots was performed using transgenic *A. rhizogenes* strain AR1193 as described previously (14). Liquid cultures of *A. rhizogenes* AR1193 were grown for 48h at 28 °C with 200 rpm horizontal shaking. Bacteria were pelleted and resuspended in 1 ml of ddH_2O. 100 µl of the bacterial suspension were plated on LB agar plates containing the appropriate antibiotics and incubated for 24h at 28°C. The bacterial lawn was resuspended in 3 ml sterile ddH_2O and the suspension was dropped on a piece of autoclaved filter paper. Two-weeks old *L japonicus* seedlings were arranged on the filter paper with their roots in the suspension. Shoots were cleaved off with a scalpel blade, within the suspension of *A. rhizogenes*. Subsequently, shoots were transferred to B5 agar plates without sucrose, sealed and incubated in the dark for 3 days. Then, the Petri dishes with plantlets were incubated for 5 days in the light. Subsequently, seedlings were transferred onto fresh B5 plates with sucrose and 300µg/ml cefotaxime. After additional 7 days, the plants were transferred to a fresh Petri dish. Fourteen days later, plants with transformed hairy roots were transplanted to pots. During harvest, transformed roots were identified with a stereomicroscope (Zeiss SteREO Discovery.V8) according to the presence of an mCherry fluorescent transformation marker.

N. benthamiana leaves were transiently transformed by infiltration of transgenic *A. tumefaciens* strain AGL1 as described (2). AGL1 cultures were grown in LB medium for 48h at 28°C and 200rpm horizontal shaking. The pellet was resuspended in infiltration buffer (10mM MES pH 5,6, 10mM $MgCl_2$, 150µM acetosyringone) and the OD_{600} value of the suspension was measured. Desired combinations were mixed to a final OD_{600} of 0,16 for each transformand carrying a different construct. The leaves of 2-3 week old *N. benthamiana* plant were infiltrated with the bacterial suspensions using a syringe.

Promoter-reporter analysis and transactivation assay

L. japonicus hairy roots transformed with plasmids containing p*RAM2*:*GUS* and p*STR*:*GUS*

expression cassettes were subjected to β-glucuronidase (GUS) staining as described (1). Roots were submerged in staining solution (0,1% Triton X-100, 1mM Na ferrocyanide, 1mM Na ferricyanide, 10mM EDTA pH 7, 100mM $NaPO_4$ pH 7, 1mM X-Gluc) and vacuum infiltrated in an excicator for five times. Infiltrated roots were incubated at 37 °C until a blue colour was observed. Staining solution was replaced with 70 % ethanol and roots were incubated overnight at 4°C before imaging and scanning.

For transactivation assays, *N. benthamiana* leaf discs (1 cm diameter) were punched out with a cork borer 2-3 days after infiltration, rapidly shock frozen in liquid nitrogen and stored at -80°C. After grinding in liquid nitrogen, the powder from one disc was resuspended in 100µl protein extraction buffer (50mM $NaPO_4$ pH 7, 10mM EDTA, 0,1% N-laurylsarcosine, 0,1% Triton X-100, 10mM 2-mercaptoethanol, 1x cOmplete™, Mini, EDTA-free Protease-Inhibitor-Cocktail) on ice. The supernatant was transferred to ice after 15min centrifugation at 15000rpm. 10µl of the supernatant were transferred to 100µl assay buffer (same as extraction buffer, w/o cOmplete™, with 1mM MUG) at 37°C and mixed. 10µl of the reaction were removed at distinct timepoints and terminated in 0,2 M Na_2CO_3. 4-MU fluorescence of the terminated reaction was measured for each timepoint and used to calculate pmoles 4-MU per minute for each sample. For normalization, this value was correlated with the protein content of the supernatant of each sample (determined in a Bradford assay), in order to determine the GUS activity in pmoles of 4-MU formed per minute, per µg protein.

Co-immunoprecipitation

Leaves of transiently transformed *N benthamiana* plants were harvested 3 days after *A. tumefaciens* infiltration and ground in liquid nitrogen, using a mortar. From the collected material, 400ml of the leaf powder were homogenized in 700ml of lysis buffer (50mM Tris-Cl, pH 8, 150mM NaCl, 1mM EDTA, 3mM DTT, 0.5% [v/v] Triton X-100, 1x complete mini-protease inhibitor cocktail (Roche) and 1mM PMSF). After three times of centrifugation at 14000rpm for 15min, the supernatants were incubated with 25µl of either GFP-Trap or Myc-Trap Magnetic Agarose beads for 20min at 4°C and then washed four times with wash buffer (50mM Tris-Cl, pH 8, 150mM NaCl, 1mM EDTA, 3mM DTT, 0,1% [v/v] Triton X-100, 1x complete mini-protease inhibitor cocktail [Roche], and 1mM PMSF). The beads were eluted with 50µl of SDS loading buffer (100mM Tris-Cl, pH 6,8, 4% [w/v] SDS, 0,2% [w/v] bromophenol blue, 20% [v/v] glycerol, and 200mM DTT). Then 20µl of each elution were analyzed by Western immunoblotting.

Protein purification and Electrophoretic Mobility Shift Assay

The open reading frame of the *CBX1* gene from *L. japonicus* was cloned into the expression vector pGEX-6P-1using the primers PGEX-CBX1-F and PGEX -CBX1-R (Supplementary Table 4), and then transferred into *E. coli* Rosetta (DE3). The recombinant strain was cultured in 1l LB medium that was supplemented with 100µg/ml carbenicillin at 37°C. Protein expression was induced at

OD_{600} ≈ 0,8 by the addition of isopropyl β-D-1-thiogalacto-pyranoside (IPTG) with a final concentration of 0,5mM. The induced cultures were then grown at 16°C for 12h. The GST-tagged recombinant protein was purified using Glutathione-Agarose (Thermo Scientific Pierce) under lysis buffer conditions (50mM Tris-HCl, 150mM NaCl, 1mM EDTA, 1mM DTT, pH 7,5). After binding to the resin, the column was washed thoroughly with lysis buffer to remove non-specifically bound proteins. On-column cleavage of the GST tag was performed by adding PreScission Protease in cleavage buffer (50mM Tris-HCl, 150mM NaCl, mM EDTA, 1 mM DTT, pH 7,5), followed by incubation at 4°C for 12h. The cleaved protein was collected in the flow-through, and the GST tag was retained on the column. The purified protein was subjected to electrophoretic mobility shift assay (EMSA) to identify CBX1 protein binding to Cy5 labeled cMYCS containing promoter fragments (sequences are listed Supplementary Table 3). For Cy5 labeled DNA probes, oligonucleotides were synthesized and labeled with Cy5 at their 5' end (Sigma-Aldrich) and annealed at 95°C for 10min. EMSA was performed as described before (4), the 20µL reaction mix (10mM Tris-HCl, pH 7,5, 0,25mM DTT, 2µg polydI/dC, 10% glycerol, 5mM $MgCl_2$, 10mM KCl, 500nM dsDNA probe and 500ng recombinant protein) was incubated on ice for 30min followed by loading on a 4% polyacrylamide gel. For competition, oligonucleotides (Supplementary Table 3) were synthesized and annealed as described above. Subsequently 10 to 50-fold more competitor than Cy5-labeled probe was added to the reaction prior to incubation.

In silico protein structure modelling

Full-length complex structures for CBX1-RAM1 and WRI5b-RAM1 were generated in RoseTTafold (15) through ColabFold (16) Notebook. The best model after 6 recycles was used for further analyses. Quality metrics of models are based on predicted Local Distance Difference Test (pLDDT) and Predicted Aligned Error (PAE). Structural illustrations, analyses, and measurements were generated in PyMol (17).

Phylogenetic and MEME analysis

The annotations of *L. japonicus CBX1*, *WRI3*, *WRI5a*, *WRI5b* and *WRI5c* were adopted from (4). The corresponding amino acid sequences were obtained from Lotus Base (18) (https://lotus.au.dk), additional *L. japonicus* sequences were found by protein BLAST of the 5 sequences against the Gifu v1.2 genome on Lotus Base (19). WRI amino acid sequences of *A. thaliana* were obtained by protein BLAST of the 5 *L. japonicus* WRI sequences on TAIR (20) (https://www.arabidopsis.org). WRI amino acid sequences from other species were obtained by protein BLAST of the 5 *L. japonicus* WRI sequences on PlantRegMap/PlantTFDB v5.0 (21) (http://planttfdb.gao-lab.org), NCBI (https://blast.ncbi.nlm.nih.gov/Blast.cgi) or EnsemblPlants (22) (https://plants.ensembl.org/index.html) (Supplementary Table 6). All sequences with a BLAST score of 150 or higher were used for the alignment, redundant sequences were excluded, as well

as incomplete sequences, if they were not repairable (repaired sequences are marked with _ in the end). Sequences without a unique species-specific identifier were amended with one (i.e. *Cephalotus follicularis* = XXXXX_Cepfo). Alignment was performed with the MUSCLE algorithm (23) in seaview5 (24). Phylogenetic reconstruction was carried out by first assessing the best substitution model using SMS (25) and then using PhyML (26) (www.atgc-montpellier.fr/phyml/) to compute maximum likelihood trees using the VT +G+F substitution model (27), with branch support using the aLRT SH-like method (28). The tree was plotted with FigTree v1.4.3 (tree.bio.ed.ac.uk/software/figtree/) and the branches were transformed into a cladogram.
To generate the position-frequency plot of the M2/M2b motif the MEME (29) tool from MEME Suite (30) (https://meme-suite.org/meme/) was used. The *cMYCS*-element was identified with the MEME (29) and tomtom (31) tools from MEME Suite (30).

Statistical Analysis

Statistical analysis was performed with R-Studio 2022.02.0 Build 443 and R version 4.1.3 (32, 33) (http://www.rproject.org)

Supplementary Figures

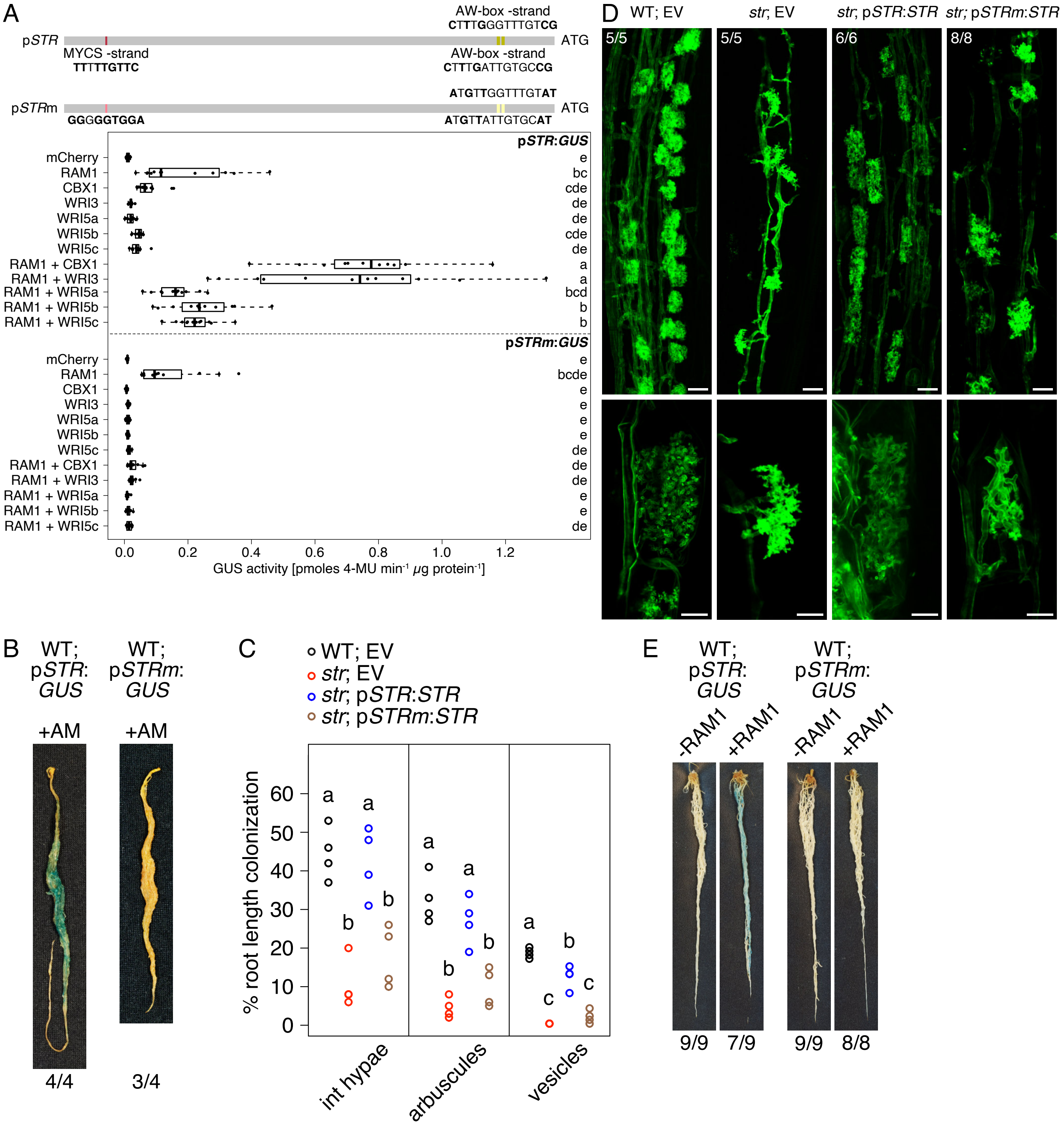

Fig. S1 WRI-target sites in the *STR* promoter are required for *STR* activation and arbuscule development. (*A*) Transactivation assay in *N. benthamiana* leaves. The p*STR*:*GUS* or p*STRm*:*GUS* reporter plasmids were co-transformed with plasmids containing the genomic sequence of the proteins indicated at the y-axis driven by the constitutive *LjUbiquitin10* promoter. The schematic representation on top illustrates the position and sequence of the *AW*-boxes (yellow marks) and *MYCS*-element (red mark) and their mutated versions (light yellow marks and light red marks, respectively) in the two promoters. Bold letters indicate the basepairs, defining the respective motif. Bold black line, median; box, interquartile range; whiskers highest and lowest data point within 1.5 interquartile range; dots, actual values. Different letters indicate different statistical groups (ANOVA; post hoc Tukey; n=12; $p < 0.05$). (*B*) Representative images of GUS activity resulting from p*STR* and p*STRm* activation in wild-type hairy roots colonized with *R. irregularis* at 6 wpi. Roots were stained with X-Gluc for 6h. The numbers indicate the number of root systems that displayed a staining as shown in the image, among the total number of analyzed root systems. (*C*) Root length colonization parameters of wild-type and *str* hairy roots, colonized with *R. irregularis* at 6 wpi, transformed with the indicated expression cassettes. Different letters indicate different statistical groups (ANOVA; post hoc Tukey; n=4; $p < 0.05$). *(D)* Representative laser scanning confocal images of wild-type and *str* hairy roots colonized by *R. irregularis* at 6 wpi, transformed with the indicated expression cassettes. The fungus is stained with WGA-Alexa-Fluor488. Scale bars: upper panel: 30 µm, lower panel: 10 µm. (*E*) Representative images of GUS activity resulting from p*STR* and p*STRm* activation in non-colonized wild-type hairy roots at 6 wpp. The plasmids included an additional empty vector (-RAM1) or p*Ubi*:*RAM1* (+RAM1) cassette. All roots were stained with X-Gluc for 6h. The numbers indicate the number of root systems that displayed a staining similar to the one, shown in the image, among the total number of analyzed root systems. (*A*-*E*) The experiments were performed once.

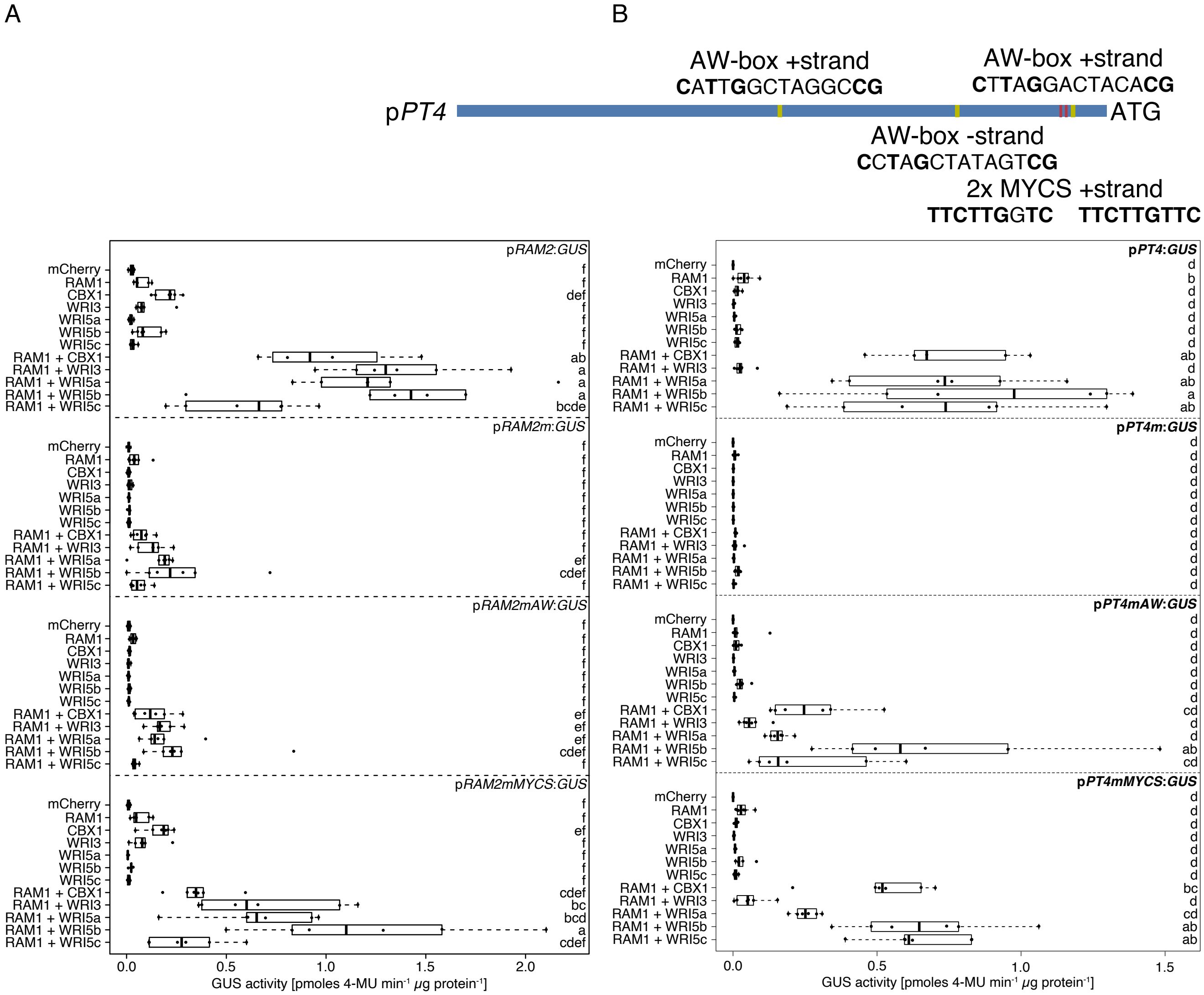

Fig. S2 WRI binding sites are required for transactivation of the *RAM2* and *PT4* promoters. Transactivation assay in *N. benthamiana* leaves. (*A*) The indicated p*RAM2* reporter plasmids were co-transformed with plasmids containing the genomic sequence encoding the proteins indicated at the y-axis driven by the constitutive *LjUbiquitin10* promoter. To generate p*RAM2m* the same mutations were applied to p*RAM2* as in Fig. 1*A*. To generate p*RAM2mAW* and p*RAM2mMYCS* only the *AW*-boxes or only the *MYCS*-elements were mutated, respectively. (*B*) The indicated p*PT4* reporter plasmids were co-transformed as in *(A)*. The location of *AW*-boxes and *MYCS*-elements and their mutations in the *PT4* promoter are shown in the schematic representation at the top. *(A, B)* Bold letters indicate the base pairs, defining the respective motif. Bold black line, median; box, interquartile range; whiskers highest and lowest data point within 1.5 interquartile range; dots, actual values. Different letters indicate different statistical groups (ANOVA; post hoc Tukey; n=6; p < 0.05). (*A,B*) The experiments were performed once.

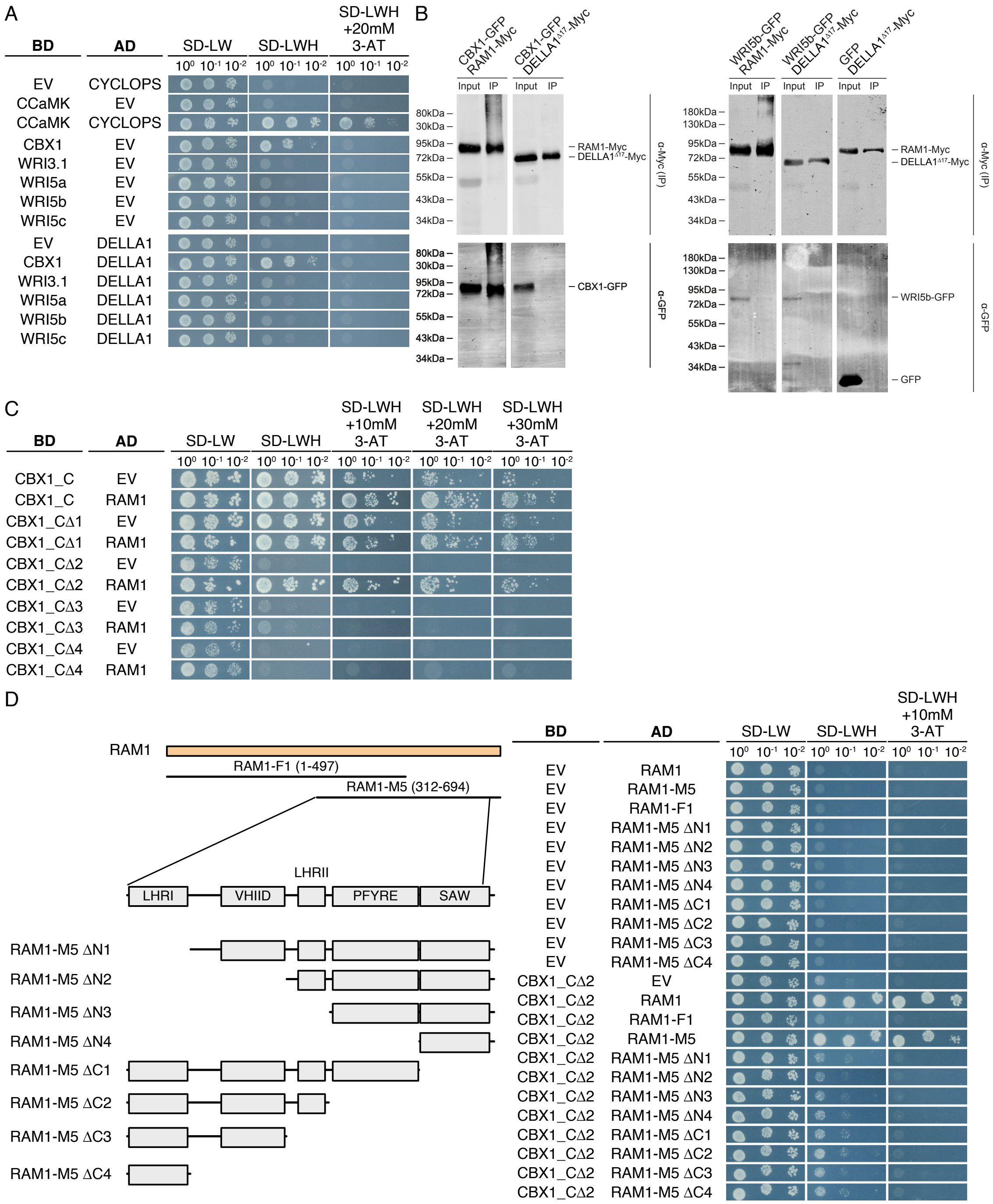

A
BD
AD
SD-LW
SD-LWH
SD-LWH +20mM 3-AT
EV CYCLOPS
CCaMK EV
CCaMK CYCLOPS
CBX1 EV
WRI3.1 EV
WRI5a EV
WRI5b EV
WRI5c EV
EV DELLA1
CBX1 DELLA1
WRI3.1 DELLA1
WRI5a DELLA1
WRI5b DELLA1
WRI5c DELLA1
B
CBX1-GFP RAM1-Myc
CBX1-GFP DELLA1Δ17-Myc
WRI5b-GFP RAM1-Myc
WRI5b-GFP DELLA1Δ17-Myc
GFP DELLA1Δ17-Myc
Input IP
– RAM1-Myc
– DELLA1Δ17-Myc
– CBX1-GFP
– WRI5b-GFP
– GFP
α-Myc (IP)
α-GFP
C
SD-LWH +10mM 3-AT
SD-LWH +30mM 3-AT
CBX1_C EV
CBX1_C RAM1
CBX1_CΔ1 EV
CBX1_CΔ1 RAM1
CBX1_CΔ2 EV
CBX1_CΔ2 RAM1
CBX1_CΔ3 EV
CBX1_CΔ3 RAM1
CBX1_CΔ4 EV
CBX1_CΔ4 RAM1
D
RAM1
RAM1-F1 (1-497)
RAM1-M5 (312-694)
LHRI
VHIID
LHRII
PFYRE
SAW
RAM1-M5 ΔN1
RAM1-M5 ΔN2
RAM1-M5 ΔN3
RAM1-M5 ΔN4
RAM1-M5 ΔC1
RAM1-M5 ΔC2
RAM1-M5 ΔC3
RAM1-M5 ΔC4
EV RAM1
EV RAM1-M5
EV RAM1-F1
CBX1_CΔ2 EV
CBX1_CΔ2 RAM1
CBX1_CΔ2 RAM1-F1
CBX1_CΔ2 RAM1-M5

Fig. S3 RAM1 but not DELLA1 interacts with AM-induced WRI transcription factors via its GRAS domain. *(A)* GAL4-based yeast two-hybrid assay to test interaction of DELLA1 (prey) and five AM-induced WRI transcription factors (bait). The well-established interaction between CCaMK and CYCLOPS (2) was used as a positive control and all coding-sequence-containing plasmids were used in combination with the complementary empty vector as negative controls. Transformed yeast clones were dropped at optical density 600(OD_{600}) = 0.6 (10^0), 0.06 (10^{-1}), and 0.006 (10^{-2}) on solid SD medium lacking Leu and Trp (-LW), lacking Leu, Trp, and His (-LWH) or lacking Leu, Trp, and His (-LWH) while additionally containing 20 mM 3-AT to suppress autoactivity. *(B)* Western Blot images of Co-immunoprecipitation assays showing interaction of CBX1 and WRI5b with RAM1 in *N. benthamiana* leaves. Fractions loaded are input (Input) and immunoprecipitation (IP). Molecular weights of the bands of the protein standard are indicated on the left side. Primary antibodies used for detection are indicated on the right side. *(C)* Replication of the experiment shown in Fig 3*B*, but with higher 3-AT concentrations. (*D*) GAL4-based yeast two-hybrid assay to test interaction of RAM1 and its truncated versions with CBX1. RAM1 and its truncated versions, illustrated in the schematic representation on the left, were used as prey (AD) and a truncated version of CBX1 (explanation in Fig. 3*B*) as bait (BD). All coding-sequence-containing plasmids were used in combination with the complementary empty vector as negative controls. Transformed yeast clones were dropped at optical density 600(OD_{600}) = 0.6 (10^0), 0.06 (10^{-1}), and 0.006 (10^{-2}) on solid SD medium lacking Leu and Trp (-LW), lacking Leu, Trp, and His (-LWH) or lacking Leu, Trp, and His (-LWH) while additionally containing 10 mM 3-AT to suppress autoactivity. Except (*D*), which was performed once, all experiments were performed twice with similar results.

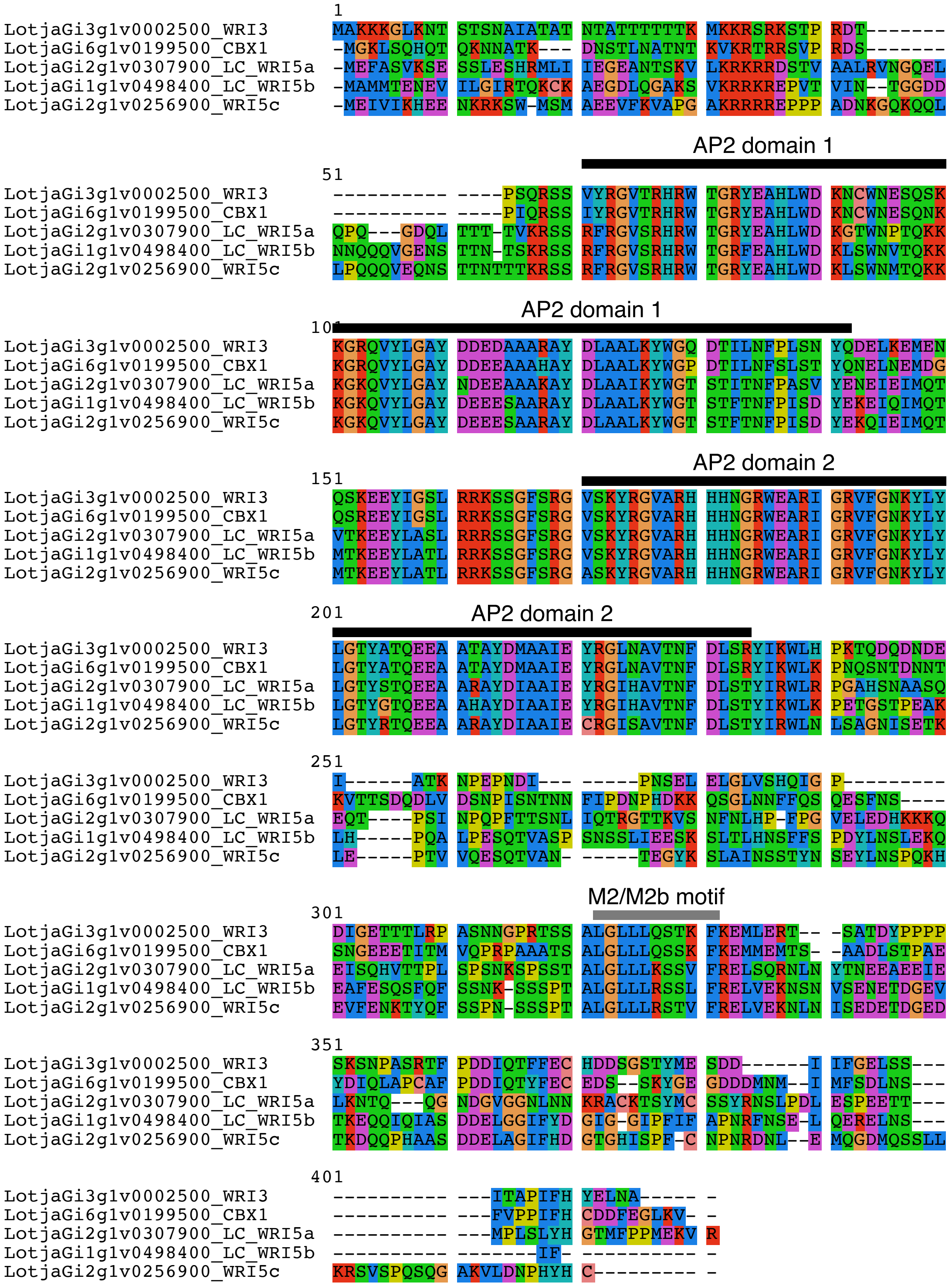
1
LotjaGi3g1v0002500_WRI3 MAKKKGLKNT STSNAIATAT NTATTTTTTK MKKRSRKSTP RDT-------
LotjaGi6g1v0199500_CBX1 -MGKLSQHQT QKNNATK--- DNSTLNATNT KVKRTRRSVP RDS-------
LotjaGi2g1v0307900_LC_WRI5a -MEFASVKSE SSLESHRMLI IEGEANTSKV LKRKRRDSTV AALRVNGQEL
LotjaGi1g1v0498400_LC_WRI5b -MAMMTENEV ILGIRTQKCK AEGDLQGAKS VKRRKREPVT VIN--TGGDD
LotjaGi2g1v0256900_WRI5c -MEIVIKHEE NKRKSW-MSM AEEVFKVAPG AKRRRREPPP ADNKGQKQQL
AP2 domain 1
51
LotjaGi3g1v0002500_WRI3 ---------- ----PSQRSS VYRGVTRHRW TGRYEAHLWD KNCWNESQSK
LotjaGi6g1v0199500_CBX1 ---------- ----PIQRSS IYRGVTRHRW TGRYEAHLWD KNCWNESQNK
LotjaGi2g1v0307900_LC_WRI5a QPQ---GDQL TTT-TVKRSS RFRGVSRHRW TGRYEAHLWD KGTWNPTQKK
LotjaGi1g1v0498400_LC_WRI5b NNQQQVGENS TTN-TSKRSS RFRGVSRHRW TGRFEAHLWD KLSWNVTQKK
LotjaGi2g1v0256900_WRI5c LPQQQVEQNS TTNTTTKRSS RFRGVSRHRW TGRYEAHLWD KLSWNMTQKK
AP2 domain 1
101
LotjaGi3g1v0002500_WRI3 KGRQVYLGAY DDEDAAARAY DLAALKYWGQ DTILNFPLSN YQDELKEMEN
LotjaGi6g1v0199500_CBX1 KGRQVYLGAY DDEEAAAHAY DLAALKYWGP DTILNFSLST YQNELNEMDG
LotjaGi2g1v0307900_LC_WRI5a KGKQVYLGAY NDEEAAAKAY DLAAIKYWGT STITNFPASV YENEIEIMQT
LotjaGi1g1v0498400_LC_WRI5b KGKQVYLGAY DEEESAARAY DLAALKYWGT STFTNFPISD YEKEIQIMQT
LotjaGi2g1v0256900_WRI5c KGKQVYLGAY DEEESAARAY DLAALKYWGT STFTNFPISD YEKQIEIMQT
AP2 domain 2
151
LotjaGi3g1v0002500_WRI3 QSKEEYIGSL RRKSSGFSRG VSKYRGVARH HHNGRWEARI GRVFGNKYLY
LotjaGi6g1v0199500_CBX1 QSREEYIGSL RRKSSGFSRG VSKYRGVARH HHNGRWEARI GRVFGNKYLY
LotjaGi2g1v0307900_LC_WRI5a VTKEEYLASL RRRSSGFSRG VSKYRGVARH HHNGRWEARI GRVFGNKYLY
LotjaGi1g1v0498400_LC_WRI5b MTKEEYLATL RRKSSGFSRG VSKYRGVARH HHNGRWEARI GRVFGNKYLY
LotjaGi2g1v0256900_WRI5c MTKEEYLATL RRKSSGFSRG ASKYRGVARH HHNGRWEARI GRVFGNKYLY
201
AP2 domain 2
LotjaGi3g1v0002500_WRI3 LGTYATQEEA ATAYDMAAIE YRGLNAVTNF DLSRYIKWLH PKTQDQDNDE
LotjaGi6g1v0199500_CBX1 LGTYATQEEA ATAYDMAAIE YRGLNAVTNF DLSRYIKWLK PNQSNTDNNT
LotjaGi2g1v0307900_LC_WRI5a LGTYSTQEEA ARAYDIAAIE YRGIHAVTNF DLSTYIRWLR PGAHSNAASQ
LotjaGi1g1v0498400_LC_WRI5b LGTYGTQEEA AHAYDIAAIE YRGIHAVTNF DLSTYIKWLK PETGSTPEAK
LotjaGi2g1v0256900_WRI5c LGTYRTQEEA ARAYDIAAIE CRGISAVTNF DLSTYIRWLN LSAGNISETK
251
LotjaGi3g1v0002500_WRI3 I------ATK NPEPNDI--- -----PNSEL ELGLVSHQIG P---------
LotjaGi6g1v0199500_CBX1 KVTTSDQDLV DSNPISNTNN FIPDNPHDKK QSGLNNFFQS QESFNS----
LotjaGi2g1v0307900_LC_WRI5a EQT----PSI NPQPFTTSNL IQTRGTTKVS NFNLHP-FPG VELEDHKKKQ
LotjaGi1g1v0498400_LC_WRI5b LH-----PQA LPESQTVASP SNSSLIEESK SLTLHNSFFS PDYLNSLEKQ
LotjaGi2g1v0256900_WRI5c LE-----PTV VQESQTVAN- -----TEGYK SLAINSSTYN SEYLNSPQKH
M2/M2b motif
301
LotjaGi3g1v0002500_WRI3 DIGETTTLRP ASNNGPRTSS ALGLLLQSTK FKEMLERT-- -SATDYPPPP
LotjaGi6g1v0199500_CBX1 SNGEEETITM VQPRPAAATS ALGLLLQSSK FKEMMEMTS- -AADLSTPAE
LotjaGi2g1v0307900_LC_WRI5a EISQHVTTPL SPSNKSPSST ALGLLLKSSV FRELSQRNLN YTNEEAEEIE
LotjaGi1g1v0498400_LC_WRI5b EAFESQSFQF SSNK-SSSPT ALGLLLRSSL FRELVEKNSN VSENETDGEV
LotjaGi2g1v0256900_WRI5c EVFENKTYQF SSPN-SSSPT ALGLLLRSTV FRELVEKNLN ISEDETDGED
351
LotjaGi3g1v0002500_WRI3 SKSNPASRTF PDDIQTFFEC HDDSGSTYME SDD------I IFGELSS---
LotjaGi6g1v0199500_CBX1 YDIQLAPCAF PDDIQTYFEC EDS--SKYGE GDDDMNM--I MFSDLNS---
LotjaGi2g1v0307900_LC_WRI5a LKNTQ---QG NDGVGGNLNN KRACKTSYMC SSYRNSLPDL ESPEETT---
LotjaGi1g1v0498400_LC_WRI5b TKEQQIQIAS DDELGGIFYD GIG-GIPFIF APNRFNSE-L QERELNS---
LotjaGi2g1v0256900_WRI5c TKDQQPHAAS DDELAGIFHD GTGHISPF-C NPNRDNL--E MQGDMQSSLL
401
LotjaGi3g1v0002500_WRI3 ---------- ---ITAPIFH YELNA----- -
LotjaGi6g1v0199500_CBX1 ---------- ---FVPPIFH CDDFEGLKV- -
LotjaGi2g1v0307900_LC_WRI5a ---------- ---MPLSLYH GTMFPPMEKV R
LotjaGi1g1v0498400_LC_WRI5b ---------- -------IF- ---------- -
LotjaGi2g1v0256900_WRI5c KRSVSPQSQG AKVLDNPHYH C--------- -

Fig. S4 Full length amino acid sequence alignment of CBX1, WRI3, WRI5a, WRI5b, WRI5c. Alignment of the amino acid sequences of the five AM-induced WRI transcription factors of *L. japonicus*. Positions of the AP2 domains and the M2/M2b motifs are highlighted with black and grey bars respectively. The color code marks amino acids with similar physico-chemical properties (blue nonpolar, green polar, red basic, purple acidic, turquoise aromatic, yellow proline, orange glycin, rose cystein). Dashes show gaps in alignment. The alignment was performed in seaview5 (24) with the muscle algorithm (23).

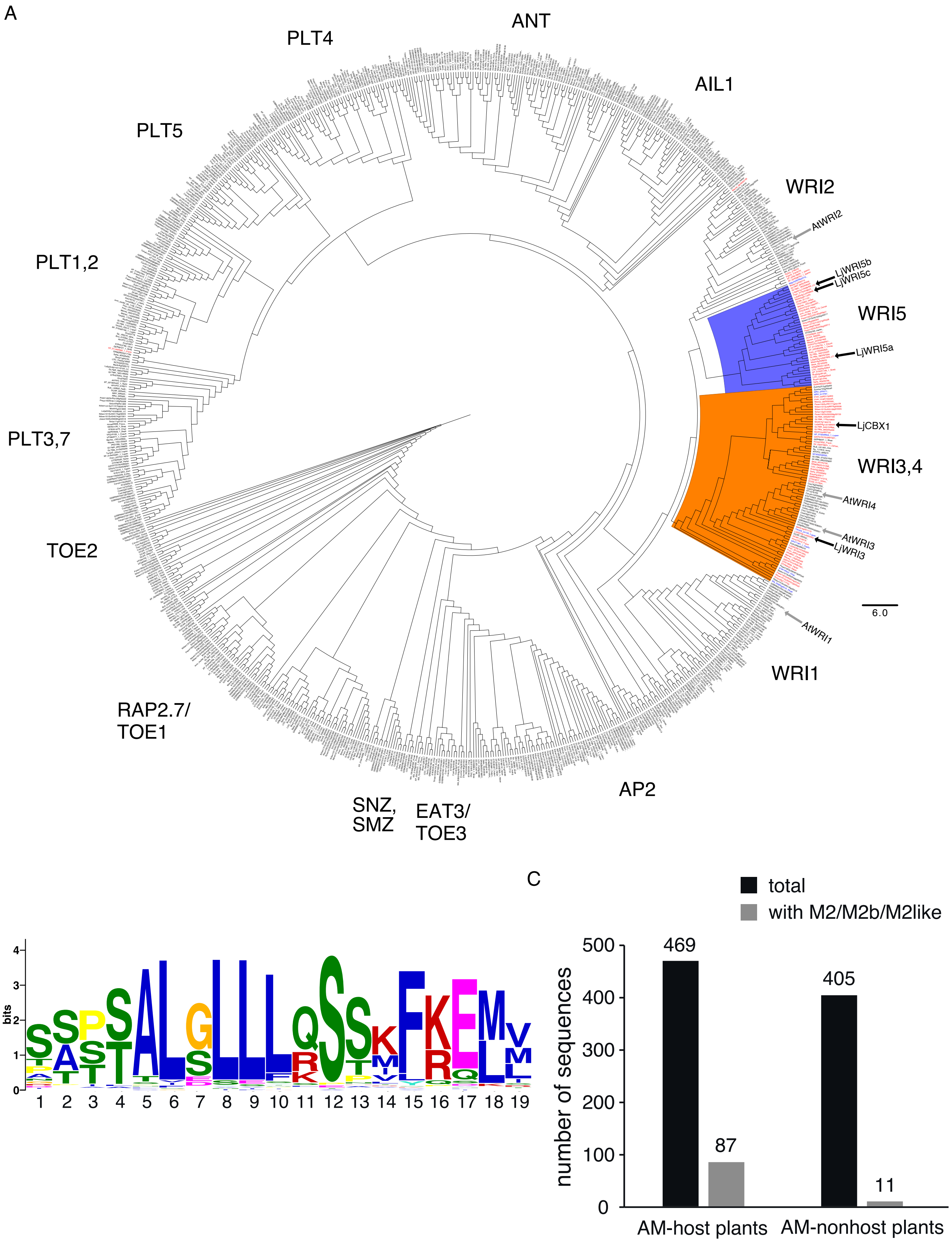
A
ANT
PLT4
AIL1
PLT5
WRI2
AtWRI2
PLT1,2
LjWRI5b
LjWRI5c
WRI5
LjWRI5a
LjCBX1
PLT3,7
WRI3,4
AtWRI4
AtWRI3
LjWRI3
TOE2
6.0
AtWRI1
WRI1
RAP2.7/
TOE1
AP2
SNZ,
SMZ
EAT3/
TOE3
B
bits
4
3
2
1
0
1 2 3 4 5 6 7 8 9 10 11 12 13 14 15 16 17 18 19
C
total
with M2/M2b/M2like
number of sequences
500
400
300
200
100
0
469
87
405
11
AM-host plants
AM-nonhost plants

Fig. S5 The M2 motif is present more frequently in WRI protein sequences of AM host plants than of AM-nonhost plants. (*A*) Cladogram of WRI protein sequences (model: VT +G+F, log-likelihood: -661244.38524). Red sequence identifiers indicate sequences from AM-host plants containing the M2/M2b/M2-like motif, blue sequence identifiers indicate sequences from AM-nonhost plants containing the M2/M2b/M2-like motif. Clades of the tree are named after representative sequences of *A. thaliana* (grey arrows) or *L. japonicus* (black arrows). In addition, the WRI3,4 clade is highlighted in orange and the WRI5 clade is highlighted in purple. (*B*) Frequence-position plot of the M2/M2b/M2-like motif generated with the 98 sequences highlighted in red and blue in Figure S3a, generated with the MEME tool (29) of meme-suite.org (30). (*C*) Barplot comparing the amount WRI protein sequences of 18 AM-host plants vs. 21 AM-non-host plants and the number of sequences containing the M2/M2b/M2-like motif.

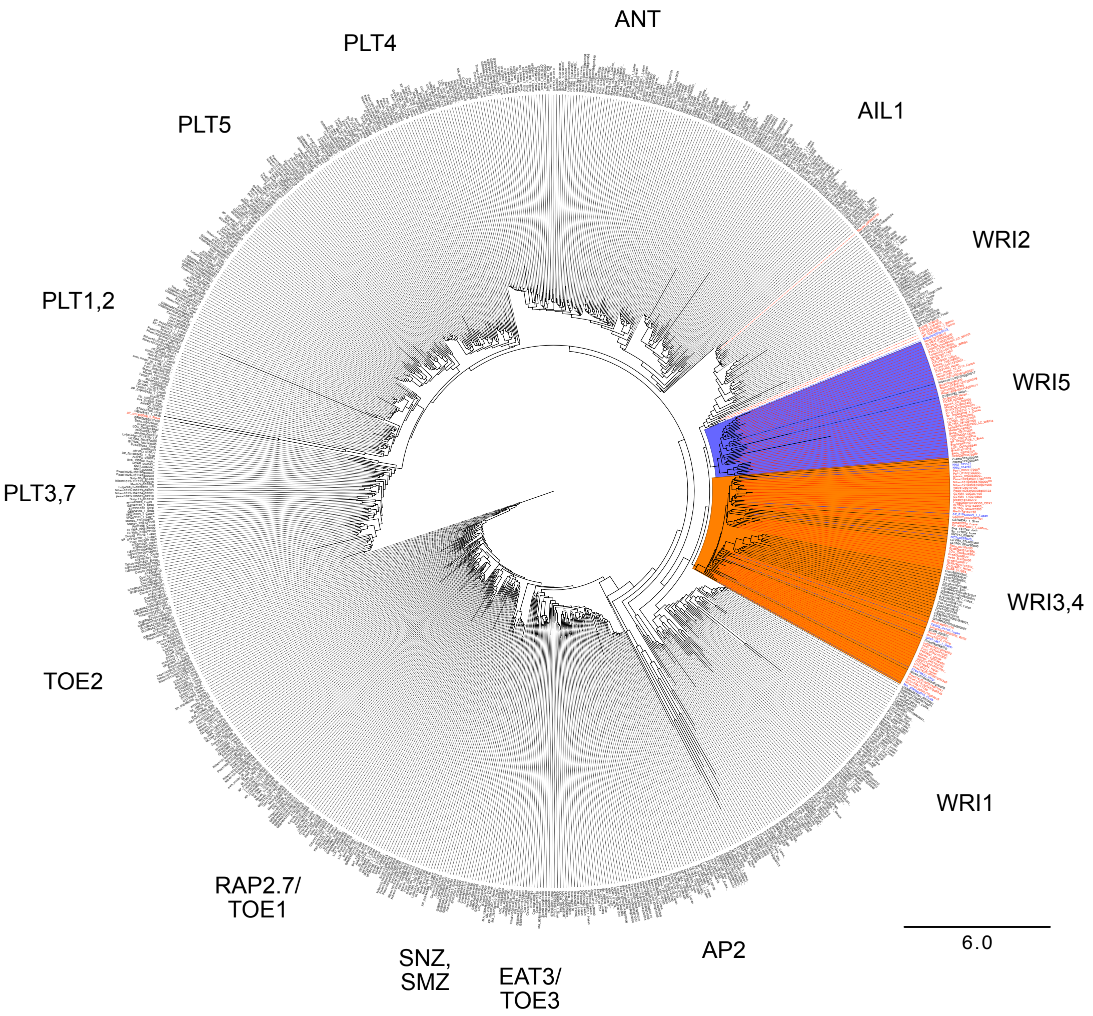

Fig. S6 The M2 motif is present more frequently in WRI protein sequences of AM host plants than of AM-nonhost plants. Phylogenetic tree of WRI protein sequences (model: VT +G+F, log-likelihood: -661244.38524), providing the basis for the cladogram in Figure S5. Red sequence identifiers indicate sequences from AM-host plants containing the M2/M2b/M2-like motif, blue sequence identifiers indicate sequences from AM-nonhost plants containing the M2/M2b/M2-like motif. Clades of the tree are named after representative sequences of *A. thaliana*. The WRI3,4 clade is highlighted in orange and the WRI5 clade is highlighted in purple. Scale bar, number of amino acid substitutions per site.

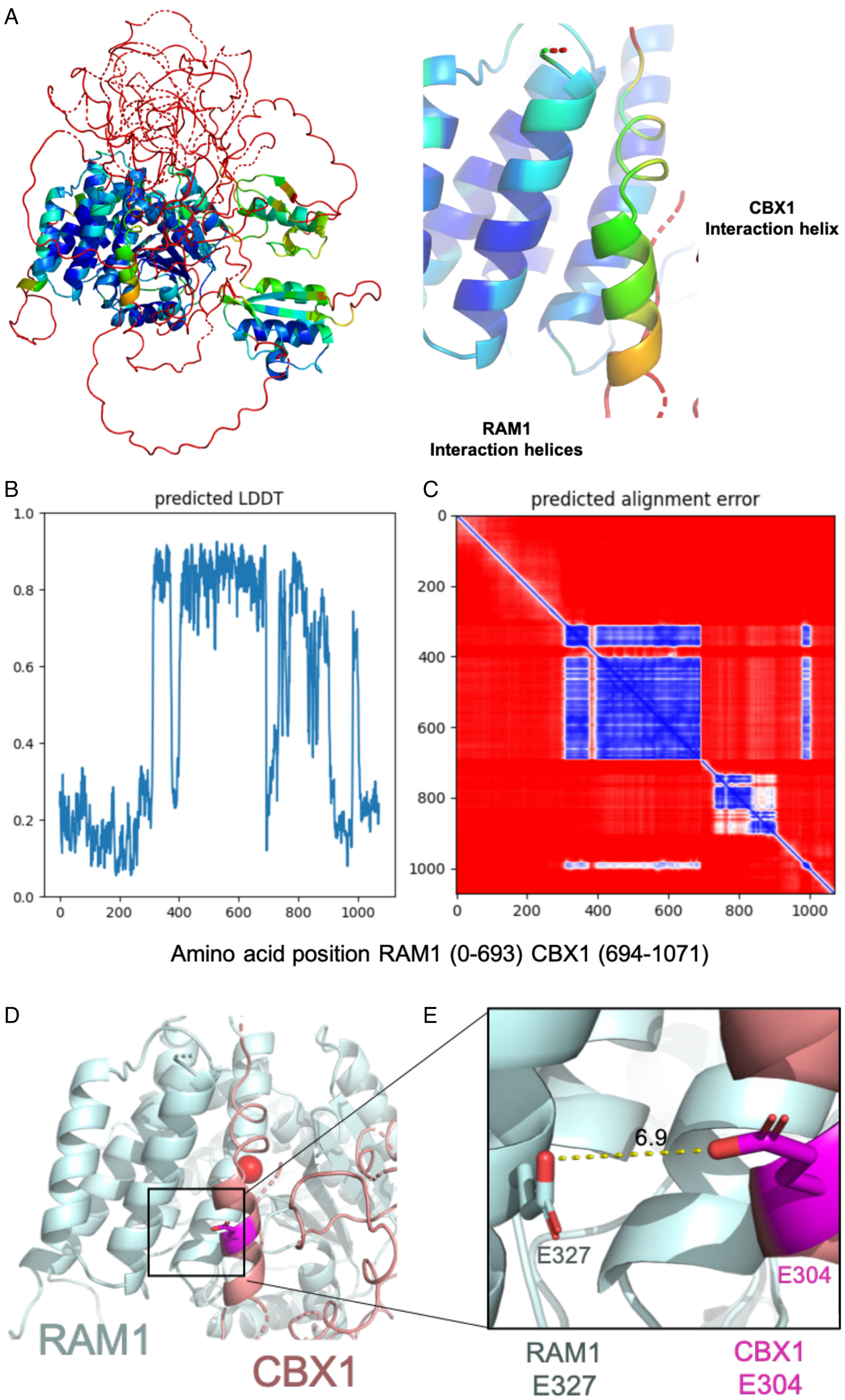
A
CBX1
Interaction helix
RAM1
Interaction helices
B
predicted LDDT
C
predicted alignment error
Amino acid position RAM1 (0-693) CBX1 (694-1071)
D
E
6.9
E327
E304
RAM1
CBX1
RAM1
E327
CBX1
E304

Fig. S7 Quality metrics of CBX1-RAM1 complex Structure *(A)* RoseTTaFold predicted CBX1-RAM1 complex shown as cartoon colored by per-residue confidence scores predicted local distance difference test (pLDDT, blue color indicates high confidence and red indicates low confidence scores). *(B-C)* the scores of pLDDT (*B*) and predicted alignment error (PAE*, C*) are plotted across each residue. The CBX1 interacting helix (interaction helix, 986-995), is highlighted in cyan star. Zoom in on the predicted interface between RAM1 (light blue) and CBX1 (salmon) represented as cartoon, and close up view highlighting E327 (of RAM1) and E304 (of CBX1) is shown in *(D)*.

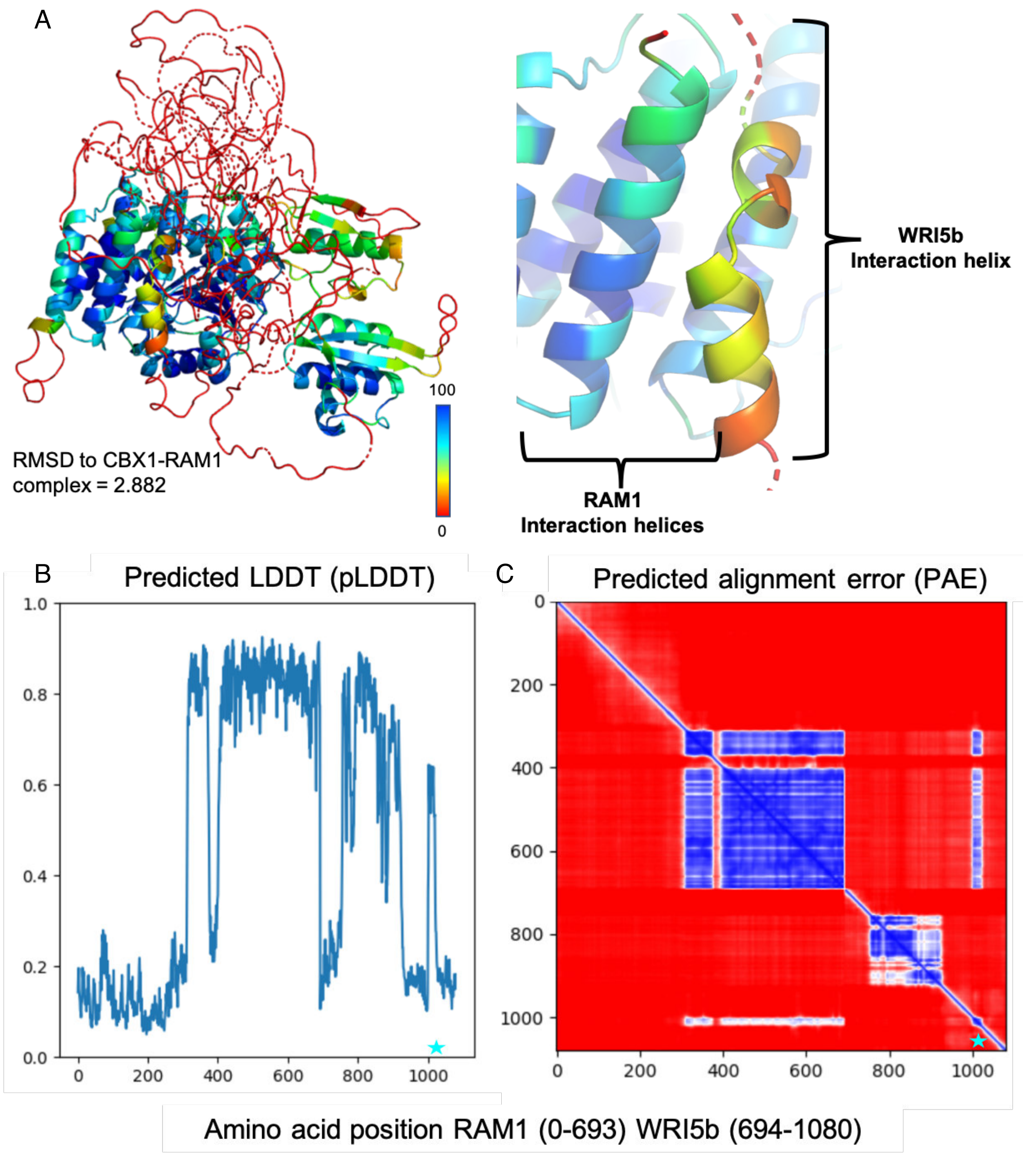

Figure S8 Quality metrics of WRI5b-RAM1 complex structure (*A*) RoseTTaFold predicted WRI5b-RAM1 complex shown in cartoon colored by per-residue confidence scores predicted local distance difference test (pLDDT, blue color indicates high confidence and red indicates low confidence scores). (*B-C*) the scores of pLDDT (B) and predicted alignment error (PAE*, C*) are plotted across each residue. The WRI5b interacting helix (interaction helix, 1005-1014), is highlighted in cyan star. Root mean square deviation (RMSD = 2.882Å) is calculated for Cα comparing the WRI5B-RAM1 complex with the entire CBX1-RAM1 complex.

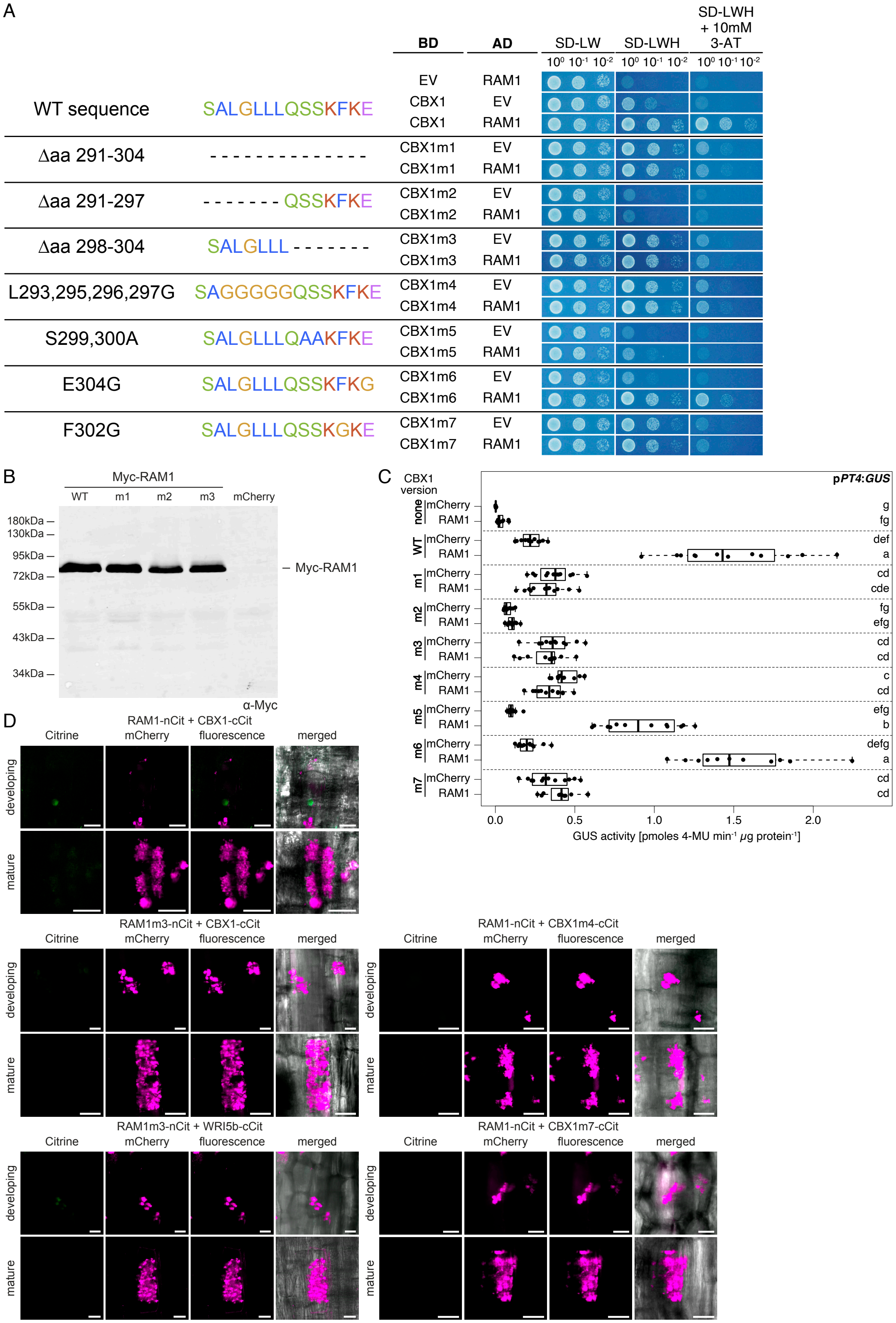
A
BD
AD
SD-LW
SD-LWH
SD-LWH + 10mM 3-AT
WT sequence
SALGLLLQSSKFKE
EV RAM1
CBX1 EV
CBX1 RAM1
Δaa 291-304
CBX1m1 EV
CBX1m1 RAM1
Δaa 291-297
QSSKFKE
CBX1m2 EV
CBX1m2 RAM1
Δaa 298-304
SALGLLL
CBX1m3 EV
CBX1m3 RAM1
L293,295,296,297G
SAGGGGGQSSKFKE
CBX1m4 EV
CBX1m4 RAM1
S299,300A
SALGLLLQAAKFKE
CBX1m5 EV
CBX1m5 RAM1
E304G
SALGLLLQSSKFKG
CBX1m6 EV
CBX1m6 RAM1
F302G
SALGLLLQSSKGKE
CBX1m7 EV
CBX1m7 RAM1
B
Myc-RAM1
WT m1 m2 m3 mCherry
180kDa
130kDa
95kDa
72kDa
55kDa
43kDa
34kDa
Myc-RAM1
α-Myc
C
CBX1 version
pPT4:GUS
none WT m1 m2 m3 m4 m5 m6 m7
mCherry
RAM1
g fg def a cd cde fg efg cd cd c cd efg b defg a cd cd
0.0 0.5 1.0 1.5 2.0
GUS activity [pmoles 4-MU min-1 µg protein-1]
D
RAM1-nCit + CBX1-cCit
RAM1m3-nCit + CBX1-cCit
RAM1-nCit + CBX1m4-cCit
RAM1m3-nCit + WRI5b-cCit
RAM1-nCit + CBX1m7-cCit
Citrine
mCherry
fluorescence
merged
developing
mature

Fig. S9 Mutations of CBX1 residues predicted to be involved in the interaction with RAM1 reduce interaction capability. (*A*) GAL4-based yeast two-hybrid assay to test interaction of RAM1 as prey (AD) and CBX1 and 7 CBX1 mutant versions as bait (BD). The schematic representation on the left illustrates the mutations and deletions applied to the M2/M2b motif of CBX1. All coding-sequence-containing plasmids were used in combination with the complementary empty vector (EV) as negative controls. Transformed yeast clones were dropped at optical density 600 (OD_{600}) = 0.6 (10^0), 0.06 (10^{-1}), and 0.006 (10^{-2}) on solid SD medium lacking Leu and Trp (-LW), lacking Leu, Trp, and His (-LWH) or lacking Leu, Trp, and His (-LWH) and additionally containing 10 mM 3-AT to suppress autoactivity. (*B*) Western Blot image of *N. benthaminana* protein extracts from transactivation assays (Fig. 5*B*) showing accumulation and stability of the three RAM1 mutant versions in *N. benthamiana* leaves. (*C*) Transactivation assay in *N. benthamiana* leaves. The p*PT4*:*GUS* reporter plasmid was co-transformed with plasmids containing the genomic sequence encoding the proteins indicated at the y-axis driven by the constitutive *LjUbiquitin10* promoter. Different mutants of CBX1 are separated by dashed lines. 4-MU, 4-methylumbelliferone. Bold black line, median; box, interquartile range; whiskers highest and lowest data point within 1.5 interquartile range; dots, actual values. Different letters indicate different statistical groups (ANOVA; post hoc Tukey; n=12; $p < 0.05$). (*A*-*C*) The experiments were performed once. (*D*) Analysis for interaction of RAM1m3 with CBX1 and WRI5b and CBX1m4 and m7 with RAM1 using bimolecular fluorescence complementation in arbuscule-containing cortex cells *L. japonicus* hairy roots at 5wpi. Green fluorescence indicates interaction. Magenta fluorescence indicates arbuscules. The upper panel shows a developing arbuscule, the lower panel a mature arbuscule. Overlays of confocal and bright field images are shown. Size bars, 20 µm. nCit, N-terminal half of Citrine; cCit, C-terminal half of Citrine. See Supplementary Table 1 for the number of observed cells.

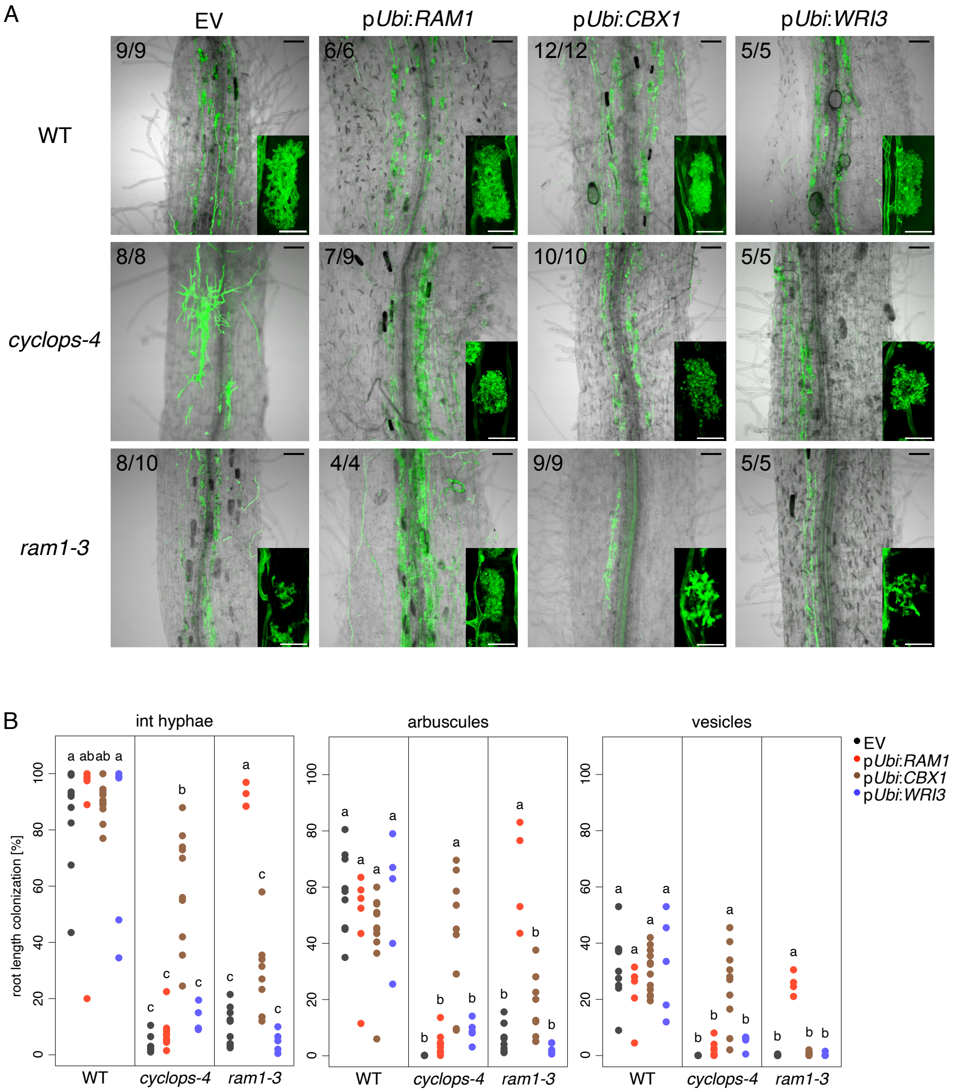

Fig. S10 Ectopic expression of *CBX1* and *WRI3* can restore arbuscule formation in *cyclops. (A)* Representative laser scanning confocal images of wild-type, *cyclops-4* and *ram1-3* hairy roots colonized with *R. irregularis* at 6 wpi, transformed with the indicated expression cassettes. The numbers indicate the number of root systems that displayed the phenotype shown in the image, among the total number of analyzed root systems. Black scale bar, 100 µm. Insets show a close-up of arbuscules; white scale bar, 20 µm. The fungus is stained with WGA-Alexa-Fluor488. *(B)* Root length colonization of wild-type, *cyclops-4* and *ram1-3* hairy roots, colonized with *R. irregularis* at 6 wpi, from (*A*). Different letters indicate different statistical groups (ANOVA; post hoc Tukey; $p < 0.05$). The experiment was performed once.

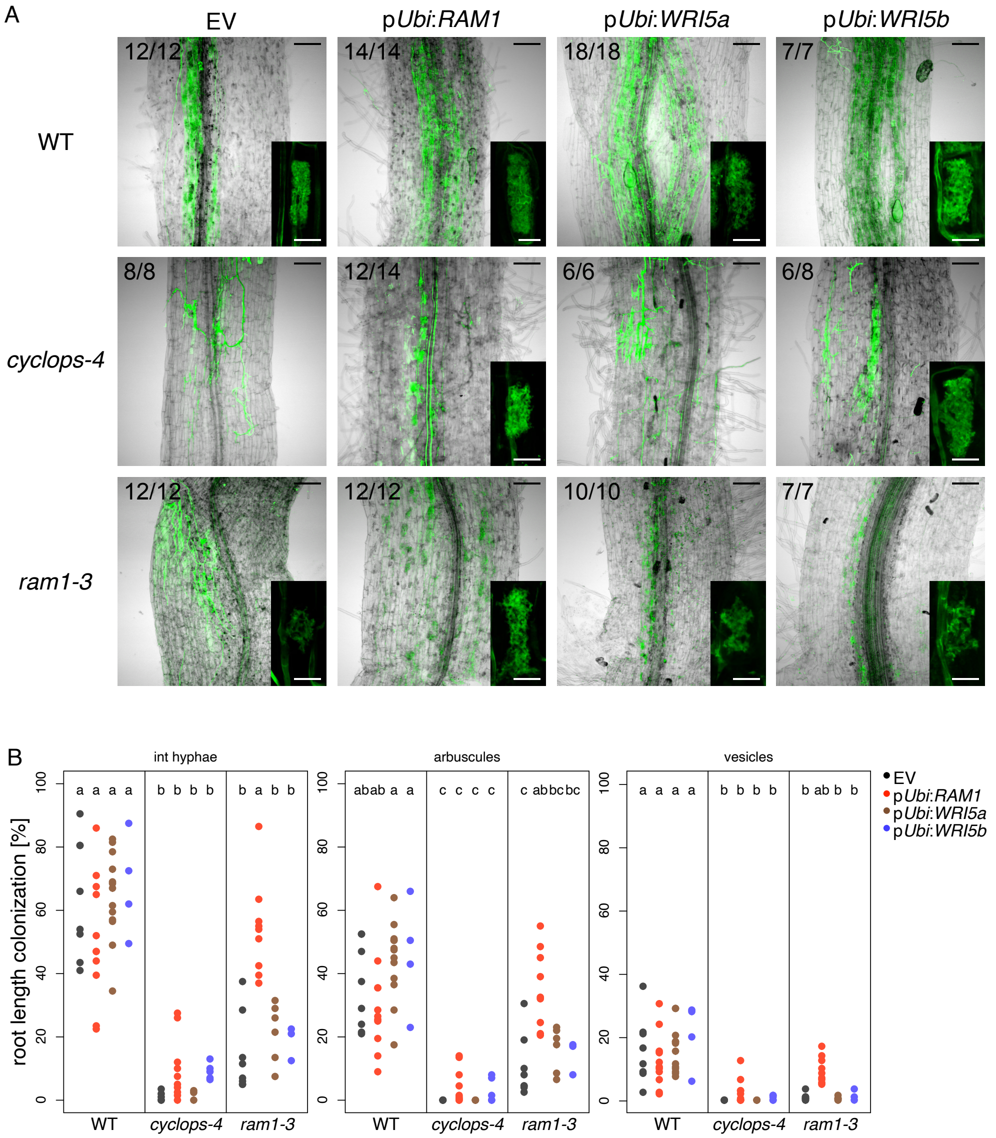

Fig. S11 Ectopic expression of *WRI5b* can restore arbuscule formation in *cyclops. (A)* Representative laser scanning confocal images of wild-type, *cyclops-4* and *ram1-3* hairy roots colonized with *R. irregularis* at 6 wpi, transformed with the indicated expression cassettes. The numbers indicate the number of root systems that displayed the phenotype shown in the image, among the total number of analyzed root systems. Note that each hairy root in a root system, results from an independent transformation event and that the phenotype was observed in all transformed and colonized roots. Black scale bar, 100 µm. Insets show a close-up of arbuscules; white scale bar, 20 µm. The fungus is stained with WGA-Alexa-Fluor488. *(B)* Root length colonization of wild-type, *cyclops-4* and *ram1-3* hairy roots, containing the indicated expression cassettes, colonized with *R. irregularis* at 6 wpi, from (*A*). Different letters indicate different statistical groups (ANOVA; post hoc Tukey; $p < 0.05$). The experiment was performed once.

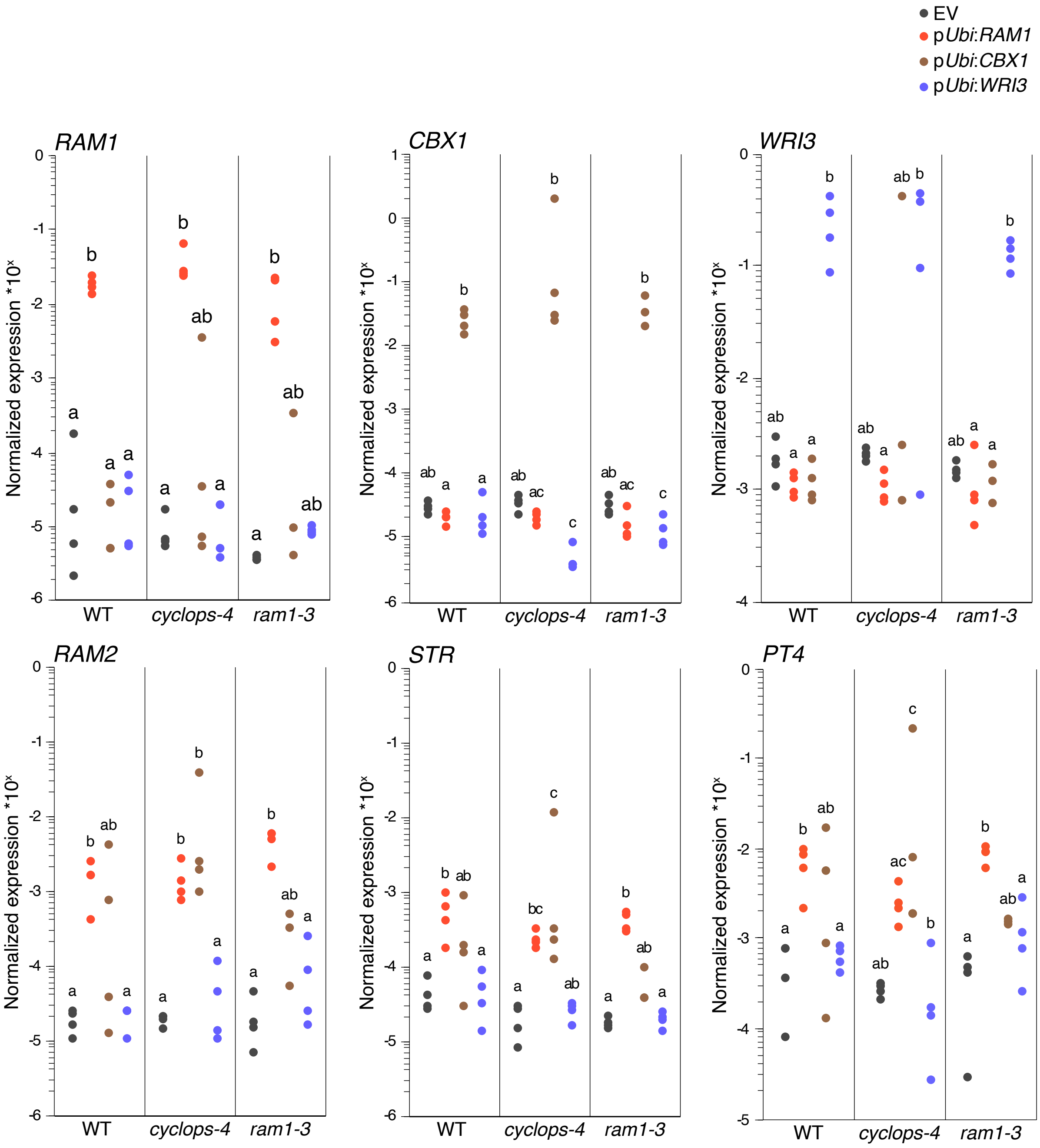

Fig. S12 *RAM2*, *STR* and *PT4* induction by *CBX1* overexpression depends on *RAM1.* Transcript accumulation of *RAM1*, *CBX1*, *WRI3*, *RAM2*, *STR* and *PT4* in non-colonized *L. japonicus* wild-type, *cyclops-4* and *ram1-3* hairy roots at 6 wpp, transformed with the indicated expression cassettes. Transcript accumulation was determined by RT-qPCR, and the housekeeping gene *Ubiquitin10* was used for normalization. Different letters indicate different statistical groups (Kruskal-Wallis; post hoc Dunn's multiple comparison test; n = 4 except *ram1-3*; p*Ubi:RAM1* n = 3; $p < 0.05$). The experiment was performed once.

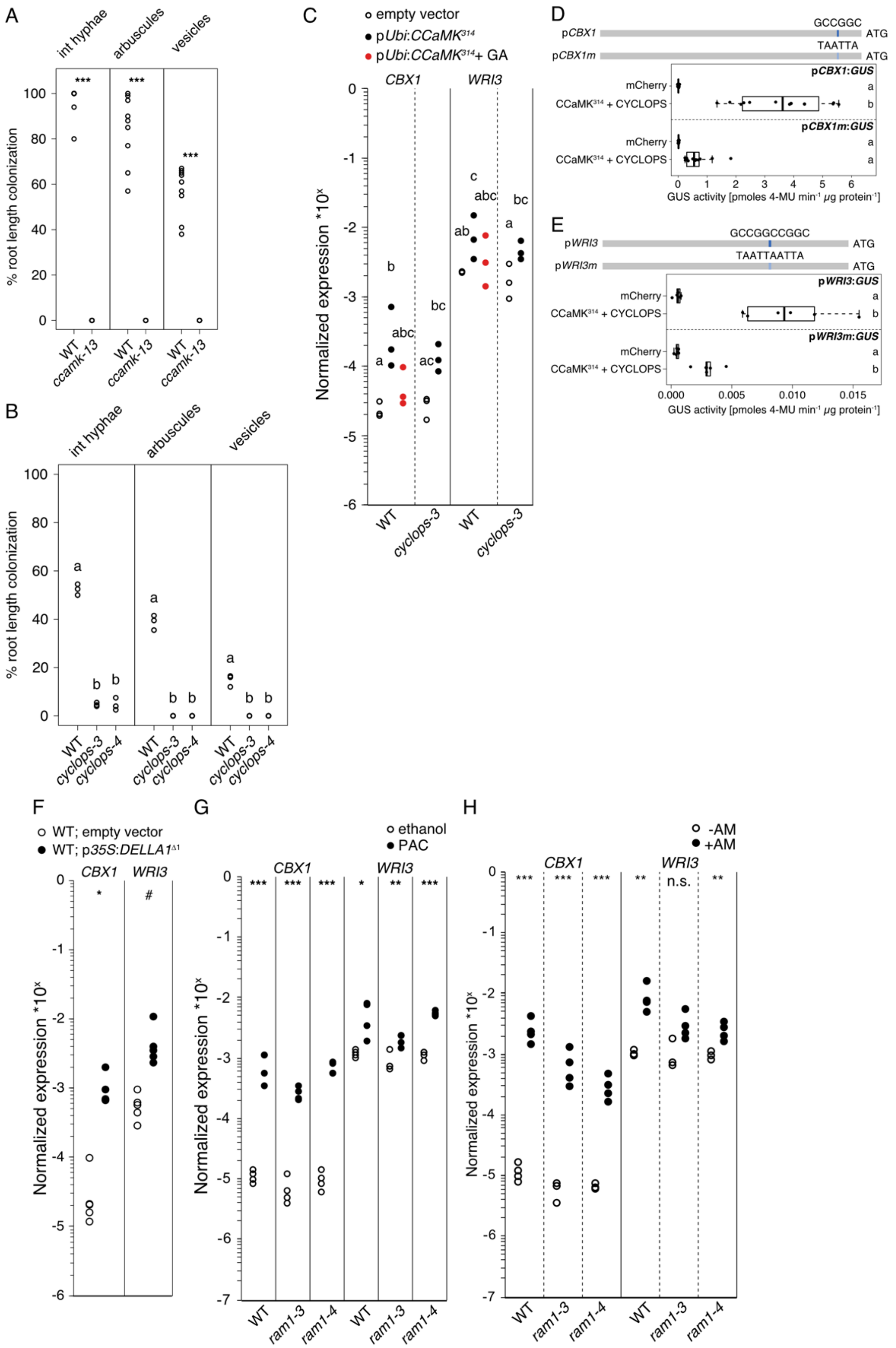
A
int hyphae
arbuscules
vesicles
% root length colonization
WT
ccamk-13
B
cyclops-3
cyclops-4
C
empty vector
pUbi:CCaMK314
pUbi:CCaMK314+ GA
CBX1
WRI3
Normalized expression *10x
D
pCBX1
pCBX1m
GCCGGC
TAATTA
ATG
pCBX1:GUS
pCBX1m:GUS
mCherry
CCaMK314 + CYCLOPS
GUS activity [pmoles 4-MU min-1 µg protein-1]
E
pWRI3
pWRI3m
GCCGGCCGGC
TAATTAATTA
pWRI3:GUS
pWRI3m:GUS
F
WT; empty vector
WT; p35S:DELLA1Δ1
G
ethanol
PAC
ram1-3
ram1-4
H
-AM
+AM
n.s.

Fig. S13 Transcript accumulation of *CBX1* and *WRI3* does not depend on *RAM1.* (A) Root length colonization of wild-type and *ccamk-13* roots, colonized with *R. irregularis* at 6 wpi, corresponding to Fig. 6*A* and S14*B*. Different letters indicate different statistical groups (ANOVA; post hoc Tukey; n=9; $p < 0.05$). (*B*) Root length colonization of wild-type, *cyclops-3* and *cyclops-4* roots, colonized with *R. irregularis* at 6 wpi, corresponding to RT-qPCR data in Fig. 6*B* and S14*C*. Different letters indicate different statistical groups (ANOVA; post hoc Tukey; n=3; $p < 0.05$*).* (*C*) Transcript accumulation of *CBX1* and *WRI3* in non-colonized wild-type and *cyclops-3* hairy roots transformed with an empty vector (EV) or with p*Ubi*:*CCaMK*^{314}*-NLS* at 5 wpp. Roots were treated with solvent (0.002% ethanol) or 1 mM GA_3. Treatment started at 1 wpp. Different letters indicate different statistical groups (Kruskal-Wallis; post hoc Dunn; n = 3; $p < 0.05$). (*D, E*) Transactivation assay of CBX1 (*D*) and WRI3 (*E*) promoters in *N. benthamiana* leaves. Coding sequences of the proteins indicated at the y-axis are driven by the *35S* promoter. The schematic representation on top illustrates the position and sequence of the *AM-CYCREs* (blue mark) and the mutated *AM-CYCREs* (light blue mark) in each of the two promoters, respectively. Bold letters indicate the base pairs, defining the respective motif. Bold black line, median; box, interquartile range; whiskers highest and lowest data point within 1.5 interquartile range; dots, actual values. Different letters indicate different statistical groups (ANOVA; post hoc Tukey; n=12 (*C*); n=6 (*D*); $p < 0.05$). (*F*) Transcript accumulation of *CBX1* and *WRI3* in wild-type hairy roots transformed with an empty vector (EV) or a p*35S*:□^{17}*DELLA1* expression cassette at 5 wpp. Statistical analysis: Welch t-test (n=5, # $p<0.1$, * $p < 0.05$*).* (*G*) Transcript accumulation of *CBX1* and *WRI3* in non-colonized wild-type and *ram1-3* roots at 5 wpp. Roots were treated with solvent (0.01% ethanol) or 1 µM PAC. Treatment started at 1 wpp. Statistical analysis: Welch t-test (n=3, * $p < 0.05$, *** p<0,001). (*H*) Transcript accumulation of *CBX1* and *WRI3* in wild-type, *ram1-3* and *ram1-4* roots colonized by *R. irregularis* at 5 wpi. Corresponding root colonization data are displayed in Fig. S14*A.* Statistical analysis: Welch t-test (n=3, # $p<0.1$, * $p < 0.05$). The experiment was performed twice with similar results. (*C,F-H)* Transcript accumulation was determined by RT-qPCR, and expression of the housekeeping gene *Ubiquitin10* was used for normalization. (*A*, *B*, F, G, *H*) The experiments were performed twice with similar results, all others were performed once.

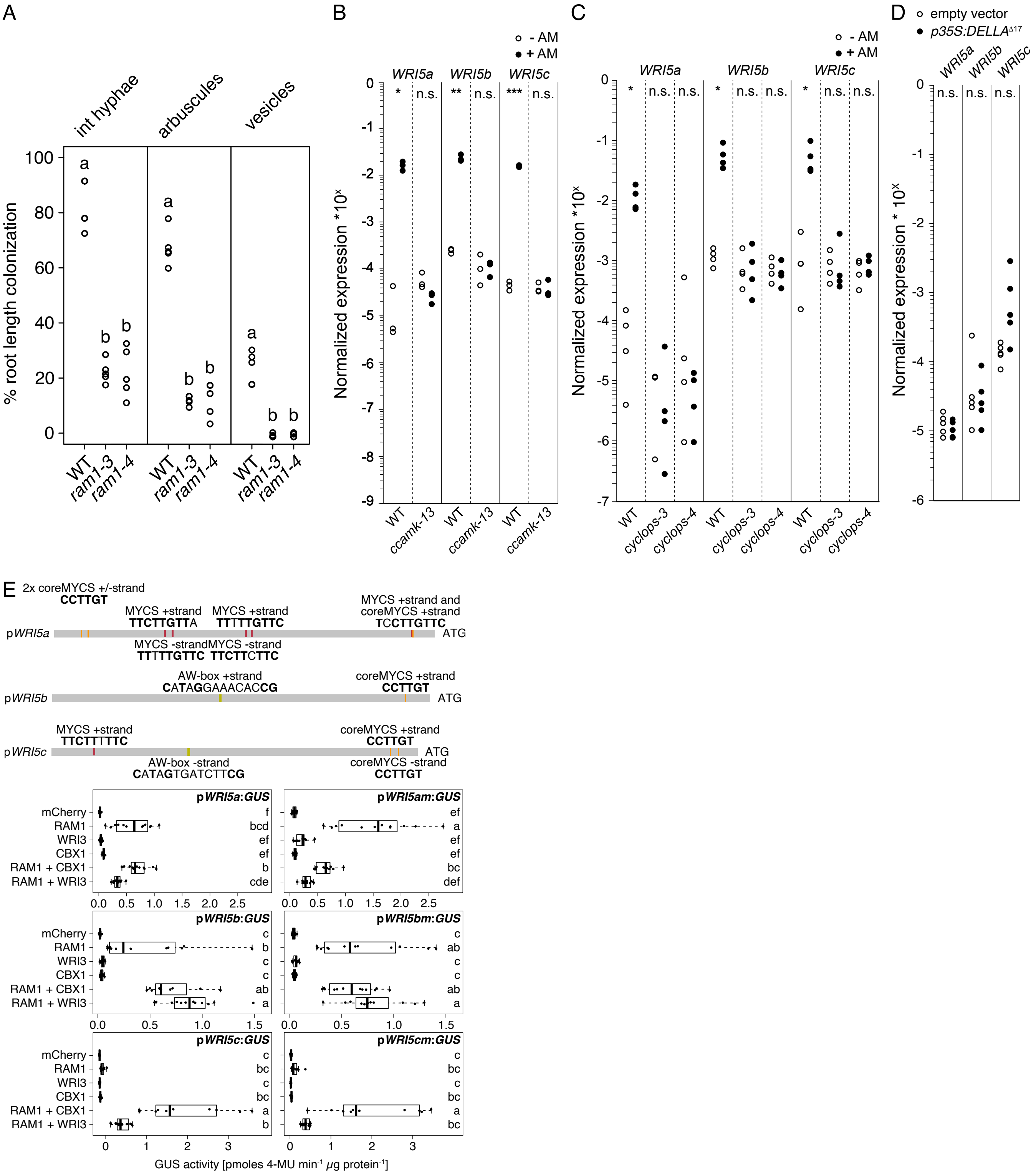
A
int hyphae
arbuscules
vesicles
% root length colonization
WT
ram1-3
ram1-4
B
- AM
+ AM
WRI5a
WRI5b
WRI5c
n.s.
Normalized expression *10x
ccamk-13
C
cyclops-3
cyclops-4
D
empty vector
p35S:DELLAΔ17
Normalized expression * 10x
E
2x coreMYCS +/-strand
CCTTGT
MYCS +strand
TTCTTGTTA
MYCS +strand
TTTTTGTTC
MYCS +strand and
coreMYCS +strand
TCCTTGTTC
pWRI5a
ATG
MYCS -strand
TTTTTGTTC
MYCS -strand
TTCTTCTTC
AW-box +strand
CATAGGAAACACCG
coreMYCS +strand
CCTTGT
pWRI5b
MYCS +strand
TTCTTTTTC
coreMYCS +strand
CCTTGT
pWRI5c
AW-box -strand
CATAGTGATCTTCG
coreMYCS -strand
CCTTGT
pWRI5a:GUS
pWRI5am:GUS
pWRI5b:GUS
pWRI5bm:GUS
pWRI5c:GUS
pWRI5cm:GUS
mCherry
RAM1
WRI3
CBX1
RAM1 + CBX1
RAM1 + WRI3
GUS activity [pmoles 4-MU min-1 µg protein-1]

Fig. S14 Expression of *WRI5a*, *b* and *c* is not induced by DELLA*. (A)* Root length colonization of wild-type, *ram1-3* and *ram1-4* roots, colonized with *R. irregularis* at 5 wpi, corresponding to RT-qPCR data in Fig. 6*C* and S13*H*. Different letters indicate different statistical groups (ANOVA; post hoc Tukey; n=8 except WT n=6; p < 0.05). (*B*) Transcript accumulation of *WRI5a*, *WRI5b* and *WRI5c* in wild-type and *ccamk-13* roots colonized by *R. irregularis* at 6 wpi, corresponding root colonization data are displayed in Fig. S13*A*. Statistical analysis: Welch t-test (n=3, * p < 0.05, ** p < 0,01, *** p < 0,001*)*. (*C*) Transcript accumulation of *WRI5a*, *WRI5b* and *WRI5c* in wild-type, *cyclops-3* and *cyclops-4* roots colonized by *R. irregularis* at 6 wpi. Corresponding root colonization data are displayed in Fig. S13*B*. Statistical analysis: Welch t-test (n=4, * p < 0.05). (*D*) Transcript accumulation of *WRI5a, WRI5b* and *WRI5c* in wild-type hairy roots transformed with an empty vector (EV) or a p*35S*:$^{\Delta 17}$*DELLA1* expression cassette at 5 wpp. Statistical analysis: Welch t-test (n=5*)*. (*E*) Transactivation assay in *N. benthamiana* leaves. The indicated promoter-reporter plasmids were co-transformed with plasmids containing the genomic sequence encoding the proteins indicated at the y-axis driven by the *LjUbiquitin10* promoter. The schematic representation on top illustrates the position and sequence of the *AW*-boxes (yellow marks), *MYCS*-elements (red marks) and *cMYCS*-elements (orange marks) in all three promoters*.* Bold letters indicate the base pairs, defining the respective motif. Bold black line, median; box, interquartile range; whiskers highest and lowest data point within 1.5 interquartile range; dots, actual values. Different letters indicate different statistical groups (ANOVA; post hoc Tukey; n=12, p < 0.05). (*B-D*) Transcript accumulation was determined by RT-qPCR, and expression of the housekeeping gene *Ubiquitin10* was used for normalization. All experiments were performed twice with similar results except for (*D*) and (*E*), which were performed once.

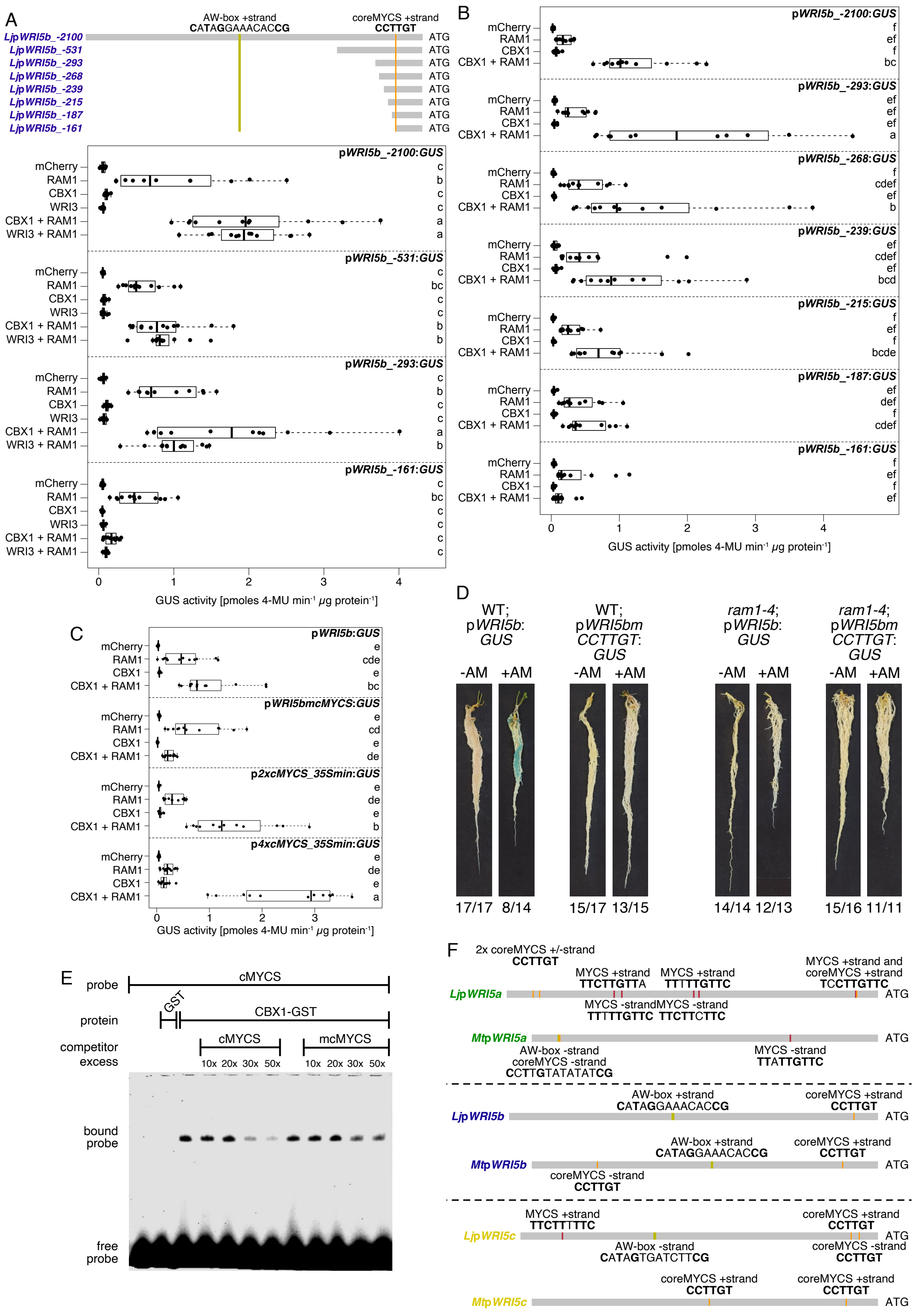
A
AW-box +strand
CATAGGAAACACCG
coreMYCS +strand
CCTTGT
LjpWRI5b_-2100
LjpWRI5b_-531
LjpWRI5b_-293
LjpWRI5b_-268
LjpWRI5b_-239
LjpWRI5b_-215
LjpWRI5b_-187
LjpWRI5b_-161
ATG
pWRI5b_-2100:GUS
pWRI5b_-531:GUS
pWRI5b_-293:GUS
pWRI5b_-161:GUS
mCherry
RAM1
CBX1
WRI3
CBX1 + RAM1
WRI3 + RAM1
GUS activity [pmoles 4-MU min-1 μg protein-1]
B
pWRI5b_-268:GUS
pWRI5b_-239:GUS
pWRI5b_-215:GUS
pWRI5b_-187:GUS
C
pWRI5b:GUS
pWRI5bmcMYCS:GUS
p2xcMYCS_35Smin:GUS
p4xcMYCS_35Smin:GUS
D
WT; pWRI5b: GUS
WT; pWRI5bm CCTTGT: GUS
ram1-4; pWRI5b: GUS
ram1-4; pWRI5bm CCTTGT: GUS
-AM
+AM
17/17
8/14
15/17
13/15
14/14
12/13
15/16
11/11
E
probe
cMYCS
protein
GST
CBX1-GST
competitor excess
mcMYCS
10x
20x
30x
50x
bound probe
free probe
F
2x coreMYCS +/-strand
CCTTGT
MYCS +strand
TTCTTGTTA
TTTTTGTTC
MYCS +strand and coreMYCS +strand
TCCTTGTTC
LjpWRI5a
MYCS -strand
TTTTTGTTC
TTCTTCTTC
MtpWRI5a
AW-box -strand coreMYCS -strand
CCTTGTATATATCG
MYCS -strand
TTATTGTTC
LjpWRI5b
MtpWRI5b
AW-box +strand
CATAGGAAACACCG
coreMYCS -strand
TTCTTTTTC
LjpWRI5c
AW-box -strand
CATAGTGATCTTCG
MtpWRI5c

Fig. S15 Importance of the cMYCS element in the *WRI5b* promoter. (*A, B*) Transactivation assay in *N. benthamiana* leaves. The *WRI5b* promoter fragment in the p*WRI5b_-2100*:*GUS* reporter plasmid corresponds to the same promoter fragment that was used in Fig. S14*E*. The numbers of the constructs correspond to the number of base pairs upstream of the TSS still included in the respective promoter fragment. The schematic representation on the top left shows the position of cis-regulatory elements and their position in the promoter fragments. Reporter plasmids were co-transformed with plasmids containing the genomic sequence of the proteins indicated at the y axis driven by the constitutive *LjUbiquitin10* promoter. (*C*) Transactivation assay in *N. benthamiana* leaves. The reporter plasmids containing p*WRI5b* or p*WRI5bmMYCS*:*GUS* and multiple tandem copies of cMYCS fused with the *35S* minimal promoter and *GUS* were co-transformed with plasmids containing the genomic sequence encoding the proteins indicated at the y-axis driven by the *LjUbiquitin10* promoter. Bold letters indicate the base pairs, defining the respective motif**.** (*A-C*) Bold black line, median; box, interquartile range; whiskers highest and lowest data point within 1.5 interquartile range; dots, actual values. Different letters indicate different statistical groups (ANOVA; post hoc Tukey; n=12; $p < 0.05$). *(D)* Representative images of GUS activity resulting from p*WRI5b* and p*WRI5bmCCTTGT* activation in wild-type and *ram1-4* hairy roots colonized with *R. irregularis* at 6 wpi. Roots were stained with X-Gluc for 6h. The numbers indicate the number of root systems that displayed a staining as shown in the image, among the total number of analyzed root systems*. (E)* Image of a polyacrylamid gel loaded with mixtures of the indicated proteins and Cy5-labelled probes for electromobility shift assay (EMSA). Cold competitor and mutated cold competitor probes were added in increasing excess compared to the labelled probe, from left to right. R =3 *(F)* Schematic representation of the location of *AW*-boxes, *MYCS*- and *cMYCS*-elements in the *WRI5a*, *b* and *c* promoters of *L. japonicus* (*Lj*) and *M. truncatula* (*Mt*). Except for (*E*) which was performed three times with similar results, the experiments were performed once.

Supplementary Tables

Table S1. Numbers of root cortex cells containing arbuscules with mCitrine signal indicating protein-protein interaction in BiFC as shown in Figure 2C and S9D. Ten individual roots per plant were picked and screened on a fluorescence stereomicroscope for cells colonized by arbuscules, as indicated by arbuscle-shaped mCherry fluorescence. The root pieces with arbuscules were then subjected to confocal imaging to screen for mCitrine signal. The numbers of cells with and without mCitrine signal were determined by counting cells on micrographs obtained at the confocal microscope. mCitrine signal observed only in cells with developing arbuscules is shown in violet, and mCitrine signal observed only in cells with mature arbuscules is shown in blue.

Interaction	Independent experiment	Number of cells with mCitrine signal	Number of cells without mCitrine signal	Number of root systems screened	Number of individual roots per root system screened
CBX1-RAM1	1	-	68	3	10
	2	10	58	3	10
	3	2	151	3	10
CBX1M4-RAM1	1	-	17	3	10
	2	-	52	3	10
CBX1M7-RAM1	1	-	8	2	10
	2	-	35	2	10
	3	-	112	3	10
CBX1-RAM1M3	2	-	28	3	10
	3	-	154	3	10
WRI5b-RAM1	1	-	17	3	10
	2	-	19	3	10
	3	2	487	3	10
WRI5b-RAM1M3	2	-	23	3	10
	3	-	64	3	10

Table S2. Primers for RT-qPCR

gene	forward primer (5'->3')	reverse primer (5'->3')
Ubiquitin10	ATGCAGATCTTCGTCAAGACCTTG	ACCTCCCCTCAGACGAAG
RAM1	TGCATTGAATCATGCTACGTT	CCTTGTGGAGACCATCCATT
RAM2	ATCCTATGAGTGCACTAGCTTTACTAGAAG	AACGAGCAAATTAAAACTGAAAGAGAGTAC
STR	CTATATTGGTGACGAGGGAAGG	GTCCTGAGGTAGGTTCATCCAG
PT4		
WRI3	CAAAGTCAAACCCTGCTTCTCG	GGTGCAGTAATTGAGCTCAGC
CBX1	CAGCTGAGTATGATATTCAG	CTTCAAAGTCATCACAATGG
WRI5a	CACTCAACAAGGCAATGATGGG	TGTTGTTTCTTCTGGGGACTCC
WRI5b	GCCAATCCTTCCAATTCTCCAGC	GCCATCATAGAAGATTCCACCCAGTTC
WRI5c	CGCATGCAGCTAGTGATGAT	CCTTGACTTTGAGGGCTGAC

Table S3. Probes for EMSA

Oligonucleotide	sequence (5'->3')
Cy5-*cMYCS*-F	[Cy5]-TTTCCCTTGTTTGC
cMYCS-F	TTTCCCTTGTTTGC
cMYCS-R	GCAAACAAGGGAAA
mutated-*cMYCS*-F	TTTCATCGTGTTGC
mutated-*cMYCS*-R	GCAACACGATGAAA

Table S4. Primers for cloning.

purpose	name	Sequence (5'->3')
cloning *WRI3* and mutate type IIs restriction sites	MP277<br>MP278<br>MP279<br>MP280<br>MP281<br>MP282	ATGAAGACTTTACGGGTCTCACACCATGGCTAAGAAGAAAGGCC<br>ATGAAGACTTCCTCCCTTTCTTGCTCTG<br>ATGAAGACTTGAGGCAAGGTTCTTAATTAATACTTAGATC<br>ATGAAGACTTAACAAGCCCAAGTTCAAGTTC<br>ATGAAGACTTTGTTTCACATCAAATTGGCCAGAC<br>ATGAAGACTTCAGAGGTCTCACCTTAGCATTGAGCTCATAATGAAAAATGGGTG
cloning *CBX1*	MP276a<br>MP276b	ATGAAGACTTTACGGGTCTCACACCATGGGGAAACTCTCACAGCAC<br>ATGAAGACTTCAGAGGTCTCACCTTAACCTTCAAGCCTTCAAAGTCATCAC
cloning *WRI5a* and mutate type IIs restriction sites	MP258<br>MP259<br>MP260<br>MP261	ATGAAGACTTTACGGGTCTCACACCATGGAGTTTGCTTCTGTAAAATCTG<br>ATGAAGACTTTAATACACTGTTGTAATCAAACACTTGAAG<br>ATGAAGACTTATTATATAGATAACTTGATATAAAGAAACTGACATC<br>ATGAAGACTTCAGAGGTCTCACCTTTTCCATAGGGGGAAACATG
cloning *WRI5b* and mutate type IIs restriction sites	MP262<br>MP263<br>MP264<br>MP265<br>MP266<br>MP267	ATGAAGACTTTACGGGTCTCACACCATGGCAATGATGACAGAAAATGAAGTTATC<br>ATGAAGACTTGTCGCTTGACAGATTTTGCTC<br>ATGAAGACTTCGACGTAAGAGAGAGCCTG<br>ATGAAGACTTAACACCTCTTGAAAAGCCACTG<br>ATGAAGACTTTGTTTCAAAGTATAGGGGTGTTG<br>ATGAAGACTTCAGAGGTCTCACCTTGAATATTGAATTAAGCTCTCTTTCTTGC
cloning *WRI5c* and mutate type IIs restriction sites	MP268<br>MP269<br>MP270<br>MP271<br>MP272<br>MP273<br>MP274<br>MP275	ATGAAGACTTTACGGGTCTCACACCATGGAAATAGTGATAAAGCATGAAGAGAACAAGAG<br>ATGAAGACTTCCTTTTGGCACCCGGAG<br>ATGAAGACTTAAGGCGGCGAAGAGAGC<br>ATGAAGACTTTAATACAGGATCCACAAATGAATGAGAAAAAGTAAGCAAC<br>ATGAAGACTTATTATCTTGGAAAGCATTTGTTTATCCTTTCTAACATAG<br>ATGAAGACTTATCCTCCCCATCAGTTTCATCC<br>ATGAAGACTTGGATACAAAAGATCAACAGCCGC<br>ATGAAGACTTCAGAGGTCTCACCTTGCAATGGTAGTGTGGATTATCTAGCAC
cloning c*WRI3.1* and mutate type IIs restriction sites	MP277<br>MP278<br>MP279<br>MP282	ATGAAGACTTTACGGGTCTCACACCATGGCTAAGAAGAAAGGCC<br>ATGAAGACTTCCTCCCTTTCTTGCTCTG<br>ATGAAGACTTGAGGCAAGGTTCTTAATTAATACTTAGATC<br>ATGAAGACTTCAGAGGTCTCACCTTAGCATTGAGCTCATAATGAAAAATGGGTG
cloning c*WRI3.2* and mutate type IIs restriction sites	MP277<br>MP278<br>MP279<br>MP282	ATGAAGACTTTACGGGTCTCACACCATGGCTAAGAAGAAAGGCC<br>ATGAAGACTTCCTCCCTTTCTTGCTCTG<br>ATGAAGACTTGAGGCAAGGTTCTTAATTAATACTTAGATC<br>ATGAAGACTTCAGAGGTCTCACCTTAGCATTGAGCTCATAATGAAAAATGGGTG

cloning c*CBX1*	MP276a<br>MP276b	ATGAAGACTTTACGGGTCTCACACCATGGGGAAACTCTCACAGCAC<br>ATGAAGACTTCAGAGGTCTCACCTTAACCTTCAAGCCTTCAAAGTCATCAC
cloning c*WRI5a*	MP258<br>MP261	ATGAAGACTTTACGGGTCTCACACCATGGAGTTTGCTTCTGTAAAATCTG<br>ATGAAGACTTCAGAGGTCTCACCTTTTCCATAGGGGGAAACATG
cloning c*WRI5b* and mutate type IIs restriction sites	MP262<br>MP263<br>MP264<br>MP265<br>MP266<br>MP267	ATGAAGACTTTACGGGTCTCACACCATGGCAATGATGACAGAAAATGAAGTTATC<br>ATGAAGACTTGTCGCTTGACAGATTTTGCTC<br>ATGAAGACTTCGACGTAAGAGAGAGCCTG<br>ATGAAGACTTAACACCTCTTGAAAAGCCACTG<br>ATGAAGACTTTGTTTCAAAGTATAGGGGTGTTG<br>ATGAAGACTTCAGAGGTCTCACCTTGAATATTGAATTAAGCTCTCTTTCTTGC
cloning c*WRI5c* and mutate type IIs restriction sites	MP268<br>MP269<br>MP270<br>MP273<br>MP274<br>MP275	ATGAAGACTTTACGGGTCTCACACCATGGAAATAGTGATAAAGCATGAAGAGAACAAGAG<br>ATGAAGACTTCCTTTTGGCACCCGGAG<br>ATGAAGACTTAAGGCGGCGAAGAGAGC<br>ATGAAGACTTATCCTCCCCATCAGTTTCATCC<br>ATGAAGACTTGGATACAAAAGATCAACAGCCGC<br>ATGAAGACTTCAGAGGTCTCACCTTGCAATGGTAGTGTGGATTATCTAGCAC
cloning p*WRI3*	MP303<br>MP304	TTTCGTCTCAGCGGGCTGTGATGAAGATTCGATAGTAGATGAGG<br>TTTCGTCTCACAGAGAATTGAATTGAATGTTAATGCTGAGTCATAACAAGAGAAG
cloning p*CBX1*	MP301<br>MP302	TTTCGTCTCAGCGGCCACACTCTCTTCAAACATATGATTAGTGACAG<br>TTTCGTCTCACAGAAATGTTGCACCTCTAGGATTTGATGTAAC
cloning p*WRI5a*	MP293<br>MP294	TTTCGTCTCAGCGGTCGATGCACTGTTGGAAAGCC<br>TTTCGTCTCACAGATATACACTTGTATTACTCACCATGCAAGCAAG
cloning p*WRI5b*	MP406<br>MP407<br>MP408<br>MP409	TTTCGTCTCACAGATCTTTAATTGTGATTTTCTGTGTCTTCTTTTCCTTCTC<br>ATGAAGACTTCTAAAAAAATTGTATTAATCTTATTTATTATTATTTTATAATTTAACATGATAAACTAATAATATAG<br>ATGAAGACTTTTAGGATAATAATAATACAAGTTGAATGAATATCATATGAGC<br>ATGAAGACTTCAGAGGTCTCACAGATTCTTGAGTTTTGATGTTCTCACTGTTAG
cloning p*WRI5c*	MP299<br>MP300	TTTCGTCTCAGCGGGTACAGGTTGGTTCATAATTGCATCACAAC<br>TTTCGTCTCACAGATCTTTAATTGTGATTTTCTGTGTCTTCTTTTCCTTCTC
cloning p*CBX1m*	MP301<br>MP330<br>MP329<br>MP302	TTTCGTCTCAGCGGCCACACTCTCTTCAAACATATGATTAGTGACAG<br>TTTCGTCTCAATTATGAAGGGTTGTGGTATTATGTGGCC<br>TTTCGTCTCATAATTATGATCTCATGTGTAATTGCCAGCAC<br>TTTCGTCTCACAGAAATGTTGCACCTCTAGGATTTGATGTAAC

cloning p*WRI3m*	AL76	ATGGTCTCTCTTTCTTTAATTAATTACATGAATGAGACCAT
	AL77	ATGGTCTCATTCATGTAATTAATTAAAGAAAGAGAGACCAT
	AL78	ATGGTCTCTCACCCGTCTCAGCGGGCTGTGATGAA
	AL79	ATGGTCTCGAAAGGATGGTTTTGATTGTGGCCGTCTTTA
	AL80	ATGGTCTCTTGAATCTTTAAGTCCCAAACACTCTGCTC
	AL81	ATGGTCTCCCCTTTCGTCTCACAGAGAATTGAATTGAATGTTAA
cloning p*WRI5am*	MP400	ATGAAGACTTTACGGGTCTCAGCGGTCGATGCACTGTTGGAAAGCC
	MP479	TTGGTCTCAACTGTAATTCCTATGAGGGGGCCACCTCCAGCTAGCATTC
	MP480	TTGGTCTCACAGTTCTTGAAACGTGAGTCCACCCCCTTCTTTG
	MP481	TTGGTCTCAATGTGTTATTTGGAGGAGGAATAGTTTCTAATAAG
	MP482	TTGGTCTCAACATTTTATTGCACGGGGGTGGAGAAAAAAATATGTC
	KH104	TTGGTCTCATCCAATAAAATTGTCGATGTTATCATCACCATGAGGCG
	KH105	TTGGTCTCATGGAAGGTGGACAGAACACAGTTTTCAAGTACAAAAGTACAATATAAGC
	MP405	ATGAAGACTTCAGAGGTCTCACAGATATACACTTGTATTACTCACCATGCAAGCAAG
cloning p*WRI5bm*	MP406	ATGAAGACTTTACGGGTCTCAGCGGCCTACATAGCCCTACTTTACTACTTCCTCC
	MP483	TTGGTCTCAGTTTCATCTTATCAAAAGCTCATATGATATTCATTCAACTTG
	MP484	TTGGTCTCAAAACACATTAAATATTATATAACCGTGGGACAATCAAG
	MP409	ATGAAGACTTCAGAGGTCTCACAGATTCTTGAGTTTTGATGTTCTCACTGTTAG
cloning p*WRI5cm*	MP410	ATGAAGACTTTACGGGTCTCAGCGGGTACAGGTTGGTTCATAATTGCATCACAAC
	MP537	TTGGTCTCAAAAAGAACATGGTTTTTCTCTTTAAGCTTGTAAGC
	MP538	TTGGTCTCATTTTTCTTCTTCTTTTTATATACTCTGCAGATTG
	MP487	TTGGTCTCATGATCTTATCATAGGGGTTATAAAATGTTGATTTC
	MP488	TTGGTCTCAATCAATCTTACAATTTTGTTCAAAGAATAATTAGAGATAAATTCC
	MP411	ATGAAGACTTCAGAGGTCTCACAGATCTTTAATTGTGATTTTCTGTGTATTATTTTCCTTCTCAACAAGTG
cloning p*WRI5b_-531*	KH158	TTTGGTCTCAGCGGTGGCTAATACATAGACGAATTTAGATTCAGC
	MP409	ATGAAGACTTCAGAGGTCTCACAGATTCTTGAGTTTTGATGTTCTCACTGTTAG
cloning p*WRI5b_-293*	KH159	TTTGGTCTCAGCGGGGGTGCTTCTTATGGGTAAGGGAAG
	MP409	ATGAAGACTTCAGAGGTCTCACAGATTCTTGAGTTTTGATGTTCTCACTGTTAG

cloning p*WRI5b_-161*	KH160<br><br>MP409	TTTGGTCTCAGCGGGCAACTCCAATCATGTCACTAACAATAT G<br>ATGAAGACTTCAGAGGTCTCACAGATTCTTGAGTTTTGATGTT CTCACTGTTAG
cloning p*WRI5b_-268*	KH161<br><br>MP409	TTTGGTCTCAGCGGGTTGGAATTCTCACGAAGCAAATTCAAA AC<br>ATGAAGACTTCAGAGGTCTCACAGATTCTTGAGTTTTGATGTT CTCACTGTTAG
cloning p*WRI5b_-239*	KH162<br>MP409	TTTGGTCTCAGCGGCTTTTGGACTTTGAGCTGTCAATAGGAC<br>ATGAAGACTTCAGAGGTCTCACAGATTCTTGAGTTTTGATGTT CTCACTGTTAG
cloning p*WRI5b_-215*	KH163<br><br>MP409	TTTGGTCTCAGCGGGGACAACACAAAACCTGTCATCTAATTT CC<br>ATGAAGACTTCAGAGGTCTCACAGATTCTTGAGTTTTGATGTT CTCACTGTTAG
cloning p*WRI5b_-187*	KH164<br><br>MP409	TTTGGTCTCAGCGGCCCTGTAATTATTGTTTCCCTTGTTTGCA AC<br>ATGAAGACTTCAGAGGTCTCACAGATTCTTGAGTTTTGATGTT CTCACTGTTAG
cloning *pWRI5bmCCTTGT*	MP406<br><br>KH186<br><br>KH187<br><br>MP409	ATGAAGACTTTACGGGTCTCAGCGGCCTACATAGCCCTACTT TACTACTTCCTCC<br>TAGGTCTCTCGATGAAACAATAATTACAGGGAAATTAGATGA CAGG<br>ATGGTCTCAATCGTGTTGCAACTCCAATCATGTCACTAACAAT ATGTAC<br>ATGAAGACTTCAGAGGTCTCACAGATTCTTGAGTTTTGATGTT CTCACTGTTAG
cloning *2xCCTTGT*	KH190<br>KH191	ATGAAGACTTTACGGGTCTCAGCGGAGTGCCCTTGTCATCA<br>ATGAAGACTTCAGAGGTCTCTCAGAACAAGGTGATGACAAGG GCACT
cloning *4xCCTTGT*	KH188<br><br>KH189	ATGAAGACTTTACGGGTCTCAGCGGCCTTGTTAATCCCTTGT AGTGCCCTTGTCATCA<br>ATGAAGACTTCAGAGGTCTCTCAGAACAAGGTGATGACAAGG GCACTAC
cloning p*2xCCTTGT_35S min*	KH192<br>KH199	ATGGTCTCAGCGGAGTGCCCTTGTCATCACCTTGTTC<br>TAGGTCTCTCAGATGGGCTGTCCTCTCCAAATGAAATG
cloning p*4xCCTTGT_35S min*	KH194<br>KH199	ATGGTCTCAGCGGCCTTGTTAATCCCTTGTAGTGCC<br>TAGGTCTCTCAGATGGGCTGTCCTCTCCAAATGAAATG
cloning p*RAM2m*	MP420<br><br>MP421<br>MP422<br>MP423<br><br>MP424<br>MP425<br>MP426<br>MP427<br>MP428<br>MP429	ATGAAGACTTTACGGGTCTCAGCGGCTGATTGAAAGCTTCCC CATAGG<br>ATGAAGACTTCTTGCCACCTTTGGTATACTGG<br>ATGAAGACTTCAAGCATGCCAAACATCTCATTG<br>ATGAAGACTTGTGGACTATTTAACTGTCATTTTGATGTTTTAA G<br>ATGAAGACTTCCACCCCCATTCTTCTAAAACG<br>ATGAAGACTTGCATGAATCATCTACATCAGCAAAAAC<br>ATGAAGACTTATGCCACCATAAAAAAGCATTGC<br>ATGAAGACTTATGTAAAAGCGTATTGGCCATATCC<br>ATGAAGACTTACATATTTCAGTATCCGCCAATCAC<br>ATGAAGACTTCAGAGGTCTCACAGAGGTGAATGCACTTGTTG TTACTCTAATAAC

cloning p*RAM2mAW*	MP420	ATGAAGACTTTACGGGTCTCAGCGGCTGATTGAAAGCTTCCCCATAGG
	MP421	ATGAAGACTTCTTGCCACCTTTGGTATACTGG
	MP422	ATGAAGACTTCAAGCATGCCAAACATCTCATTG
	MP425	ATGAAGACTTGCATGAATCATCTACATCAGCAAAAAC
	MP426	ATGAAGACTTATGCCACCATAAAAAAGCATTGC
	MP427	ATGAAGACTTATGTAAAAGCGTATTGGCCATATCC
	MP428	ATGAAGACTTACATATTTCAGTATCCGCCAATCAC
	MP429	ATGAAGACTTCAGAGGTCTCACAGAGGTGAATGCACTTGTTGTTACTCTAATAAC
cloning p*RAM2mMYCS*	MP420	ATGAAGACTTTACGGGTCTCAGCGGCTGATTGAAAGCTTCCCCATAGG
	MP423	ATGAAGACTTGTGGACTATTTAACTGTCATTTTGATGTTTTAAG
	MP424	ATGAAGACTTCCACCCCCATTCTTCTAAAACG
	MP429	ATGAAGACTTCAGAGGTCTCACAGAGGTGAATGCACTTGTTGTTACTCTAATAAC
cloning p*PT4m*	MP458	ATGAAGACTTTACGGGTCTCAGCGGGCGGGGACTCAAGAAACCATG
	MP459	TTGAAGACATGCCTAGCAACTTCTAAACTTTGATGATTTCTC
	MP460	ATGAAGACTTAGGCATACGCTAACTTATGTG
	MP461	ATGAAGACTTATAGTATACCCTATGTAGGGTTG
	MP462	ATGAAGACTTCTATAGATCGTGACAATAATGAGCATATTTTAGCTCTATATCTTTCTCATGGGGTAG
	MP463	ATGAAGACTTCACCTCCGGAGATGTTCAACCTCCATAGAATTCTCTAGC
	MP464	ATGAAGACTTGGTGGAACATCTCCTAAAATGATGACTACAATTCCATTTC
	MP465	ATGAAGACTTCAGAGGTCTCACAGACTTGAACGATGTCGATTTAGTTTGTTTCTATGTTGTGTGGG
cloning p*PT4mAW*	MP458	ATGAAGACTTTACGGGTCTCAGCGGGCGGGGACTCAAGAAACCATG
	MP459	TTGAAGACATGCCTAGCAACTTCTAAACTTTGATGATTTCTC
	MP460	ATGAAGACTTAGGCATACGCTAACTTATGTG
	MP461	ATGAAGACTTATAGTATACCCTATGTAGGGTTG
	MP462	ATGAAGACTTCTATAGATCGTGACAATAATGAGCATATTTTAGCTCTATATCTTTCTCATGGGGTAG
	MP475	ATGAAGACTTTCATCATTTTAGGAGATGTGAACAAGAAGGAGATGTGACC
	MP476	ATGAAGACTTATGACTACAATTCCATTTCTGTGTTACCAACTTCTTTAATAATG
	MP465	ATGAAGACTTCAGAGGTCTCACAGACTTGAACGATGTCGATTTAGTTTGTTTCTATGTTGTGTGGG
cloning p*PT4mMYCS*	MP458	ATGAAGACTTTACGGGTCTCAGCGGGCGGGGACTCAAGAAACCATG
	AL35	ATGAAGACTTTTTCTCTGCCTTTTCGTCCTTATAAGCC
	AL36	ATGAAGACTTGAAATCATCAAAGTTTAGCATTGGCTA
	MP463	ATGAAGACTTCACCTCCGGAGATGTTCAACCTCCATAGAATTCTCTAGC
	MP464	ATGAAGACTTGGTGGAACATCTCCTAAAATGATGACTACAATTCCATTTC
	MP465	ATGAAGACTTCAGAGGTCTCACAGACTTGAACGATGTCGATTTAGTTTGTTTCTATGTTGTGTGGG

cloning p*STR*	MP452<br><br>MP470	ATGAAGACTTTACGGGTCTCAGCGGCTCAAGTGGTAAGAGCT AGGAAC<br>ATGAAGACTTCAGAGGTCTCACAGATGCAGTTAACTTAACCA AAGCAAC
cloning p*STRm*	MP452<br><br>MP453<br>MP454<br>MP455<br>MP456<br><br>MP468<br><br>MP469<br>MP470	ATGAAGACTTTACGGGTCTCAGCGGCTCAAGTGGTAAGAGCT AGGAAC<br>ATGAAGACTTGGGTGGAATATCTCTCTTTTTTTCCTATC<br>ATGAAGACTTACCCCCACAAGTGAAAATGAG<br>ATGAAGACTTTTGTGCCGAAGAATGACATTG<br>ATGAAGACTTACAATCAAAGCAAATTTAACGACAAACCCAAAG GGAAACTGAAATGG<br>ATGAAGACTTTAATACAATTGAAAGAGAAACAAAACATGAGAC GGCAAACTTAGGGGGAGGGGGGAGTCCATTTCAGTTTC<br>ATGAAGACTTATTACCCCAATATTTACAAATCACAACC<br>ATGAAGACTTCAGAGGTCTCACAGATGCAGTTAACTTAACCA AAGCAAC
cloning *STR* and mutate type IIs restriction sites	CC66<br>CC67<br><br>CC69<br>CC70<br>CC71<br><br>CC72<br>CC73<br>CC74<br><br>CC75<br>CC76<br>CC77<br>CC155	GATGATGGAGAGTAGACAGAAAC<br>AGTGCTGAAGACTGTACGGGTCTCACACCGCCAGGGTAGAC AGGACA<br>AGTGCTGAAGACCTAACACCTATTTACCACCTCTG<br>AGTGCTGAAGACCTGGATACGAGTCCCATAGCATACT<br>AGTGCTGAAGACGTATCCTGCATAAGAATAGCCCCTATGCCA ACTCCG<br>AGTGCTGAAGACGTGAAGGACACCCGCGGGCACGG<br>AGTGCTGAAGACGACTTCTCTACAGCTCGGGTC<br>AAGAAGACAACAGAGGTCTCACCTTTTCATGTAGCTTGTAGA TACC<br>AGTGCTGAAGACGAGGTGTCATCATATGTGATGC<br>AGTGCTGAAGACGAAGTCTACCCCTATAAAACC<br>AGTGCTGAAGACTCGACTGATATACATGGAAAGCCA<br>AGTGCTGAAGACTCCAGAGGTCTCACCTTTTTTCTCTCAATTT TGGAA
mutate type IIs restriction site in the amplicon of CC69 + CC70 by overlapping PCR	CC69<br>CC58<br>CC57<br>CC70	AGTGCTGAAGACCTAACACCTATTTACCACCTCTG<br>ACACTGGAGATCCATATTTGATAG<br>CTATCAAATATGGATCTCCAGTGT<br>AGTGCTGAAGACCTGGATACGAGTCCCATAGCATACT
mutate type IIs restriction site in the amplicon of CC71 + CC72 by overlapping PCR	CC71<br><br>CC60<br>CC59<br>CC62<br>CC61<br>CC64<br>CC63<br>CC66<br>CC65<br>CC72	AGTGCTGAAGACGTATCCTGCATAAGAATAGCCCCTATGCCA ACTCCG<br>CAGTGAACGTGTGACCATATAG<br>CTATATGGTCACACGTTCACTG<br>TTCAAGGAACCAACTAGACTTC<br>GAAGTCTAGTTGGTTCCTTGAA<br>GGATTCCAGCGAGCAAGAC<br>GTCTTGCTCGCTGGAATCC<br>GATGATGGAGAGTAGACAGAAAC<br>GTTTCTGTCTACTCTCCATCATC<br>AGTGCTGAAGACGTGAAGGACACCCGCGGGCACGG
cloning *cCBX1_N*	MP276a<br><br>MP474	ATGAAGACTTTACGGGTCTCACACCATGGGGAAACTCTCACA GCAC<br>TTTGGTCTCACCTTAATTGGAGAATCTCTAGGGACACTTCTC
cloning *cCBX1_C*	MP47<br>MP276b	TTTGGTCTCACACCAAGTGGCTTAAGCCTAATCAAAGC<br>ATGAAGACTTCAGAGGTCTCACCTTAACCTTCAAGCCTTCAA AGTCATCAC

cloning *cCBX1_AP2*	MP471<br>MP472	TTTGGTCTCACACCCAAAGAAGCTCAATATACAGAGGAGTC<br>TTTGGTCTCACCTTAATGTAACGGCTTAGGTCAAAATTGG
cloning *cCBX1_N + AP2*	MP276a<br><br>MP472	ATGAAGACTTTACGGGTCTCACACCATGGGGAAACTCTCACA GCAC<br>TTTGGTCTCACCTTAATGTAACGGCTTAGGTCAAAATTGG
cloning *cCBX1_C + AP2*	MP471<br>MP276b	TTTGGTCTCACACCCAAAGAAGCTCAATATACAGAGGAGTC<br>ATGAAGACTTCAGAGGTCTCACCTTAACCTTCAAGCCTTCAA AGTCATCAC
cloning *cCBX1_CΔ1*	AL24<br><br>MP276b	ATGAAGACttTACGGGTCTCaCACCTCCCAGATAACCCTCATG ATAAAA<br>ATGAAGACTTCAGAGGTCTCACCTTAACCTTCAAGCCTTCAA AGTCATCAC
cloning *cCBX1_CΔ2*	AL26<br>MP276b	ATGAAGACttTACGGGTCTCaCACCTCCAGCCTAGGCCAGCT<br>ATGAAGACTTCAGAGGTCTCACCTTAACCTTCAAGCCTTCAA AGTCATCAC
cloning *cCBX1_CΔ3*	AL28<br><br>MP276b	ATGAAGACttTACGGGTCTCaCACCTGTCAACGCCAGCTGAGT AT<br>ATGAAGACTTCAGAGGTCTCACCTTAACCTTCAAGCCTTCAA AGTCATCAC
cloning *cCBX1_CΔ4*	AL30<br><br>MP276b	ATGAAGACttTACGGGTCTCaCACCGAGAAGGGGATGATGATA TGAATA<br>ATGAAGACTTCAGAGGTCTCACCTTAACCTTCAAGCCTTCAA AGTCATCAC
cloning *RAM1-M5 ΔN1*	MP227<br>MP226	TTTGGTCTCACACCCCACAAAGCATCTCCAACGGC<br>TTTGGTCTCACCTTTCACTTGCATCTCCATGCAGAGGCAG
cloning *RAM1-M5 ΔN2*	MP228<br>MP226	TTTGGTCTCACACCCCGTGCATCGACTCGGTC<br>TTTGGTCTCACCTTTCACTTGCATCTCCATGCAGAGGCAG
cloning *RAM1-M5 ΔN3*	MP229<br>MP226	TTTGGTCTCACACCGTCGGGGAGGCACTGG<br>TTTGGTCTCACCTTTCACTTGCATCTCCATGCAGAGGCAG
cloning *RAM1-M5 ΔN4*	MP230<br>MP226	TTTGGTCTCACACCGTGGCGTGCGAGGGG<br>TTTGGTCTCACCTTTCACTTGCATCTCCATGCAGAGGCAG
cloning *RAM1-M5 ΔC1*	MP225<br><br>MP231	TTTGGTCTCACACCAATCTGACAGTCCCTATTCCAATTGGAAT GG<br>TTTGGTCTCACCTTTCAAATGTTCCTTATCTCCGGCGCG
cloning *RAM1-M5 ΔC2*	MP225<br><br>MP232	TTTGGTCTCACACCAATCTGACAGTCCCTATTCCAATTGGAAT GG<br>TTTGGTCTCACCTTTCAGCGGCGGTTAAACATGTGTGG
cloning *RAM1-M5 ΔC3*	MP225<br><br>MP233	TTTGGTCTCACACCAATCTGACAGTCCCTATTCCAATTGGAAT GG<br>TTTGGTCTCACCTTTCATCCCACTCCGGTTATTCGGAG
cloning *RAM1-M5 ΔC4*	MP225<br><br>MP234	TTTGGTCTCACACCAATCTGACAGTCCCTATTCCAATTGGAAT GG<br>TTTGGTCTCACCTTTCACTTGGTGGTGAGTGTTGCAGC
cloning *RAM1m1*	PP5<br><br>KH314<br>KH315<br>PP12	ATGAAGACTTTACGGGTCTCACACCATGATCAATTCAATGTGT GGAAG<br>TAGAAGACAAGCGCCACAAGTTGAAGGCCACTGTC<br>TAGAAGACAAGCGCTACTTCTAGCTTGTGCTGAAGC<br>ATGAAGACTTCAGAGGTCTCACCTTGCATCTCCATGCAGAGG C

cloning *RAM1m2*	PP5<br><br>KH316<br>KH317<br>KH318<br>KH319<br>PP12	ATGAAGACTTTACGGGTCTCACACCATGATCAATTCAATGTGT GGAAG<br>TAGAAGACAAGTAGGTGCGCAAGTTGAAG<br>TAGAAGACAACTACTTGCAGCTTGTGCTGAA<br>TAGAAGACAACTCAGCAAACCTCCCCAAG<br>TAGAAGACAATGAGGCAGCGGCCTACTACTCAGCGA<br>ATGAAGACTTCAGAGGTCTCACCTTGCATCTCCATGCAGAGG C
cloning *RAM1m3*	PP5<br><br>KH320<br>KH321<br><br>KH322<br><br>KH323<br>KH324<br>KH325<br>PP12	ATGAAGACTTTACGGGTCTCACACCATGATCAATTCAATGTGT GGAAG<br>TAGAAGACAATGAGGCAGCGGCCTACTACTCAGCGA<br>TAGAAGACAATGCGGCCCTACTTGCAGCTTGTGCTGCAGCA GTGG<br>TAGAAGACAATGCGGCCCTACTTGCAGCTTGTGCTGCAGCA GTGG<br>TAGAAGACAATCCGCGCAGCGCGTGGCAGCAT<br>TAGAAGACAAGGCCGCTGCCTCAGCAAACCTCCCCAAG<br>TAGAAGACAAGGCCGCTGCCTCAGCAAACCTCCCCAAG<br>ATGAAGACTTCAGAGGTCTCACCTTGCATCTCCATGCAGAGG C
cloning *cRAM1m1*	PP5<br><br>KH314<br>KH315<br>PP57	ATGAAGACTTTACGGGTCTCACACCATGATCAATTCAATGTGT GGAAG<br>TAGAAGACAAGCGCCACAAGTTGAAGGCCACTGTC<br>TAGAAGACAAGCGCTACTTCTAGCTTGTGCTGAAGC<br>ATGAAGACTTCAGAGGTCTCACCTTTCAGCATCTCCATGCAG AGGCAG
cloning *cRAM1m2*	PP5<br><br>KH316<br>KH317<br>KH318<br>KH319<br>PP57	ATGAAGACTTTACGGGTCTCACACCATGATCAATTCAATGTGT GGAAG<br>TAGAAGACAAGTAGGTGCGCAAGTTGAAG<br>TAGAAGACAACTACTTGCAGCTTGTGCTGAA<br>TAGAAGACAACTCAGCAAACCTCCCCAAG<br>TAGAAGACAATGAGGCAGCGGCCTACTACTCAGCGA<br>ATGAAGACTTCAGAGGTCTCACCTTTCAGCATCTCCATGCAG AGGCAG
cloning *cRAM1m3*	PP5<br><br>KH320<br>KH321<br><br>KH322<br><br>KH323<br>KH324<br>KH325<br>PP57	ATGAAGACTTTACGGGTCTCACACCATGATCAATTCAATGTGT GGAAG<br>TAGAAGACAATGAGGCAGCGGCCTACTACTCAGCGA<br>TAGAAGACAATGCGGCCCTACTTGCAGCTTGTGCTGCAGCA GTGG<br>TAGAAGACAATGCGGCCCTACTTGCAGCTTGTGCTGCAGCA GTGG<br>TAGAAGACAATCCGCGCAGCGCGTGGCAGCAT<br>TAGAAGACAAGGCCGCTGCCTCAGCAAACCTCCCCAAG<br>TAGAAGACAAGGCCGCTGCCTCAGCAAACCTCCCCAAG<br>ATGAAGACTTCAGAGGTCTCACCTTTCAGCATCTCCATGCAG AGGCAG
cloning c*CBX1m1*	MP276a<br><br>AL40<br>AL41<br>MP276b	ATGAAGACTTTACGGGTCTCACACCATGGGGAAACTCTCACA GCAC<br>TTCAGAGGTCTCCTCATTGTGGCAGCAGCTGGC<br>TTTGGTCTCAATGATGGAGATGACTTCTGCT<br>ATGAAGACTTCAGAGGTCTCACCTTAACCTTCAAGCCTTCAA AGTCATCAC

cloning c*CBX1m2*	MP276a<br>AL42<br>AL41<br>MP276b	ATGAAGACTTTACGGGTCTCACACCATGGGGAAACTCTCACAGCAC<br>TTCAGAGGTCTCCTCATAAGCAAAAGCCCTAGGGCT<br>TTTGGTCTCAATGATGGAGATGACTTCTGCT<br>ATGAAGACTTCAGAGGTCTCACCTTAACCTTCAAGCCTTCAAAGTCATCAC
cloning c*CBX1m3*	MP276a<br>AL43<br>AL44<br>MP276b	ATGAAGACTTTACGGGTCTCACACCATGGGGAAACTCTCACAGCAC<br>TTCAGAGGTCTCCATTGTGTGGCAGCAGCTGGC<br>TTTGGTCTCACAATCATCTAAGTTTAAGGAGATG<br>ATGAAGACTTCAGAGGTCTCACCTTAACCTTCAAGCCTTCAAAGTCATCAC
cloning c*CBX1m4*	MP276a<br>AL45<br>AL46<br>AL47<br>AL41<br>MP276b	ATGAAGACTTTACGGGTCTCACACCATGGGGAAACTCTCACAGCAC<br>TTCAGAGGTCTCCCTGATGTGGCAGCAGCTGGC<br>TTTGGTCTCATCAGCCGGAGGGGGTGGG<br>TTCAGAGGTCTCCTCATCTCCTTAAACTTAGATGATTGACCCCCAC<br>TTTGGTCTCAATGATGGAGATGACTTCTGCT<br>ATGAAGACTTCAGAGGTCTCACCTTAACCTTCAAGCCTTCAAAGTCATCAC
cloning c*CBX1m5*	MP276a<br>AL45<br>AL48_n<br>AL49<br>AL41<br>MP276b	ATGAAGACTTTACGGGTCTCACACCATGGGGAAACTCTCACAGCAC<br>TTCAGAGGTCTCCCTGATGTGGCAGCAGCTGGC<br>TTTGGTCTCATCAGCCCTAGGGCTTTTGCT<br>TTCAGAGGTCTCCTCATCTCCTTAAACTTAGCTGCTTGAAGCAA<br>TTTGGTCTCAATGATGGAGATGACTTCTGCT<br>ATGAAGACTTCAGAGGTCTCACCTTAACCTTCAAGCCTTCAAAGTCATCAC
cloning c*CBX1m6*	MP276a<br>AL45<br>AL50<br>AL51<br>AL41<br>MP276b	ATGAAGACTTTACGGGTCTCACACCATGGGGAAACTCTCACAGCAC<br>TTCAGAGGTCTCCCTGATGTGGCAGCAGCTGGC<br>TTTGGTCTCATCAGCCCTAGGGCTTTTGCTT<br>TTCAGAGGTCTCCTCATCCCCTTAAACTTAGATGATTGAAGCA<br>TTTGGTCTCAATGATGGAGATGACTTCTGCT<br>ATGAAGACTTCAGAGGTCTCACCTTAACCTTCAAGCCTTCAAAGTCATCAC
cloning c*CBX1m7*	MP276a<br>AL45<br>AL52<br>AL53<br>AL41<br>MP276b	ATGAAGACTTTACGGGTCTCACACCATGGGGAAACTCTCACAGCAC<br>TTCAGAGGTCTCCCTGATGTGGCAGCAGCTGGC<br>TTTGGTCTCATCAGCCCTAGGGCTTTTGCTTC<br>TTCAGAGGTCTCCTCATCTCCTTACCCTTAGATGATTGAAGC<br>TTTGGTCTCAATGATGGAGATGACTTCTGCT<br>ATGAAGACTTCAGAGGTCTCACCTTAACCTTCAAGCCTTCAAAGTCATCAC
cloning p*35Smin*	TZ109<br>TZ110	CATGGTCTCATCTGTGCAGCAAGACCCTTCCTCTA<br>CCCGGTCTCTGGTGTGGGCTGTCCTCTCCAAATG
cloning *cCBX1* into pGEX-6P-1	PGEX-CBX1-F<br>PGEX-CBX1-R	TAGAATTCACCGGGAAACTCTCACAG<br>ATCTCGAGTTAAACCTTCAAGCCTTCAAAGTCA

Table S5. Plasmids used in this study.

purpose	name	description
**LI golden gate plasmids**		
	LI C-D *GUS* (pPPLI3)	(14)
	LI C-D *RAM1* (pPPLI2)	(14)
	LI C-D *DELLA1*$^{\square 17}$(pPPLI5)	(14)
	LI C-D *RAM2*	(44)
	LI C-D *STR* (pCC022)	Assembled by Assembled by BpiI cut ligation from: LI pENTR BpiI + PCR amplicons CC66 + CC67, CC69 + CC70, CC71 + CC72, CC73 + CC74, CC75 + CC76, CC77 + CC155 amplified from Gifu genomic DNA
	LI C-D *CBX1* (pMP042)	Assembled by BpiI cut ligation from: BB52 + PCR amplicon MP276a + 276b amplified from Gifu genomic DNA
	LI C-D *WRI3* (pMP041)	Assembled by BpiI cut ligation from: BB52 + PCR amplicons MP277 + MP278, MP279 + MP280, MP281 + MP282 amplified from Gifu genomic DNA
	LI C-D *WRI5a* (pMP038)	Assembled by BpiI cut ligation from: BB52 + PCR amplicons MP258 + MP259, MP260 + MP261 amplified from Gifu genomic DNA
	LI C-D *WRI5b* (pMP039)	Assembled by BpiI cut ligation from: BB52 + PCR amplicons MP262 + MP263, MP264 + MP265, MP266 + MP267 amplified from Gifu genomic DNA
	LI C-D *WRI5c* (pMP040)	Assembled by BpiI cut ligation from: BB52 + PCR amplicons MP268 + MP269, MP270 + MP271, MP272 + MP273, MP274 + MP275 amplified from Gifu genomic DNA
	LI C-D *cCBX1* (pMP058)	Assembled by BpiI cut ligation from: BB3 + PCR amplicons MP276a + MP276b amplified from Gifu cDNA
	LI C-D *cWRI3.1* (pMP056)	Assembled by BpiI cut ligation from: BB3 + PCR amplicons MP277 + MP278, MP279 + MP282 amplified from Gifu cDNA
	LI C-D *cWRI3.2* (pMP057)	Assembled by BpiI cut ligation from: BB3 + PCR amplicons MP277 + MP278, MP279 + MP282 amplified from Gifu cDNA
	LI C-D *cWRI5a* (pMP053)	Assembled by BpiI cut ligation from: BB3 + PCR amplicon MP258 + MP261 amplified from Gifu cDNA

	LI C-D *cWRI5b* (pMP054)	Assembled by BpiI cut ligation from: BB3 + PCR amplicons MP262 + MP263, MP264 + MP265, MP266 + MP267 amplified from Gifu cDNA
	LI C-D *cWRI5c* (pMP055)	Assembled by BpiI cut ligation from: BB3 + PCR amplicons MP268 + MP269, MP270 + MP273, MP274 + MP275 amplified from Gifu cDNA
	LI A-B p*RAM2*	(44)
	LI A-B p*RAM2m* (pMP043)	Assembled by BpiI cut ligation from: BB3 + PCR amplicons MP420 + MP421, MP422 + MP423, MP424 + MP425, MP426 + MP427, MP428 + MP429 amplified from LI A-B p*RAM2*
	LI A-B p*RAM2mAW* (pMP045)	Assembled by BpiI cut ligation from: BB3 + PCR amplicons MP420 + MP421, MP422 + MP425, MP426 + MP427, 428 + MP429 amplified from LI A-B p*RAM2*
	LI A-B p*RAM2mMYCS* (pMP044)	Assembled by BpiI cut ligation from: BB3 + PCR amplicons MP420 + MP423, MP424 + MP429 amplified from LI A-B p*RAM2*
	LI A-B p*PT4*	(44)
	LI A-B p*PT4m* (pMP046)	Assembled by BpiI cut ligation from: BB3 + PCR amplicons MP458 + MP459, MP460 + MP461, MP462 + MP463, MP464 + MP465 amplified from LI A-B p*PT4*
	LI A-B p*PT4mAW* (pMP070)	Assembled by BpiI cut ligation from: BB3 + PCR amplicons MP458 + MP459, MP460 + MP461, MP462 + MP476, MP476 + MP465 amplified from LI A-B p*PT4*
	LI A-B p*PT4mMYCS* (LI AL24)	Assembled by BpiI cut ligation from: BB3 + PCR amplicons MP458 + AL35, AL36 + MP463, MP464 + MP465 amplified from LI A-B p*PT4*
	LI A-B p*STR*	Assembled by BpiI cut ligation from: BB3 + PCR amplicon MP452 + MP470 amplified from Gifu genomic DNA
	LI A-B p*STRm*	Assembled by BpiI cut ligation from: BB3 + PCR amplicons MP452 + MP453, MP454 + MP455, MP456 + MP468, MP469 + MP470 amplified from LI A-B p*STR*
	LI A-B p*RAM1* (pPPLI1)	(14)
	LI A-B p*WRI5b* (pMP030)	Assembled by BpiI cut ligation from: BB3 + PCR amplicons MP406 + MP407, MP408 + MP409 amplified from Gifu genomic DNA

	LI C-D *cRAM1m1* (pKH308)	Assembled by BpiI cut ligation from: BB52 + PCR amplicons PP5 + KH314, KH315 + PP12 amplified from Gifu cDNA
	LI C-D *cRAM1m2* (pKH309)	Assembled by BpiI cut ligation from: BB52 + PCR amplicons PP5 + KH316, KH317 + KH318, KH319 + PP12 amplified from Gifu cDNA
	LI C-D *cRAM1m3* (pKH310)	Assembled by BpiI cut ligation from: BB52 + PCR amplicons PP5 + KH320, KH321 + KH322, KH323 + KH324, KH325 + PP12 amplified from Gifu cDNA
	LI C-D *cCBX1m1* (pMP72)	Assembled by BpiI cut ligation from: BB3 + PCR amplicons of MP276a + AL40, AL41 + MP276b amplified from Gifu cDNA
	LI C-D *cCBX1m2* (pMP73)	Assembled by BpiI cut ligation from: BB3 + PCR amplicons of MP276a + AL42, AL41 + MP276b amplified from Gifu cDNA
	LI C-D *cCBX1m3* (pMP74)	Assembled by BpiI cut ligation from: BB3 + PCR amplicons of MP276a + AL43, AL44 + MP276b amplified from Gifu cDNA
	LI C-D *cCBX1m4* (pMP75)	Assembled by BpiI cut ligation from: BB3 + PCR amplicons of MP276a + AL40, AL46 + AL47, AL41 + MP276b amplified from Gifu cDNA
	LI C-D *cCBX1m5* (pMP76)	Assembled by BpiI cut ligation from: BB3 + PCR amplicons of MP276a + AL40, AL48_n + AL49, AL41 + MP276b amplified from Gifu cDNA
	LI C-D *cCBX1m6* (pMP77)	Assembled by BpiI cut ligation from: BB3 + PCR amplicons of MP276a + AL40, AL50 + AL51, AL41 + MP276b amplified from Gifu cDNA
	LI C-D *cCBX1m7* (pMP78)	Assembled by BpiI cut ligation from: BB3 + PCR amplicons of MP276a + AL40, AL52 + AL53, AL41 + MP276b amplified from Gifu cDNA
	LI D-E *nCitrine* (pPP27)	
	LI D-E *cCitrine* (pPP26)	
	LI B-C p*35Smin* (pTZI9)	Assembled by BpiI cut ligation from: BB3 and PCR amplicon TZ109 + TZ110

LII golden gate plasmids		
	LIIβ F 1-2 p*Ubi*:*mCherry* (pPPLII24)	Assembled by BsaI cut ligation from:<br>LI A-B p*Ubi* (G007) + LI B-C dy (BB06) + LI C-D *mCherry* (G023) + LI D-E dy (BB08) + LI E-F *35S*-T (G059) + LI F-G dy (BB09) + LIIβ F 1-2 (BB20)
	LIIβ F 5-6 p*Ubi*:*mCherry* (pMP200)	Assembled by BsaI cut ligation from:<br>LI A-B p*Ubi* (G007) + LI B-C dy (BB06) + LI C-D *mCherry* (G023) + LI D-E dy (BB08) + LI E-F *35S*-T (G059) + LI F-G dy (BB09) + LIIβ F 5-6 (BB28)
	LIIc R 3-4 p*35S*:*mCherry* (pPPLII7)	Assembled by BsaI cut ligation from:<br>LI A-B p*35S* (G005) + LI B-C dy (BB06) + LI C-D *mCherry* (G023) + LI D-E dy (BB08) + LI E-F *35S*-T (G059) + LI F-G dy (BB09) + LIIc R 3-4 (BB34)
	LIIc R 3-4 p*Ubi*:*mCherry* (pPPLII8)	Assembled by BsaI cut ligation from:<br>LI A-B p*Ubi* (G006) + LI B-C dy (BB06) + LI C-D *mCherry* (G023) + LI D-E dy (BB08) + LI E-F *35S*-T (G059) + LI F-G dy (BB09) + LIIc R 3-4 (BB34)
	LIIc F 3-4 pOI*:GUS* (pPPLII22)	Assembled by BsaI cut ligation from:<br>LI A-B Esp3I-*lacZ* dy (G082) + LI B-C dy (BB06) + LI C-D *GUS* + LI D-E dy (BB08) + LI *nos*-T (G006) + LI F-G dy (BB09) + LIIc F 3-4 (BB33)
	LIIc F 1-2 p*Ubi*:*RAM1* (pPPLII9)	(14)
	LIIc F 1-2 p*Ubi*:*Myc-RAM1* (pPPLII16)	Assembled by BsaI cut ligation from:<br>LI A-B p*Ubi* (G007) + LI B-C *c-Myc* (G069) + LI C-D *RAM1* + LI D-E dy (BB08) + LI E-F *nos*-T (G006) + LI F-G dy (BB09) + LIIc F 1-2 (BB30)
	LIIβ F 2-3 p*Ubi*:*WRI3* (pMP609)	Assembled by BsaI cut ligation from:<br>LI A-B p*Ubi* (G007) + LI B-C dy (BB06) + LI C-D *WRI3* + LI D-E dy (BB08) + LI E-F *nos*-T (G006) + LI F-G dy (BB09) + LIIβ F 2-3 (BB22)
	LIIβ F 3-4 p*Ubi*:*CBX1* (pMP612)	Assembled by BsaI cut ligation from:<br>LI A-B p*Ubi* (G007) + LI B-C dy (BB06) + LI C-D *CBX1* + LI D-E dy (BB08) + LI E-F *nos*-T (G006) + LI F-G dy (BB09) + LIIβ F 3-4 (BB24)
	LIIβ F 1-2 p*Ubi*:*WRI5a* (pMP600)	Assembled by BsaI cut ligation from:<br>LI A-B p*Ubi* (G007) + LI B-C dy (BB06) + LI C-D *WRI5a* + LI D-E dy (BB08) + LI E-F *nos*-T (G006) + LI F-G dy (BB09) + LIIβ F 1-2 (BB20)

	LIIβ F 1-2 p*Ubi*:*WRI5b* (pMP603)	Assembled by BsaI cut ligation from:<br>LI A-B p*Ubi* (G007) + LI B-C dy (BB06) + LI C-D *WRI5b* + LI D-E dy (BB08) + LI E-F *nos*-T (G006) + LI F-G dy (BB09) + LIIβ F 1-2 (BB20)
	LIIβ F 1-2 p*Ubi*:*WRI5c* (pMP606)	Assembled by BsaI cut ligation from:<br>LI A-B p*Ubi* (G007) + LI B-C dy (BB06) + LI C-D *WRI5c* + LI D-E dy (BB08) + LI E-F *nos*-T (G006) + LI F-G dy (BB09) + LIIβ F 1-2 (BB20)
	LIIβ F 3-4 p*RAM2*:*GUS* (pMP631)	Assembled by BsaI cut ligation from:<br>LI A-B p*RAM2* + LI B-C dy (BB06) + LI C-D *GUS* + LI D-E dy (BB08) + LI E-F *nos*-T (G006) + LI F-G dy (BB09) + LIIβ F 3-4 (BB24)
	LIIβ F 3-4 p*RAM2m*:*GUS* (pMP632)	Assembled by BsaI cut ligation from:<br>LI A-B p*RAM2m* + LI B-C dy (BB06) + LI C-D *GUS* + LI D-E dy (BB08) + LI E-F *nos*-T (G006) + LI F-G dy (BB09) + LIIβ F 3-4 (BB24)
	LIIβ F 3-4 p*STR*:*GUS* (pMP633)	Assembled by BsaI cut ligation from:<br>LI A-B p*STR* + LI B-C dy (BB06) + LI C-D *GUS* + LI D-E dy (BB08) + LI E-F *nos*-T (G006) + LI F-G dy (BB09) + LIIβ F 3-4 (BB24)
	LIIβ F 3-4 p*STRm*:*GUS* (pMP634)	Assembled by BsaI cut ligation from:<br>LI A-B p*STRm* + LI B-C dy (BB06) + LI C-D *GUS* + LI D-E dy (BB08) + LI E-F *nos*-T (G006) + LI F-G dy (BB09) + LIIβ F 3-4 (BB24)
	LIIβ F 1-2 p*RAM2m*:*RAM2* (pMP635)	Assembled by BsaI cut ligation from:<br>LI A-B p*RAM2m* + LI dy B-C (BB06) + LI C-D *RAM2* + LI D-E dy (BB08) + LI E-F *nos*-T (G006) + LI F-G dy (BB09) + LIIβ F 1-2 (BB20)
	LIIc 1-2 p*STR*:*STR* (pCC027)	Assembled by BsaI cut ligation from:<br>LI A-B p*STR* + LI B-C ATG dy + LI C-D *STR* + LI D-E dy (BB08) + LI E-F *nost*-T + LI F-G dy (BB09) + LIIc F 1-2 (BB30)
	LIIc 1-2 p*STRm*:*STR* (pCC031)	Assembled by BsaI cut ligation from:<br>LI A-B p*STRm* + LI B-C ATG dy + LI C-D *STR* + LI D-E dy (BB08) + LI E-F *nost*-T + LI F-G dy (BB09) + LIIc F 1-2 (BB30)

	LIIc F 3-4 p*2xCCTTGT_35Smin*	Assembled by BsaI cut ligation from: PCR amplicon KH190 + KH191 + LI B-C p*35Smin*, LI C-D dy (BB07), LI D-E dy (BB08), LI E-F *Hsp*T (G045), LI F-G dy (BB09), LIIc F 3-4 (BB33)
	LIIc F 3-4 p*4xCCTTGT_35Smin*	Assembled by BsaI cut ligation from: PCR amplicon KH188 + KH189 + LI B-C p*35Smin*, LI C-D dy (BB07), LI D-E dy (BB08), LI E-F *Hsp*T (G045), LI F-G dy (BB09), LIIc F 3-4 (BB33)
	LIIβ F 1-2 p*Ubi*:*Myc-RAM1m1* (pKH315)	Assembled by BsaI cut ligation from: LI A-B p*Ubi* (G007) + LI B-C *c-Myc* (G069) + LI C-D *RAM1m1* + LI D-E dy (BB08) + LI E-F *nos*-T (G006) + LI F-G dy (BB09) + LIIβ F 1-2 (BB20)
	LIIβ F 1-2 p*Ubi*:*Myc-RAM1m2* (pKH316)	Assembled by BsaI cut ligation from: LI A-B p*Ubi* (G007) + LI B-C *c-Myc* (G069) + LI C-D *RAM1m2* + LI D-E dy (BB08) + LI E-F *nos*-T (G006) + LI F-G dy (BB09) + LIIβ F 1-2 (BB20)
	LIIβ F 1-2 p*Ubi*:*Myc-RAM1m3* (pKH317)	Assembled by BsaI cut ligation from: LI A-B p*Ubi* (G007) + LI B-C *c-Myc* (G069) + LI C-D *RAM1m3* + LI D-E dy (BB08) + LI E-F *nos*-T (G006) + LI F-G dy (BB09) + LIIβ F 1-2 (BB20)
	LIIβ F 3-4 p*RAM1*:*RAM1-nCit* (pSHL_2-63)	Assembled by BsaI cut ligation from: LI A-B p*RAM1* + LI B-C dy (BB06) + LI C-D *RAM1* + LI D-E *nCitrine* + LI E-F n*os*-T (G006) + LI F-G dy (BB09) + LIIβ F 3-4 (BB24)
	LIIβ F 3-4 p*RAM1*:*RAM1m3-nCit* (pSHL_2-78)	Assembled by BsaI cut ligation from: LI A-B p*RAM1* + LI B-C dy (BB06) + LI C-D *RAM1m3* + LI D-E *nCitrine* + LI E-F *nos*-T (G006) + LI F-G dy (BB09) + LIIβ F 3-4 (BB24)

	LIIβ F 5-6 p*CBX1*:*CBX1-cCit* (pSHL_2-59)	Assembled by BsaI cut ligation from: LI A-B p*CBX1* + LI B-C dy (BB06) + LI C-D *CBX1* + LI D-E *cCitrine* + LI E-F *nos*-T (G006) + LI F-G dy (BB09) + LIIβ F 5-6 (BB28)
	LIIβ F 5-6 p*CBX1*:*CBX1m4-cCit* (pSHL_2-60)	Assembled by BsaI cut ligation from: LI A-B p*CBX1* + LI B-C dy (BB06) + LI C-D *CBX1m4* + LI D-E *cCitrine* + LI E-F *nos*-T (G006) + LI F-G dy (BB09) + LIIβ F 5-6 (BB28)
	LIIβ F 5-6 p*CBX1*:*CBX1m7-cCit* (pSHL_2-61)	Assembled by BsaI cut ligation from: LI A-B p*CBX1* + LI B-C dy (BB06) + LI C-D *CBX1m7* + LI D-E *cCitrine* + LI E-F *nos*-T (G006) + LI F-G dy (BB09) + LIIβ F 5-6 (BB28)
	LIIβ F 5-6 p*WRI5b*:*WRI5b-cCit* (pSHL_2-62)	Assembled by BsaI cut ligation from: LI A-B p*WRI5b* + LI B-C dy (BB06) + LI C-D *WRI5b* (pMP39) + LI D-E *cCitrine* (pPP24) + LI E-F *nos*-T (G006) + LI F-G dy (BB09) + LIIβ F 5-6 (BB28)
	LIIβ F 1-2 p*Ubi:CBX1-Myc* (pMP642)	Assembled by BsaI cut ligation from:<br>LI A-B p*Ubi* (G007) + LI B-C dy (BB06) + LI C-D c*CBX1* + LI D-E *c-Myc* (G070) + LI E-F *nos*-T (G006) + LI F-G dy (BB09) + LIIβ F 1-2 (BB20)
	LIIβ F 1-2 p*Ubi*:*Myc-CBX1m1* (pMP686)	Assembled by BsaI cut ligation from:<br>LI A-B p*Ubi* (G007) + LI B-C *c-Myc* (G069) + LI C-D *CBX1m1* + LI D-E dy (BB08) + LI E-F *nos*-T (G006) + LI F-G dy (BB09) + LIIβ F 1-2 (BB20)
	LIIβ F 1-2 p*Ubi*:*Myc-CBX1m2* (pMP687)	Assembled by BsaI cut ligation from:<br>LI A-B p*Ubi* (G007) + LI B-C *c-Myc* (G069) + LI C-D *CBX1m2* + LI D-E dy (BB08) + LI E-F *nos*-T (G006) + LI F-G dy (BB09) + LIIβ F 1-2 (BB20)
	LIIβ F 1-2 p*Ubi*:*Myc-CBX1m3* (pMP688)	Assembled by BsaI cut ligation from:<br>LI A-B p*Ubi* (G007) + LI B-C *c-Myc* (G069) + LI C-D *CBX1m3* + LI D-E dy (BB08) + LI E-F *nos*-T (G006) + LI F-G dy (BB09) + LIIβ F 1-2 (BB20)
	LIIβ F 1-2 p*Ubi*:*Myc-CBX1m4* (pMP689)	Assembled by BsaI cut ligation from:<br>LI A-B p*Ubi* (G007) + LI B-C *c-Myc* (G069) + LI C-D *CBX1m4* + LI D-E dy (BB08) + LI E-F *nos*-T (G006) + LI F-G dy (BB09) + LIIβ F 1-2 (BB20)
	LIIβ F 1-2 p*Ubi*:*Myc-CBX1m5* (pMP690)	Assembled by BsaI cut ligation from:

		LI A-B p*Ubi* (G007) + LI B-C *c-Myc* (G069) + LI C-D *CBX1m5* + LI D-E dy (BB08) + LI E-F *nos*-T (G006) + LI F-G dy (BB09) + LIIβ F 1-2 (BB20)
	LIIβ F 1-2 p*Ubi*:*Myc-CBX1m6* (pMP691)	Assembled by BsaI cut ligation from:<br>LI A-B p*Ubi* (G007) + LI B-C *c-Myc* (G069) + LI C-D *CBX1m6* + LI D-E dy (BB08) + LI E-F *nos*-T (G006) + LI F-G dy (BB09) + LIIβ F 1-2 (BB20)
	LIIβ F1-2 p*Ubi*:*Myc-CBX1m7* (pMP692)	Assembled by BsaI cut ligation from:<br>LI A-B p*Ubi* (G007) + LI B-C *c-Myc* (G069) + LI C-D *CBX1m7* + LI D-E dy (BB08) + LI E-F *nos*-T (G006) + LI F-G dy (BB09) + LIIβ F 1-2 (BB20)
	LIIβ F1-2 p*Ubi*:*WRI5b-GFP* (pMP605)	Assembled by BsaI cut ligation from:<br>LI A-B p*Ubi* (G007) + LI B-C dy (BB06) + LI C-D *WRI5b* + LI D-E *linker GFP* (G011) + LI E-F *nos*-T (G006) + LI F-G dy (BB09) + LIIβ F 1-2 (BB20)
	LIIβ F 1-2 p*Ubi*:*CBX1-GFP* (pMP614)	Assembled by BsaI cut ligation from:<br>LI A-B p*Ubi* (G007) + LI B-C dy (BB06) + LI C-D *CBX1* + LI D-E *linker GFP* (G011) + LI E-F *nos*-T (G006) + LI F-G dy (BB09) + LIIβ F 1-2 (BB20)
	LIIc R 5-6 p*35S*:*GFP* (pPPLII29)	Assembled by BsaI cut ligation from: LI A-B p*35S* (G005) + LI B-C dy (BB06) + LI C-D *GFP* (G19) + LI D-E dy (BB08) + LI E-F *35S*-T (G059) + LI F-G dy (BB09) + LIIc R 5-6 (BB19)
	LIIc F 1-2 p*Ubi*:*Myc-DELLA1*$^{\square 17}$ (pPPLII15)	Assembled by BsaI cut ligation from: LI A-B p*Ubi* (G007) + LI B-C *c-Myc* (G069) + LI C-D *DELLA1*$^{\square 17}$ + LI D-E dy (BB08) + LI E-F *nos*-T (G006) + LI F-G dy (BB09) + LIIc F 1-2 (BB30)
	LIIβ F 3-4 p*RAM1*:*HA-RAM1* (pSHL_2-75)	Assembled by BsaI cut ligation from: LI A-B p*RAM1* + LI B-C *HA* (G67) + LI C-D *RAM1* + LI D-E dy (BB08) + LI E-F *Nos*T (G006) + LI F-G dy (BB09) + LIIβ F 3-4 (BB24)
	LIIβ F 1-2 p*RAM1*:*HA-RAM1m2* (pSHL_2-79)	Assembled by BsaI cut ligation from: LI A-B p*RAM1* + LI B-C *HA* (G67) + LI C-D *RAM1m2* + LI D-E dy (BB08) + LI E-F *Nos*T (G006) + LI F-G dy (BB09) + LIIβ F 1-2 (BB20)

**LIII golden gate plasmids**		
Esp3I compatible destination backbone for localization of promoter activity	LIIIβ fin<br>p*Ubi*:*mCherry*_pOI*:GUS* Esp3I (pMP301)	Assembled by BpiI cut ligation from:<br>LIIβ F 1-2 p*Ubi*:*mCherry* + LII 2-3 ins (BB43) + LIIc F 3-4 pOI:*GUS* + LII dy 4-6 (BB41) + LIIIβ fin (BB52)
BsaI compatible destination backbone for localization of promoter activity	LIIIβ fin p*Ubi*:*mCherry*_pOI*:GUS* BsaI (pMP302)	Assembled by Esp3I cut ligation from:<br>LIIIβ fin p*Ubi*:*mCherry*_pOI:*GUS* Esp3I + LI A-B Esp3I-*ccdB* dy (G084)
EV for hairy root experiments excluding GUS staining	LIIIβ F A-B p*RAM1*:*GUS* (pPPLIII5)	(14)
Transactivation assay, Overexpression of *RAM1*	LIIIβ F A-B p*Ubi*: *Myc-RAM1* (pPPLIII24)	Assembled by BpiI cut ligation from:<br>LIIc F 1-2 p*Ubi*:*Myc-RAM1* + LII 2-3 ins (BB43) + LIIc R 3-4 p*35S*:*mCherry* + LII dy 4-6 (BB41) + LIIIβ F A-B (BB53)
Localization of *RAM2* promoter activity , EV for *ram2* complementation, transactivation assay	LIIIβ fin p*RAM2*:*GUS* (pPPLIII79)	Assembled by BsaI cut ligation from: LIIIβ fin p*Ubi*:*mCherry*_pOI:*GUS* BsaI + LI A-B p*RAM2*
Localization of *RAM2* promoter activity , EV for *ram2* complementation, transactivation assay	LIIIβ fin p*RAM2m*:*GUS* (pMP494)	Assembled by BsaI cut ligation from: LIIIβ fin p*Ubi*:*mCherry*_pOI:*GUS* BsaI + LI A-B p*RAM2m*
Transactivation assay	LIIIβ fin p*RAM2mAW*:*GUS* (pMP495)	Assembled by BsaI cut ligation from: LIIIβ fin p*Ubi*:*mCherry*_pOI:*GUS* BsaI + LI A-B p*RAM2mAW*
Transactivation assay	LIIIβ fin p*RAM2mMYCS*:*GUS* (pMP496)	Assembled by BsaI cut ligation from: LIIIβ fin p*Ubi*:*mCherry*_pOI:*GUS* BsaI + LI A-B p*RAM2mMYCS*
Transactivation assay	LIIIβ fin p*PT4:GUS* (pMP390)	Assembled by BsaI cut ligation from: LIIIβ fin p*Ubi*:*mCherry*_pOI*:GUS* BsaI + LI A-B p*PT4*
Transactivation assay	LIIIβ fin p*PT4m:GUS* (pMP498)	Assembled by BsaI cut ligation from: LIIIβ fin p*Ubi*:*mCherry*_pOI*:GUS* BsaI + LI A-B p*PT4m*
Transactivation assay	LIIIβ fin p*PT4mAW:GUS* (LIII AL20)	Assembled by BsaI cut ligation from: LIIIβ fin p*Ubi*:*mCherry*_pOI*:GUS* BsaI + LI A-B p*PT4mAW*
Transactivation assay	LIIIβ fin p*PT4mMYCS:GUS* (LIII AL21)	Assembled by BsaI cut ligation from: LIIIβ fin p*Ubi*:*mCherry*_pOI*:GUS* BsaI + LI A-B p*PT4mMYCS*

Transactivation assay	LIIIβ fin p*WRI3*:*GUS* (pMP477)	Assembled by Esp3I cut ligation from: LIIIβ fin p*Ubi*:*mCherry*_pOI:*GUS*_Esp3I + PCR amplicon of MP303 and MP304 amplified from Gifu genomic DNA
Transactivation assay	LIIIβ fin p*CBX1*:*GUS* (pMP478)	Assembled by Esp3I cut ligation from: LIIIβ fin p*Ubi*:*mCherry*_pOI:*GUS*_Esp3I + PCR amplicon of MP301 and MP302 amplified from Gifu genomic DNA
Transactivation assay	LIIIβ fin p*WRI5a*:*GUS* (pMP474)	Assembled by Esp3I cut ligation from: LIIIβ fin p*Ubi*:*mCherry*_pOI:*GUS*_Esp3I + PCR amplicon of MP293 and MP294 amplified from Gifu genomic DNA
Transactivation assay	LIIIβ fin p*WRI5b*:*GUS* (pMP475)	Assembled by BsaI cut ligation from: LIIIβ fin p*Ubi*:*mCherry*_pOI:*GUS*_BsaI + LI A-B p*WRI5b*
Transactivation assay	LIIIβ fin p*WRI5c*:*GUS* (pMP476)	Assembled by Esp3I cut ligation from: LIIIβ fin p*Ubi*:*mCherry*_pOI:*GUS*_Esp3I + PCR amplicon of MP299 and 300 amplified from Gifu genomic DNA
Transactivation assay	LIIIβ fin p*CBX1m*:*GUS* (pMP493)	Assembled by Esp3I cut ligation from: LIIIβ fin p*Ubi*:*mCherry*_pOI:*GUS*_Esp3I + PCR amplicons of MP301 + 330, MP329 + MP302 and 300 amplified from LIIIβ fin p*CBX1*:*GUS*
Transactivation assay	LIIIβ fin p*WRI3m*:*GUS* (LIII AL28)	Assembled by Esp3I cut ligation from: LIIIβ fin p*Ubi*:*mCherry*_pOI:*GUS*_Esp3I + PCR amplicons of AL76 + AL77, AL78 + AL79, AL80 + AL81 amplified from LIIIβ fin p*WRI3*:*GUS*
Transactivation assay	LIIIβ fin p*WRI5am*:*GUS* (pKH130)	Assembled by BsaI cut ligation from: LIIIβ fin p*Ubi*:*mCherry*_pOI:*GUS*_BsaI + PCR amplicons of MP400 + MP479, MP480 + MP481, MP482 + KH104, KH105 + MP405 amplified from LIIIβ fin p*WRI5a*:*GUS*
Transactivation assay	LIIIβ fin p*WRI5bm*:*GUS* (pKH116)	Assembled by BsaI cut ligation from: LIIIβ fin p*Ubi*:*mCherry*_pOI:*GUS*_BsaI + PCR amplicons of MP406 + MP483, MP484 + MP409 amplified from LIIIβ fin p*WRI5b*:*GUS*

Transactivation assay	LIIIβ fin p*WRI5cm*:*GUS* (pKH118)	Assembled by BsaI cut ligation from: LIIIβ fin p*Ubi*:*mCherry*_pOI:*GUS*_BsaI + PCR amplicons of MP410 + MP537. MP538 + MP487, MP488 + MP411 amplified from LIIIβ fin p*WRI5c*:*GUS*
Transactivation assay	LIIIβ fin p*WRI5b_-531*:*GUS* (pKH151)	Assembled by BsaI cut ligation from: LIIIβ fin p*Ubi*:*mCherry*_pOI:*GUS*_BsaI + PCR amplicon of KH158 + MP409 amplified from LIIIβ fin p*WRI5b*:*GUS*
Transactivation assay	LIIIβ fin p*WRI5b_-293*:*GUS* (pKH150)	Assembled by BsaI cut ligation from: LIIIβ fin p*Ubi*:*mCherry*_pOI:*GUS*_BsaI + PCR amplicon of KH159 + MP409 amplified from LIIIβ fin p*WRI5b*:*GUS*
Transactivation assay	LIIIβ fin p*WRI5b_-161*:*GUS* (pKH149)	Assembled by BsaI cut ligation from: LIIIβ fin p*Ubi*:*mCherry*_pOI:*GUS*_BsaI + PCR amplicon of KH160 + MP409 amplified from LIIIβ fin p*WRI5b*:*GUS*
Transactivation assay	LIIIβ fin p*WRI5b_-268*:*GUS* (pKH159)	Assembled by BsaI cut ligation from: LIIIβ fin p*Ubi*:*mCherry*_pOI:*GUS*_BsaI + PCR amplicon of KH161 + MP409 amplified from LIIIβ fin p*WRI5b*:*GUS*
Transactivation assay	LIIIβ fin p*WRI5b_-239*:*GUS* (pKH160)	Assembled by BsaI cut ligation from: LIIIβ fin p*Ubi*:*mCherry*_pOI:*GUS*_BsaI + PCR amplicon of KH162 + MP409 amplified from LIIIβ fin p*WRI5b*:*GUS*
Transactivation assay	LIIIβ fin p*WRI5b_-215*:*GUS* (pKH161)	Assembled by BsaI cut ligation from: LIIIβ fin p*Ubi*:*mCherry*_pOI:*GUS*_BsaI + PCR amplicon of KH163 + MP409 amplified from LIIIβ fin p*WRI5b*:*GUS*
Transactivation assay	LIIIβ fin p*WRI5b_-187*:*GUS* (pKH162)	Assembled by BsaI cut ligation from: LIIIβ fin p*Ubi*:*mCherry*_pOI:*GUS*_BsaI + PCR amplicon of KH164 + MP409 amplified from LIIIβ fin p*WRI5b*:*GUS*
Transactivation assay	LIIIβ fin p*WRI5bmCCTTGT*:*GUS* (pKH178)	Assembled by BsaI cut ligation from: LIIIβ fin p*Ubi*:*mCherry*_pOI:*GUS*_BsaI + PCR amplicon of MP406 + KH186 and KH187 + MP409 from LIIIβ fin p*WRI5b*:*GUS*
Transactivation assay	LIIIβ fin p*2xCCTTGT_35Smin*:*GUS* (pKH183)	Assembled by BsaI cut ligation from: LIIIβ fin p*Ubi*:*mCherry*_pOI:*GUS*_BsaI + PCR amplicon of KH192 + KH199 from LIIc F 3-4 p2xCCTTGT_35Smin

Transactivation assay	LIIIβ fin p*4xCCTTGT_35Smin*:*GUS* (pKH184)	Assembled by BsaI cut ligation from: LIIIβ fin p*Ubi*:*mCherry*_pOI:*GUS*_BsaI + PCR amplicon of KH194 + KH199 from LIIc F 3-4 p4xCCTTGT_35Smin
Overexpression of *WRI3*	LIIIβ F A-B p*Ubi*:*WRI3* (pMP480)	Assembled by BpiI cut ligation from:<br>LII dy 1-2 (BB63) + LIIβ F 2-3 p*Ubi*:*WRI3* + LII dy 3-4 (BB64) LII 4-5 ins (BB44) + LIIβ F 5-6 p*Ubi*:*mCherry* + LIIIβ F A-B (BB53)
Overexpression of *CBX1*	LIIIβ F A-B p*Ubi*:*CBX1* (pMP481)	Assembled by BpiI cut ligation from:<br>LII dy 1-3 (BB38) + LIIβ F 3-4 p*Ubi*:*CBX1* + LII 4-5 ins (BB44) + LIIβ F 5-6 p*Ubi*:*mCherry* + LIIIβ F A-B (BB53)
Overexpression of *WRI5a*	LIIIβ F A-B p*Ubi*:*WRI5a* (pMP479)	Assembled by BpiI cut ligation from:<br>LIIβ F 1-2 p*Ubi*:*WRI5a* + LII ins 2-3 (BB43) + LII dy 3-4 (BB64) + LII 4-5 ins (BB44) + LIIβ F 5-6 p*Ubi*:*mCherry* + LIIIβ F A-B (BB53)
Overexpression of *WRI5b*	LIIIβ F A-B p*Ubi*:*WRI5b* (pMP504)	Assembled by BpiI cut ligation from:<br>LIIβ F 1-2 p*Ubi*:*WRI5b* + LII ins 2-3 (BB43) + LII dy 3-4 (BB64) + LII 4-5 ins (BB44) + LIIβ F 5-6 p*Ubi*:*mCherry* + LIIIβ F A-B (BB53)
Overexpression of *WRI5c*	LIIIβ F A-B p*Ubi*:*WRI5c* (pMP505)	Assembled by BpiI cut ligation from:<br>LIIβ F 1-2 p*Ubi*:*WRI5c* + LII ins 2-3 (BB43) + LII dy 3-4 (BB64) + LII 4-5 ins (BB44) + LIIβ F 5-6 p*Ubi*:*mCherry* + LIIIβ F A-B (BB53)
Complementation of the *ram2* mutant	LIIIβ F A-B p*RAM2*:*RAM2*	(44)
Complementation of the *ram2* mutant	LIIIβ F A-B p*RAM2m*:*RAM2* (pMP497)	Assembled by BpiI cut ligation from:<br>LIIβ F 1-2 p*RAM2m*:*RAM2* + LII ins 2-3 (BB43) + LII dy 3-4 (BB64) + LII 4-5 ins (BB44) + LIIβ F 5-6 p*Ubi*:*mCherry* + LIIIβ F A-B (BB53)
Complementation of the *str* mutant	LIIIβ fin p*STR*:*STR* (pCC037)	Assembled by BpiI cut ligation from:<br>LIIc F 1-2 p*STR*:*STR* + LII ins 2-3 (BB43) + LIIc R 3-4 p*Ubi*:*mCherry* + LII 4-6 dy (BB41)
Complementation of the *str* mutant	LIIIβ fin p*STRm*:*STR* (pCC038)	Assembled by BpiI cut ligation from:<br>LIIc F 1-2 p*STRm*:*STR* + LII ins 2-3 (BB43) + LIIc R 3-4 p*Ubi*:*mCherry* + LII 4-6 dy (BB41)
Induction of *RAM2* promoter activity in noncolonized roots	LIIIβ F A-B p*Ubi*:*RAM1*_ p*RAM2*:*GUS* (pMP517)	Assembled by BpiI cut ligation from:<br>LIIc F 1-2 p*Ubi*:*RAM1* + LII ins 2-3 (BB43) + LIIβ F 3-4 p*RAM2*:*GUS* + LII ins 4-5 (BB44) + LIIβ F 5-6 p*Ubi*:*mCherry* + LIIIβ F A-B (BB53)

Induction of *RAM2* promoter activity in noncolonized roots	LIIIβ F A-B p*Ubi*:*RAM1*_ p*RAM2m*:*RAM2* (pMP518)	Assembled by BpiI cut ligation from: LIIc F 1-2 p*Ubi*:*RAM1* + LII ins 2-3 (BB43) + LIIβ F 3-4 p*RAM2m*:*GUS* + LII ins 4-5 (BB44) + LIIβ F 5-6 p*Ubi*:*mCherry* + LIIIβ F A-B (BB53)
Induction of *STR* promoter activity in noncolonized roots	LIIIβ F A-B p*Ubi*:*RAM1*_ p*STR*:*GUS* (pMP519)	Assembled by BpiI cut ligation from: LIIc F 1-2 p*Ubi*:*RAM1* + LII ins 2-3 (BB43) + LIIβ F 3-4 p*STR*:*GUS* + LII ins 4-5 (BB44) + LIIβ F 5-6 p*Ubi*:*mCherry* + LIIIβ F A-B (BB53)
Induction of *STR* promoter activity in noncolonized roots	LIIIβ F A-B p*Ubi*:*RAM1*_ p*STRm*:*GUS* (pMP520)	Assembled by BpiI cut ligation from: LIIc F 1-2 p*Ubi*:*RAM1* + LII ins 2-3 (BB43) + LIIβ F 3-4 p*STRm*:*GUS* + LII ins 4-5 (BB44) + LIIβ F 5-6 p*Ubi*:*mCherry* + LIIIβ F A-B (BB53)
Overexpression of *DELLA1*$^{\Delta 17}$	LIIIβ F A-B p*35S: DELLA1*$^{\Delta 17}$ (pPPLIII7)	(14)
Overexpression of *CCaMK*^{314}	LIIIβ F A-B p*Ubi*:*CCaMK*^{314} (pPPLIII15)	(14)
Transactivation assay	LIIIβ F A-B p*Ubi*:*Myc-RAM1m1* (pKH318)	Assembled by BpiI cut ligation from: LIIβ F 1-2 p*Ubi*:*Myc-RAM1m1* + LII ins 2-3 (BB43) + LIIc R 3-4 p*35S*:*mCherry* + LII ins 4-6 (BB41) + LIIIβ F A-B (BB53)
Transactivation assay	LIIIβ F A-B p*Ubi*:*Myc-RAM1m2* (pKH319)	Assembled by BpiI cut ligation from: LIIβ F 1-2 p*Ubi*:*Myc-RAM1m2* + LII ins 2-3 (BB43) + LIIc R 3-4 p*35S*:*mCherry* + LII ins 4-6 (BB41) + LIIIβ F A-B (BB53)
Transactivation assay	LIIIβ F A-B p*Ubi*:*Myc-RAM1m3* (pKH320)	Assembled by BpiI cut ligation from: LIIβ F 1-2 p*Ubi*:*Myc-RAM1m3* + LII ins 2-3 (BB43) + LIIc R 3-4 p*35S*:*mCherry* + LII ins 4-6 (BB41) + LIIIβ F A-B (BB53)
BiFC in hairy roots	LIIIβ fin p*RAM1*:*RAM1-nCit* p*CBX1*:*CBX1-cCit* (pSHL_3-32)	Assembled by BpiI cut ligation from: LIIc F 1-2 p*SbtM1*:*SP-mCherry* +LIIc F 2-3 ins (BB44)+ LIIβ F 3-4 p*RAM1*:*RAM1-nCit* + LIIc F 3-4 ins (BB44) + LIIβ F 5-6 p*CBX1*:*CBX1-cCit* + LIIIβ fin (BB52)
BiFC in hairy roots	LIIIβ fin p*RAM1*:*RAM1-nCit* p*WRI5b*:*WRI5b-cCit* (pSHL_3-35)	Assembled by BpiI cut ligation from: LIIc F 1-2 p*SbtM1*:*SP-mCherry* +LIIc F 2-3 ins (BB44)+ LIIβ F 3-4 p*RAM1*:*RAM1-nCit* + LIIc F 3-4 ins (BB44) +LIIβ F 5-6 p*WRI5b*:*WRI5b-cCit* + LIIIβ fin (BB52)
BiFC in hairy roots	LIIIβ fin p*RAM1*:*RAM1-nCit* p*CBX1*:*CBX1m4-cCit* (pSHL_3-33)	Assembled by BpiI cut ligation from: LIIc F 1-2 p*SbtM1*:*SP-mCherry* +LIIc F 2-3 ins (BB44)+ LIIβ F 3-4 p*RAM1*:*RAM1-nCit* + LIIc F 3-4 ins

		(BB44) + LIIβ F 5-6 p*CBX1*:*CBX1m4-cCit* + LIIIβ fin (BB52)
BiFC in hairy roots	LIIIβ fin p*RAM1*:*RAM1-nCit* p*CBX1*:*CBX1m7-cCit* (pSHL_3-34)	Assembled by BpiI cut ligation from: LIIc F 1-2 p*SbtM1*:*SP-mCherry* +LIIc F 2-3 ins (BB44)+ LIIβ F 3-4 p*RAM1*:*RAM1-nCit* + LIIc F 3-4 ins (BB44) + LIIβ F 5-6 p*CBX1*:*CBX1m7-cCit* + LIIIβ fin (BB52)
BiFC in hairy roots	LIIIβ fin p*RAM1*:*RAM1m3-nCit* p*CBX1*:*CBX1-cCit* (pSHL_3-36)	Assembled by BpiI cut ligation from: LIIc F 1-2 p*SbtM1*:*SP-mCherry* +LIIc F 2-3 ins (BB44)+LIIβ F 3-4 p*RAM1*:*RAM1m3-nCit* + LIIc F 3-4 ins (BB44) + LIIβ F 5-6 pCBX1:*CBX1-cCit* + LIIIβ fin (BB52)
BiFC in hairy roots	LIIIβ fin p*RAM1*:*RAM1m3-nCit* p*WRI5b*:*WRI5b-cCit* (pSHL_3-37)	Assembled by BpiI cut ligation from: LIIc F 1-2 p*SbtM1*:*SP-mCherry* +LIIc F 2-3 ins (BB44)+ LIIβ F 3-4 p*RAM1*:*RAM1m3-nCit* + LIIc F 3-4 ins (BB44) +LIIβ F 5-6 p*WRI5b*:*WRI5b-cCit* + LIIIβ fin (BB52)
CO-IP	LIIIβ fin p*Ubi*:*CBX1-GFP*	Assembled by BpiI cut ligation from: LII dy 1-2 (BB63) + LII ins 2-3 (BB44) + LIIb F3-4 p*Ubi*:*CBX1-GFP* + LII ins 4-5 (BB43) + LIIβ F 5-6 p*AtUBI10*:*mCherry* + LIIIβ fin (BB52)
CO-IP	LIIIβ fin p*Ubi*:*WRI5b-GFP*	Assembled by BpiI cut ligation from: LIIβ F 1-2 p*Ubi*:*WRI5b-GFP* + LII ins 2-3 (BB44) + LIIβ F 3-4 dy (BB64) + LII ins 4-5 (BB43) + LIIβ F 5-6 p*AtUBI10*:*mCherry* + LIIIβ fin (BB52)

CO-IP	LIIIβ fin p*Ubi*:*Myc-RAM1*	Assembled by BpiI cut ligation from: LIIβ F 1-2 p*Ubi*:*Myc-RAM1* + LII ins 2-3 (BB44) + LIIβ F 3-4 dy (BB64) + LII ins 4-5 (BB43) + LIIβ F 5-6 p*AtUBI10*:*mCherry* + LIIIβ fin (BB52)
CO-IP	LIIIβ F 1-2 p*Ubi*:*Myc-DELLA1*$^{\square 17}$ (pPPLIII22)	Assembled by BpiI cut ligation from: LIIc F 1-2 p*Ubi*:*Myc-DELLA1*$^{\square 17}$ + LII ins 2-3 (BB44) + LIIc R 5-6 p*35S*:*mCherry* + LII dy 4-6 (BB41) + LIIIβ F 1-2 (BB53)
CO-IP	LIIIβ fin p*35S*:*GFP* (pSHL_3-73)	Assembled by BpiI cut ligation from: LIIβ F 1-2 p*Ubi*:*mCherry* + LII ins 2-3 (BB44) + LII dy 3-4 (BB64) + LII ins 4-5 (BB43) + LIIc R 5-6 p*35S*:*GFP* + LIIIβ fin (BB52)
Complementation	LIIIβ fin p*RAM1*:*HA-RAM1*	Assembled by BpiI cut ligation from: LIIβ dy 1-2 (BB63) + LII ins 2-3 (BB44) + LIIβ F 3-4 p*RAM1*:*HA-RAM1* + LII ins 4-5 (BB43) + LIIβ F 5-6 p*AtUBI10*:*mCherry* + LIIIβ fin (BB52)
Complementation	LIIIβ fin p*RAM1*:*HA-RAM1m2*	Assembled by BpiI cut ligation from: LIIβ F 1-2 p*RAM1*:*HA-RAM1m2* + LII ins 2-3 (BB44) + LIIc F3-4 dy (BB64) + LII ins 4-5 (BB43) + LIIβ F 5-6 p*AtUBI10:mCherry* + LIIIβ fin (BB52)

gateway entry vectors		
	BB4 pENTR *WRI3.1* (pMP150)	Assembled by BsaI cut ligation from: BB4 pENTR + LI C-D *cWRI3.1*
	BB4 pENTR *WRI3.2* (pMP151)	Assembled by BsaI cut ligation from: BB4 pENTR + LI C-D *cWRI3.2*
	BB4 pENTR *CBX1* (pMP152)	Assembled by BsaI cut ligation from: BB4 pENTR + LI C-D *cCBX1*
	BB4 pENTR *WRI5a* (pMP147)	Assembled by BsaI cut ligation from: BB4 pENTR + LI C-D *cWRI5a*
	BB4 pENTR *WRI5b* (pMP148)	Assembled by BsaI cut ligation from: BB4 pENTR + LI C-D *cWRI5b*
	BB4 pENTR *WRI5c* (pMP149)	Assembled by BsaI cut ligation from: BB4 pENTR + LI C-D *cWRI5c*
	BB4 pENTR *RAM1* (pPPGW29)	Assembled by BsaI cut ligation from: BB4 pENTR + LI C-D *cRAM1*
	BB4 pENTR *RAM1-F1* (pPPGW37)	Assembled by BsaI cut ligation from: BB4 pENTR + LI C-D *cRAM1-F1*
	BB4 pENTR *RAM1-M5* (pPPGW60)	Assembled by BsaI cut ligation from: BB4 pENTR + LI C-D *cRAM1-M5*
	BB4 pENTR *CBX1_N* (pMP153)	Assembled by BsaI cut ligation from: BB4 pENTR + PCR amplicon MP276a + MP474 amplified from LI C-D *cCBX1*
	BB4 pENTR *CBX1_C* (pMP155)	Assembled by BsaI cut ligation from: BB4 pENTR + PCR amplicon MP473 + MP276b amplified from LI C-D *cCBX1*
	BB4 pENTR *CBX1_AP2* (pMP154)	Assembled by BsaI cut ligation from: BB4 pENTR + PCR amplicon MP471 + MP472 amplified from LI C-D *cCBX1*
	BB4 pENTR *CBX1_N+AP2* (pMP156)	Assembled by BsaI cut ligation from: BB4 pENTR + PCR amplicon MP276a + MP472 amplified from LI C-D *cCBX1*
	BB4 pENTR *CBX1_AP2+C* (pMP157)	Assembled by BsaI cut ligation from: BB4 pENTR + PCR amplicon MP471 + MP276b amplified from LI C-D *cCBX1*
	BB4 pENTR *CBX1_CΔ1* (pALGW40)	Assembled by BsaI cut ligation from: BB4 pENTR + PCR amplicon AL24 + MP276b amplified from LI C-D *cCBX1*
	BB4 pENTR *CBX1_CΔ2* (pALGW41)	Assembled by BsaI cut ligation from: BB4 pENTR + PCR amplicon AL26 + MP276b amplified from LI C-D *cCBX1*
	BB4 pENTR *CBX1_CΔ3* (pALGW42)	Assembled by BsaI cut ligation from: BB4 pENTR + PCR amplicon AL28 + MP276b amplified from LI C-D *cCBX1*
	BB4 pENTR *CBX1_CΔ4* (pALGW43)	Assembled by BsaI cut ligation from: BB4 pENTR + PCR amplicon AL30 + MP276b amplified from LI C-D *cCBX1*

	BB4 pENTR *RAM1-M5 ΔN1* (pMP127)	Assembled by BsaI cut ligation from: BB4 pENTR + PCR amplicon MP227 + MP226 amplified from pGAD424 *RAM1*
	BB4 pENTR *RAM1-M5 ΔN2* (pMP128)	Assembled by BsaI cut ligation from: BB4 pENTR + PCR amplicon MP228 + MP226 amplified from pGAD424 *RAM1*
	BB4 pENTR *RAM1-M5 ΔN3* (pMP129)	Assembled by BsaI cut ligation from: BB4 pENTR + PCR amplicon MP229 + MP226 amplified from pGAD424 *RAM1*
	BB4 pENTR *RAM1-M5 ΔN4* (pMP130)	Assembled by BsaI cut ligation from: BB4 pENTR + PCR amplicon MP230 + MP226 amplified from pGAD424 *RAM1*
	BB4 pENTR *RAM1-M5 ΔC1* (pMP131)	Assembled by BsaI cut ligation from: BB4 pENTR + PCR amplicon MP225 + MP231 amplified from pGAD424 *RAM1*
	BB4 pENTR *RAM1-M5 ΔC2* (pMP132)	Assembled by BsaI cut ligation from: BB4 pENTR + PCR amplicon MP225 + MP232 amplified from pGAD424 *RAM1*
	BB4 pENTR *RAM1-M5 ΔC3* (pMP133)	Assembled by BsaI cut ligation from: BB4 pENTR + PCR amplicon MP225 + MP233 amplified from pGAD424 *RAM1*
	BB4 pENTR *RAM1-M5 ΔC4* (pMP134)	Assembled by BsaI cut ligation from: BB4 pENTR + PCR amplicon MP225 + MP234 amplified from pGAD424 *RAM1*
	BB4 pENTR *RAM1m1* (pKH312)	Assembled by BsaI cut ligation from: BB4 pENTR + PCR amplicons PP5 + KH314, KH315 + PP57 amplified from pGAD424 *RAM1*

	BB4 pENTR *RAM1m2* (pKH313)	Assembled by BsaI cut ligation from: BB4 pENTR + PCR amplicons PP5 + KH316, KH317 + KH318, KH319 + PP57 amplified from pGAD424 *RAM1*
	BB4 pENTR *RAM1m3* (pKH314)	Assembled by BsaI cut ligation from: BB4 pENTR + PCR amplicons PP5 + KH320, KH321 + KH322, KH323 + KH324, KH325 + PP57 amplified from pGAD424 *RAM1*
	BB4 pENTR *CBX1m1* (pMP166)	Assembled by BsaI cut ligation from: BB4 pENTR + LI C-D *cCBX1m1*
	BB4 pENTR *CBX1m2* (pMP167)	Assembled by BsaI cut ligation from: BB4 pENTR + LI C-D *cCBX1m2*
	BB4 pENTR *CBX1m3* (pMP168)	Assembled by BsaI cut ligation from: BB4 pENTR + LI C-D *cCBX1m3*
	BB4 pENTR *CBX1m4* (pMP169)	Assembled by BsaI cut ligation from: BB4 pENTR + LI C-D *cCBX1m4*
	BB4 pENTR *CBX1m5* (pMP170)	Assembled by BsaI cut ligation from: BB4 pENTR + LI C-D *cCBX1m5*
	BB4 pENTR *CBX1m6* (pMP171)	Assembled by BsaI cut ligation from: BB4 pENTR + LI C-D *cCBX1m6*
	BB4 pENTR *CBX1m7* (pMP172)	Assembled by BsaI cut ligation from: BB4 pENTR + LI C-D *cCBX1m7*

gateway expression plasmids		
Transactivation assay	pAMPAT*p35S:3xHA-CYCLOPS*	(14)
Transactivation assay	*Kinase-RFP (CCaMK^{314})+NLS* (pK7WGR2)	(14)
EV for Y2H	pGAD424 (pMP700)	
EV for Y2H	pBD-GAL4 Cam (pMP702)	
Y2H	pGAD424 *WRI3.1* (pMP782)	LR clonase recombination of pGAD424 + BB4 pENTR *WRI3.1*
Y2H	pBD-GAL4 Cam *WRI3.1* (pMP783)	LR clonase recombination of pBD-GAL4 Cam + BB4 pENTR *WRI3.1*
Y2H	pGAD424 *WRI3.2* (pMP784)	LR clonase recombination of pGAD424 + BB4 pENTR *WRI3.2*
Y2H	pBD-GAL4 Cam *WRI3.2* (pMP785)	LR clonase recombination of pBD-GAL4 Cam + BB4 pENTR *WRI3.2*
Y2H	pGAD424 *CBX1* (pMP786)	LR clonase recombination of pGAD424 + BB4 pENTR *CBX1*
Y2H	pBD-GAL4 Cam *CBX1* (pMP787)	LR clonase recombination of pBD-GAL4 Cam + BB4 pENTR *CBX1*
Y2H	pGAD424 *WRI5a* (pMP773)	LR clonase recombination of pGAD424 + BB4 pENTR *WRI5a*
Y2H	pBD-GAL4 Cam *WRI5a* (pMP774)	LR clonase recombination of pBD-GAL4 Cam + BB4 pENTR *WRI5a*
Y2H	pGAD424 *WRI5b* (pMP776)	LR clonase recombination of of pGAD424 + BB4 pENTR *WRI5b*
Y2H	pBD-GAL4 Cam *WRI5b* (pMP777)	LR clonase recombination of pBD-GAL4 Cam + BB4 pENTR *WRI5b*
Y2H	pGAD424 *WRI5c* (pMP779)	LR clonase recombination of of pGAD424 + BB4 pENTR *WRI5c*
Y2H	pBD-GAL4 Cam *WRI5c* (pMP780)	LR clonase recombination of pBD-GAL4 Cam + BB4 pENTR *WRI5c*
Y2H	pGAD424 *RAM1* (pPPGW49)	LR clonase recombination of of pGAD424 + BB4 pENTR *RAM1*
Y2H	pBD-GAL4 Cam *RAM1* (pPPGW54)	LR clonase recombination of pBD-GAL4 Cam + BB4 pENTR *RAM1*
Y2H	pGAD424 *RAM1-F1* (pPPGW52)	LR clonase recombination of of pGAD424 + BB4 pENTR *RAM1-F1*
Y2H	pBD-GAL4 Cam *RAM1-F1* (pPPGW57)	LR clonase recombination of pBD-GAL4 Cam + BB4 pENTR *RAM1-F1*
Y2H	pGAD424 *RAM1-M5* (pPPGW66)	LR clonase recombination of of pGAD424 + BB4 pENTR *RAM1-M5*
Y2H	pBD-GAL4 Cam *RAM1-M5* (pPPGW72)	LR clonase recombination of pBD-GAL4 Cam + BB4 pENTR *RAM1-M5*
Y2H	pGAD424 *DELLA1* (pPPGW5)	(14)
Y2H	pBD-Gal4 *CBX1_N* (pMP788)	LR clonase recombination of pBD-GAL4 Cam + BB4 pENTR *CBX1_N*
Y2H	pBD-Gal4 *CBX1_C* (pMP792)	LR clonase recombination of pBD-GAL4 Cam + BB4 pENTR *CBX1_C*
Y2H	pBD-Gal4 *CBX1_AP2* (pMP790)	LR clonase recombination of pBD-GAL4 Cam + BB4 pENTR *CBX1_AP2*
Y2H	pBD-Gal4 *CBX1_N+AP2* (pMP789)	LR clonase recombination of pBD-GAL4 Cam + BB4 pENTR *CBX1_N+AP2*
Y2H	pBD-Gal4 *CBX1_AP2+C* (pMP791)	LR clonase recombination of pBD-GAL4 Cam + BB4 pENTR *CBX1_AP2+C*

Y2H	pBD-Gal4 *CBX1_CΔ1* (pALGW44)	LR clonase recombination of pBD-GAL4 Cam + BB4 pENTR *CBX1_CΔ1*
Y2H	pBD-Gal4 *CBX1_CΔ2* (pALGW45)	LR clonase recombination of pBD-GAL4 Cam + BB4 pENTR *CBX1_CΔ2*
Y2H	pBD-Gal4 *CBX1_CΔ3* (pALGW46)	LR clonase recombination of pBD-GAL4 Cam + BB4 pENTR *CBX1_CΔ3*
Y2H	pBD-Gal4 *CBX1_CΔ4* (pALGW47)	LR clonase recombination of pBD-GAL4 Cam + BB4 pENTR *CBX1_CΔ4*
Y2H	pGAD424 *RAM1-M5 ΔN1* (pMP733)	LR clonase recombination of of pGAD424 + BB4 pENTR *RAM1-M5 ΔN1*
Y2H	pGAD424 *RAM1-M5 ΔN2* (pMP734)	LR clonase recombination of of pGAD424 + BB4 pENTR *RAM1-M5 ΔN2*
Y2H	pGAD424 *RAM1-M5 ΔN3* (pMP733)	LR clonase recombination of of pGAD424 + BB4 pENTR *RAM1-M5 ΔN3*
Y2H	pGAD424 *RAM1-M5 ΔN4* (pMP735)	LR clonase recombination of of pGAD424 + BB4 pENTR *RAM1-M5 ΔN4*
Y2H	pGAD424 *RAM1-M5 ΔC1* (pMP736)	LR clonase recombination of of pGAD424 + BB4 pENTR *RAM1-M5 ΔC1*
Y2H	pGAD424 *RAM1-M5 ΔC2* (pMP737)	LR clonase recombination of of pGAD424 + BB4 pENTR *RAM1-M5 ΔC2*
Y2H	pGAD424 *RAM1-M5 ΔC3* (pMP738)	LR clonase recombination of of pGAD424 + BB4 pENTR *RAM1-M5 ΔC3*
Y2H	pGAD424 *RAM1-M5 ΔC4* (pMP739)	LR clonase recombination of of pGAD424 + BB4 pENTR *RAM1-M5 ΔC4*
Y2H	pGAD424 *RAM1m1* (pSHL_GW4)	LR clonase recombination of of pGAD424 + BB4 pENTR *RAM1m1*
Y2H	pGAD424 *RAM1m2* (pSHL_GW5)	LR clonase recombination of of pGAD424 + BB4 pENTR *RAM1m2*
Y2H	pGAD424 *RAM1m3* (pSHL_GW6)	LR clonase recombination of of pGAD424 + BB4 pENTR *RAM1m3*
Y2H	pBD-Gal4 *CBX1m1* (pMP810)	LR clonase recombination of of pBD-Gal4 + BB4 pENTR *CBX1m1*
Y2H	pBD-Gal4 *CBX1m2* (pMP811)	LR clonase recombination of of pBD-Gal4 + BB4 pENTR *CBX1m2*
Y2H	pBD-Gal4 *CBX1m3* (pMP812)	LR clonase recombination of of pBD-Gal4 + BB4 pENTR *CBX1m3*
Y2H	pBD-Gal4 *CBX1m4* (pMP813)	LR clonase recombination of of pBD-Gal4 + BB4 pENTR *CBX1m4*
Y2H	pBD-Gal4 *CBX1m5* (pMP814)	LR clonase recombination of of pBD-Gal4 + BB4 pENTR *CBX1m5*
Y2H	pBD-Gal4 *CBX1m6* (pMP815)	LR clonase recombination of of pBD-Gal4 + BB4 pENTR *CBX1m6*
Y2H	pBD-Gal4 *CBX1m7* (pMP816)	LR clonase recombination of of pBD-Gal4 + BB4 pENTR *CBX1m7*

Table S6. Benchmark data of the WRI amino acid sequences used for phylogenetic analysis.

plant species	AM host	sequences retrieved from	number of sequences	Number of sequences with M2/M2b or M2like motif	*RAM1* present in genome
Amaranthus hypochondriacus	No	TFDB	14	0	No
Arabidopsis thaliana	No	TAIR, NCBI	19	0	No
Beta vulgaris	No	TFDB, NCBI	13	0	No
Brachypodium distachyon	Yes	TFDB, NCBI	25	5	Yes
Brassica napus	No	TFDB, NCBI	57	0	No
Camelina sativa	No	TFDB, NCBI	56	0	No
Cannabis sativa	Yes	EnsemblPlants, NCBI	17	4	Yes
Capsella rubella	No	TFDB, NCBI	18	0	No
Cephalotus follicularis	No	NCBI	14	1	No
Daucus carota	Yes	TFDB, NCBI	29	5	Yes (2x)
Dianthus caryophyllus	No	TFDB, NCBI	11	0	No
Eutrema salsugineum	No	TFDB, NCBI	18	0	No
Fragaria vesca	Yes	TFDB, NCBI	18	4	Yes
Glycine max	Yes	TFDB, NCBI	48	8	Yes (2x)
Helianthus annuus	Yes	EnsemblPlants, NCBI	35	6	Yes (2x)
Lotus japonicus Gifu	Yes	Lotusbase	20	6	Yes
Lupinus angustifolius	No	EnsemblPlants, NCBI	33	2	No
Manihot esculenta	Yes	TFDB, NCBI	29	7	Yes
Marchantia polymorpha	No	TFDB, NCBI	2	1	No
Medicago truncatula	Yes	TFDB	27	6	Yes
Nelumbo nucifera	No	TFDB, NCBI	18	2	No
Nicotiana benthamiana	Yes	TFDB	39	4	Yes
Nymphea colorata	No	EnsemblPlants, NCBI	13	1	No
Oryza sativa japonica	yes	TFDB	21	3	yes
Petunia axillaris	Yes	TFDB, NCBI	26	5	Yes
Physcomitrium patens	No	TFDB, NCBI	10	2	No
Picea abies	No	TFDB, NCBI	9	1	No
Populus trichocarpa	Yes	TFDB, NCBI	29	6	Yes
Selaginella moellendorffii	Yes	TFDB, NCBI	7	2	Yes
Setaria italica	Yes	TFDB, NCBI	28	4	Yes
Sisymbrium irio	No	TFDB	18	0	No
Solanum lycopersicum	Yes	TFDB, NCBI	23	4	Yes
Spinacia oleracera	No	TFDB, NCBI	13	0	No
Striga asiatica	No	NCBI	17	0	No
Utricularia gibba	No	TFDB	23	1	No
Vitis vinifera	Yes	TFDB, NCBI	20	4	Yes
Zea mays	Yes	TFDB, NCBI	28	4	Yes
Zostera marina	no	TFDB, NCBI	17	0	no

Supplementary References

1. P. Pimprikar, *et al.*, A CCaMK-CYCLOPS-DELLA complex activates transcription of *RAM1* to regulate arbuscule branching. *Curr. Biol.* **26**, 987–998 (2016).
2. S. Singh, K. Katzer, J. Lambert, M. Cerri, M. Parniske, CYCLOPS, a DNA-binding transcriptional activator, orchestrates symbiotic root nodule development. *Cell Host Microbe* **15**, 139–152 (2014).
3. A. Keymer, *et al.*, Lipid transfer from plants to arbuscular mycorrhiza fungi. *Elife* **6**, e29107–e29107 (2017).
4. L. Xue, *et al.*, AP2 transcription factor CBX1 with a specific function in symbiotic exchange of nutrients in mycorrhizal *Lotus japonicus*. *Proc. Natl. Acad. Sci. U. S. A.* **115**, E9239–E9246 (2018).
5. J. Perry, *et al.*, TILLING in *Lotus japonicus* identified large allelic series for symbiosis genes and revealed a bias in functionally defective ethyl methanesulfonate alleles toward glycine replacements. *Plant Physiol.* **151**, 1281–1291 (2009).
6. T. Zeng, *et al.*, Host-and stage-dependent secretome of the arbuscular mycorrhizal fungus *Rhizophagus irregularis*. *Plant J.* **94**, 411–425 (2018).
7. H. Vierheilig, A. P. Coughlan, U. R. S. Wyss, Y. Piché, Ink and vinegar, a simple staining technique for arbuscular-mycorrhizal fungi. *Appl. Environ. Microbiol.* **64**, 5004–5007 (1998).
8. S. Torabi, K. Varshney, J. A. Villaécija-Aguilar, A. Keymer, C. Gutjahr, "Controlled assays for phenotyping the effects of strigolactone-like molecules on arbuscular mycorrhiza development" in *Strigolactones*, (Springer, 2021), pp. 157–177.
9. T. P. McGonigle, M. H. Miller, D. G. Evans, G. L. Fairchild, J. A. Swan, A new method which gives an objective measure of colonization of roots by vesicular—arbuscular mycorrhizal fungi. *New Phytol.* **115**, 495–501 (1990).
10. N. Panchuk-Voloshina, *et al.*, Alexa dyes, a series of new fluorescent dyes that yield exceptionally bright, photostable conjugates. *J. Histochem. Cytochem.* **47**, 1179–1188 (1999).
11. S. Rozen, H. Skaletsky, "Primer3 on the WWW for general users and for biologist programmers" in *Bioinformatics Methods and Protocols*, (Springer, 2000), pp. 365–386.
12. T. Czechowski, R. P. Bari, M. Stitt, W. Scheible, M. K. Udvardi, Real-time RT-PCR profiling of over 1400 *Arabidopsis* transcription factors: unprecedented sensitivity reveals novel root-and shoot-specific genes. *Plant J.* **38**, 366–379 (2004).
13. A. Binder, *et al.*, A modular plasmid assembly kit for multigene expression, gene silencing and silencing rescue in plants. *PLoS One* **9**, e88218–e88218 (2014).
14. N. Takeda, S. Sato, E. Asamizu, S. Tabata, M. Parniske, Apoplastic plant subtilases support arbuscular mycorrhiza development in *Lotus japonicus*. *Plant J.* **58**, 766–777 (2009).
15. M. Baek, *et al.*, Accurate prediction of protein structures and interactions using a three-track neural network. *Science.* **373**, 871–876 (2021).
16. M. Mirdita, *et al.*, ColabFold: making protein folding accessible to all. *Nat. Methods* **19**, 679–682 (2022).
17. L. Schrödinger, W. DeLano, The PyMOL molecular graphics system, version 2.0 Schrödinger, LLC (2017).
18. T. Mun, A. Bachmann, V. Gupta, J. Stougaard, S. U. Andersen, Lotus Base: An integrated information portal for the model legume *Lotus japonicus*. *Sci. Rep.* **6**, 1–18 (2016).
19. J. Montiel, *et al.*, Distinct signaling routes mediate intercellular and intracellular rhizobial infection in Lotus japonicus. *Plant Physiol.* **185**, 1131–1147 (2021).
20. T. Z. Berardini, *et al.*, The *Arabidopsis* information resource: making and mining the "gold standard" annotated reference plant genome. *Genesis* **53**, 474–485 (2015).
21. J. Jin, *et al.*, PlantTFDB 4.0: toward a central hub for transcription factors and regulatory interactions in plants. *Nucleic Acids Res.*, gkw982–gkw982 (2016).
22. K. L. Howe, *et al.*, Ensembl Genomes 2020—enabling non-vertebrate genomic research. *Nucleic Acids Res.* **48**, D689–D695 (2020).
23. R. C. Edgar, MUSCLE: multiple sequence alignment with high accuracy and high

throughput. *Nucleic Acids Res.* **32**, 1792–1797 (2004).
24. M. Gouy, E. Tannier, N. Comte, D. P. Parsons, "Seaview version 5: a multiplatform software for multiple sequence alignment, molecular phylogenetic analyses, and tree reconciliation" in *Multiple Sequence Alignment*, (Springer, 2021), pp. 241–260.
25. V. Lefort, J.-E. Longueville, O. Gascuel, SMS: smart model selection in PhyML. *Mol. Biol. Evol.* **34**, 2422–2424 (2017).
26. S. Guindon, *et al.*, New algorithms and methods to estimate maximum-likelihood phylogenies: assessing the performance of PhyML 3.0. *Syst. Biol.* **59**, 307–321 (2010).
27. T. Müller, M. Vingron, Modeling amino acid replacement. *J. Comput. Biol.* **7**, 761–776 (2000).
28. M. Anisimova, O. Gascuel, Approximate likelihood-ratio test for branches: a fast, accurate, and powerful alternative. *Syst. Biol.* **55**, 539–552 (2006).
29. T. L. Bailey, C. Elkan, Fitting a mixture model by expectation maximization to discover motifs in bipolymers (1994).
30. T. L. Bailey, J. Johnson, C. E. Grant, W. S. Noble, The MEME suite. *Nucleic Acids Res.* **43**, W39–W49 (2015).
31. S. Gupta, J. A. Stamatoyannopoulos, T. L. Bailey, W. S. Noble, Quantifying similarity between motifs. *Genome Biol.* **8**, 1–9 (2007).
32. R. C. Team, R: A language and environment for statistical computing (2013).
33. Rs. Team, RStudio: Integrated Development for R. RStudio, PBC, Boston, MA URL (2020).
